# Screening of core targets for Di(2-ethylhexyl) Phthalate-related gastric cancer based on machine learning, molecular docking, and SHAP analysis

**DOI:** 10.1371/journal.pcbi.1014514

**Published:** 2026-07-16

**Authors:** Shenghao Li, Qing Peng, Liyuan Hao, Jingyu Mao, Bingjie Huo

**Affiliations:** 1 Department of Integrated Traditional Chinese and Western Medicine Oncology, The Fourth Hospital of Hebei Medical University, Shijiazhuang, Hebei, China; 2 School of Clinical Medicine, Chengdu University of Traditional Chinese Medicine, Chengdu, Sichuan, China; 3 Department of Infectious Diseases, Hospital of Chengdu University of Traditional Chinese Medicine, Chengdu, Sichuan, China; 4 College of Integrated Traditional Chinese and Western Medicine, Hebei University of Chinese Medicine, Shijiazhuang, Hebei, China; Children’s National Hospital, UNITED STATES OF AMERICA

## Abstract

**Purpose:**

Given the existing uncertainties regarding the link between Di(2-ethylhexyl) phthalate (DEHP) exposure and gastric cancer (GC) progression, this study aimed to clarify their association, identify the toxic targets of DEHP, and elucidate the underlying molecular mechanisms.

**Methods:**

Multiple integrated approaches were employed, including Gene Expression Omnibus (GEO) data analysis, network toxicology, molecular docking, and machine learning. STRING and Cytoscape tools were utilized to identify key targets, while Gene Ontology (GO) and Kyoto Encyclopedia of Genes and Genomes (KEGG) enrichment analyses were performed to explore the functional enrichment of intersecting targets. Machine learning and SHAP analysis were applied to screen core targets in GC. Molecular docking was performed to evaluate the binding affinity of DEHP toward core targets, and 200 ns molecular dynamics simulations were further conducted for representative complexes to validate their dynamic stability.

**Results:**

A total of 18 key targets were identified using STRING and Cytoscape. GO and KEGG enrichment analyses demonstrated that these intersecting targets were primarily enriched in the extracellular region, as well as the Calcium signaling pathway and cAMP signaling pathway. Through machine learning analyses, 7 key genes (*ADRB2*, *ESRRG*, *GRIA4*, *IL13RA2*, *NR3C2*, *PLA2G1B*, and *SULT2A1*) were identified as core targets in GC through machine learning analyses. Molecular docking simulations revealed strong binding specificity between DEHP and the target proteins. Among them, NR3C2 and ADRB2 exhibited relatively high predictive importance in the machine learning models. DEHP showed favorable binding affinity toward these core targets, and molecular dynamics simulations further confirmed that ADRB2–DEHP and NR3C2–DEHP complexes maintained stable conformations throughout the simulation.

**Conclusions:**

Our findings identified GC associated genes that were computationally predicted as potential targets of DEHP. These results indicated structural compatibility between DEHP and its target proteins but did not prove that DEHP exposure accounts for the gene expression changes in GC.

## Author summary

Di(2-ethylhexyl) phthalate (DEHP), a common plasticizer in food packaging and daily plastics, is a widespread environmental contaminant humans are exposed to regularly, but its association with gastric cancer (GC), one of the world’s most fatal malignancies, remains poorly understood. Here, we combined bioinformatics, machine learning, and molecular docking to explore the potential links between DEHP and GC development. Functional enrichment analysis suggested that these genes may disrupt key biological pathways including the calcium and cAMP signaling pathways. We further performed molecular docking, which revealed strong binding affinity between DEHP and the proteins encoded by these core targets. Notably, 200 ns MD simulations confirmed stable conformations of ADRB2–DEHP and NR3C2–DEHP complexes, supporting structural plausibility of binding. This confirmed only structural plausibility of binding and did not support a causal or associative link between DEHP exposure and the gene expression changes in GC, which reflect GC biology rather than DEHP exposure.

## Introduction

Integrated computational approaches combining bioinformatics, network toxicology, machine learning, and molecular docking have been widely recognized and applied in exploring the toxic molecular mechanisms of environmental pollutants and the pathogenic basis of malignant tumors [1–3]. Such studies collectively demonstrate that this integrated computational approach has become a mainstream and well-accepted tool in environmental toxicology and tumor biology research. Di(2-ethylhexyl) phthalate (DEHP) is a widely used plasticizer that enhances the flexibility of polyvinyl chloride (PVC) products [4]. It is often found in food packaging, plastic flooring, wrapping materials for wires and cables, and children’s toys [5]. The primary route of human exposure to DEHP is the ingestion of packaged food contaminated with this compound [5,6]. Because of its chemical makeup, DEHP tends to seep out of packaging materials and get into the food inside [5,6], leading to chronic low-dose exposure in the general population. Given the widespread application of DEHP and the persistent human exposure to it, significant concerns have been raised regarding its potential health hazards, primarily due to its diverse adverse effects.

Gastric cancer (GC) is one of the most prevalent malignant tumors worldwide, ranking fourth in mortality and fifth in incidence [7]. Phthalate exposure is associated with various potential adverse health effects in humans. Di(2-ethylhexyl) phthalate (DEHP) is a plasticizer widely present in food packaging [8]. Studies have indicated that even extremely low concentrations of DEHP can upregulate the expression of the pro-inflammatory gene cyclooxygenase-2 (COX-2) in GC cells by the activation of extracellular signal-regulated kinase 1/2 (ERK1/2) and nuclear factor-κB (NF-κB) [9]. Studies have shown that extremely low concentrations of DEHP exert a significant impact on cell migration by promoting the epithelial-mesenchymal transition (EMT) of GC cells. This effect was mediated by the regulation of the PI3K/AKT/mTOR and Smad2 signaling pathways [10]. Additionally, it has been discovered that DEHP can augment the cytotoxicity of Helicobacter pylori and trigger apoptosis in gastric epithelial cells [11], suggesting that DEHP may synergize with pathogenic bacterial infection to accelerate gastric mucosal damage and the development of precancerous lesions. Beyond GC, DEHP has also been implicated in the development of other digestive tract tumors. Specifically, long-term exposure to DEHP reduced the therapeutic efficacy of sorafenib by enhancing the mesenchymal transition of hepatocellular carcinoma (HCC) cells [12]. Studies have further revealed that DEHP exposure consistently upregulates Krüppel-like factor 5 (KLF5) and matrix metalloproteinase 7 (MMP7), and enhances the migration and invasion of pancreatic cancer PANC-1 cells [13]. Despite these findings, the core target genes and precise regulatory networks underlying DEHP‑related GC initiation and progression remain poorly elucidated, highlighting the need for systematic and in-depth investigation.

Network toxicology integrates multiple disciplines, including bioinformatics, big data analytics, and genomics, along with related technologies, to investigate the toxicity pathways of chemicals and the molecular mechanisms underlying diseases [14,15]. It exhibits the characteristics of high efficiency, user-friendliness, and comprehensiveness, and holds broad prospects in exploring the molecular mechanisms of toxicity [16], making it a powerful tool for identifying core toxic targets of environmental pollutants in malignant tumors. This approach facilitates the clarification of intricate relationships among disease-related and chemical-related genes, proteins, and metabolites [16].

**Fig 1:**
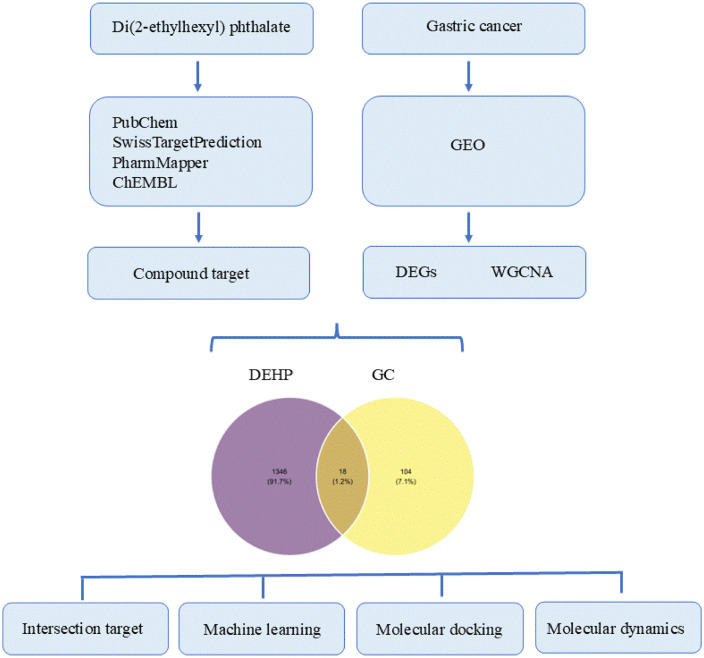
Flow chart of the study design.

In the present study, we analyzed data from the Gene Expression Omnibus (GEO) dataset to explore the association between DEHP exposure and GC. Moreover, we employed strategies including network toxicology, molecular docking, molecular dynamics simulation, and multi-level bioinformatics data analysis to decipher the complex interactions and molecular pathways linking DEHP to GC. By leveraging machine learning to prioritize key hub molecules through topological and functional enrichment analyses, coupled with molecular docking to verify target binding and interactions, 200 ns molecular dynamics simulations to further validate the dynamic stability of representative protein-ligand complexes, this study aimed to explore computationally predicted associations between DEHP and GC, identify potential binding targets, and generate mechanistic hypotheses for future experimental validation. No causal relationship between DEHP exposure and GC gene expression changes is directly demonstrated in this study (Fig 1).

## Results

### Acquisition of compound-related targets

**Fig 2:**
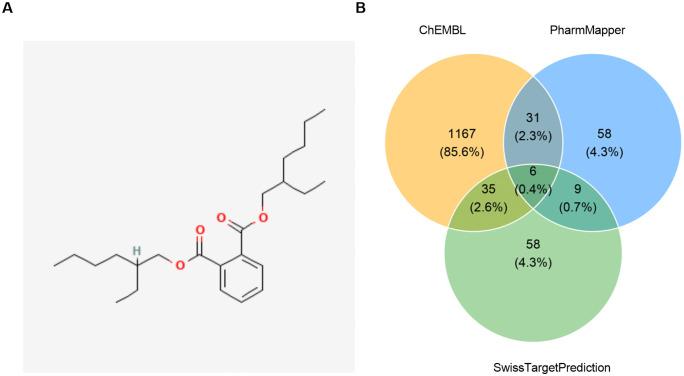
Identification of DEHP‑related candidate targets. A. Chemical structure of DEHP. B. Venn diagram of DEHP‑related targets from multiple databases.

To identify candidate targets associated with DEHP-related GC, the chemical structure of DEHP was first obtained from the PubChem database (Fig 2A). Then DEHP-related targets were systematically collected from three databases: 1,239 targets were retrieved from the ChEMBL database, 104 targets were predicted using the PharmMapper database, and 108 targets were predicted via the SwissTargetPrediction database. After integration and deduplication, a total of 1,364 putative DEHP-associated targets were obtained (Fig 2B), providing a target pool for subsequent screening.

### Acquisition of disease-related targets

**Fig 3:**
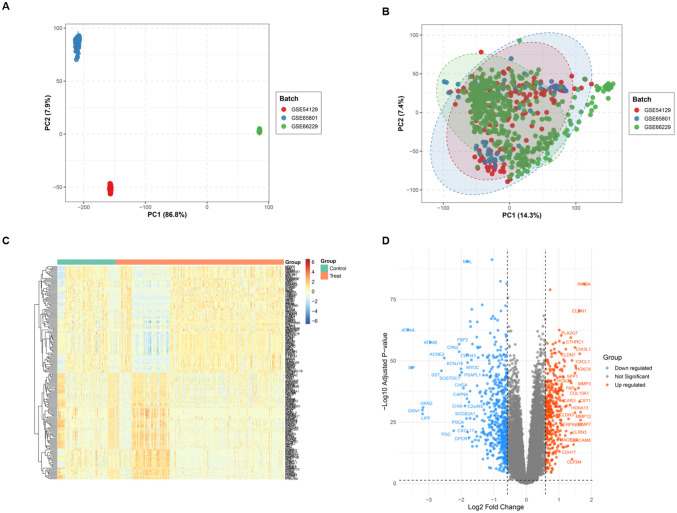
Acquisition of disease-related targets. A. PCA analysis before merging the three chip datasets. B. PCA analysis after merging the three chip datasets. C. The heatmap represented expression patterns of partial significantly up-regulated or down-regulated DEGs. D. The volcano plots represented the expression pattern of DEGs with red representing up-regulated genes, blue representing down-regulated genes, and gray representing genes with no significant differences.

To obtain disease-related targets, microarray datasets were downloaded from the GEO database. To expand the sample size and improve the accuracy of differentially expressed gene identification, three microarray datasets three microarray datasets (GSE66229, GSE65801, and GSE54129) were integrated for combined analysis. PCA results revealed that the pre-normalization dataset displayed obvious single-chip clustering, indicating severe batch effects. In contrast, following normalization, the integrated data showed a more compact clustering tendency with batch effects reduced (Fig 3A-B).

Using the normalized merged dataset, 863 differentially expressed genes (DEGs) were identified, including 356 upregulated and 507 downregulated genes. The expression profiles of these DEGs were visualized through a heatmap, which intuitively displayed the clustering of DEGs expression levels across samples, and a volcano plot, which illustrated the distribution of DEGs based on fold change and statistical significance (Fig 3C-D) (S1 Table).

### Acquisition of targets with significant disease-related changes

**Fig 4:**
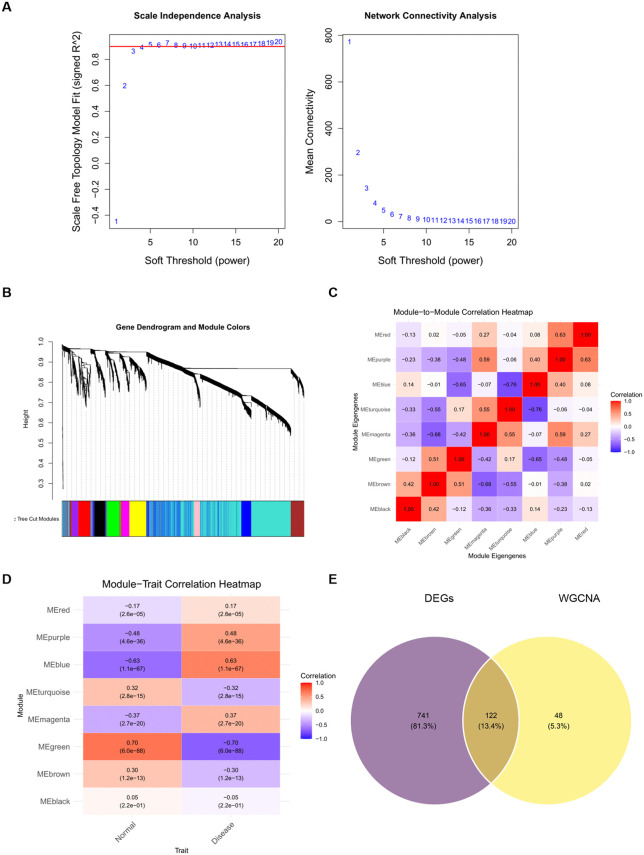
WGCNA analysis identified core modules. A. Analysis of the scale-free fit index for various soft-thresholding powers (β) and the mean connectivity for various soft-thresholding powers. B. The dendrogram obtained by clustering all genes. C. The module-to-module correlation heatmap shows the degree of correlation between gene modules. D. The heatmap shows that 8 modules are identified between normal samples and tumor samples. E. Venn diagram analysis of DEGs and WGCNA.

WGCNA was performed to identify co-expression modules associated with GC. In the left panel (scale independence analysis), the goodness-of-fit (R^2^) of the scale-free topological model increased with rising soft threshold and approached 0.8, gradually endowing the network with scale-free properties. Combined with network connectivity analysis, the network under this threshold not only met the fitting requirements of the scale-free structure but also maintained a reasonable average node connectivity, providing performance parameters for network construction (Fig 4A).

Clustering analysis of gene expression data grouped genes with similar expression patterns into distinct branches. Different colors represent different modules, each containing a set of co-expressed genes that may exert synergistic effects in specific biological processes or phenotypes (Fig 4B). A total of 8 co-expression modules were identified, with the gene number of each module as follows: MEblack (144 genes), MEbrown (494 genes), MEgreen (170 genes), MEmagenta (204 genes), MEblue (465 genes), MEturquoise (1514 genes), MEpurple (87 genes), and MEred (165 genes). The Module-to-Module Correlation Heatmap shows the correlation levels between the eigengenes of these gene modules (Fig 4C). Correlation values closer to 1 indicate stronger positive correlations between modules, while values closer to -1 indicate stronger negative correlations; values near 0 indicate insignificant associations. For instance, MEred showed a perfect positive correlation with itself (1.00) and a positive correlation with MEpurple (0.63), while MEblue exhibited a strong negative correlation with MEturquoise (-0.76), which intuitively reflects the interaction patterns between different modules.

The Module–trait correlation heatmap was constructed to quantify the correlation between each module eigengene and the disease phenotype, with both correlation coefficients and corresponding statistical *P*-values calculated (Fig 4D). The results showed that all modules were significantly correlated with the GC phenotype, among which the MEgreen module exhibited the strongest correlation with GC (correlation coefficient = -0.70, *P* = 6.0e-88), followed by the MEblue module (correlation coefficient = 0.63, *P* = 1.1e-67). Scatterplots of MM and GS were further generated to evaluate the performance of module-trait correlations, and the results confirmed a strong positive correlation between intramodular MM and GS for the MEgreen module, indicating that genes in this module had both high module specificity and strong phenotypic relevance. Based on the criteria of the strongest correlation with GC phenotype and the highest intramodular MM-GS correlation, the MEgreen module (170 genes) was selected as the key module for subsequent analysis.

Hub genes in the MEgreen module were screened using the criteria |MM| ≥ 0.8 and |GS| ≥ 0.2. By intersecting these genes with the 863 DEGs, 122 overlapping genes were identified (Fig 4E), which were regarded as candidate core dysregulated genes in GC (Fig 4E), suggesting that these genes are core co-expressed DEGs and may play key regulatory roles in the induction and progression of GC.

### Acquisition of Di(2-ethylhexyl) phthalate-associated gastric cancer-related targets

**Fig 5:**
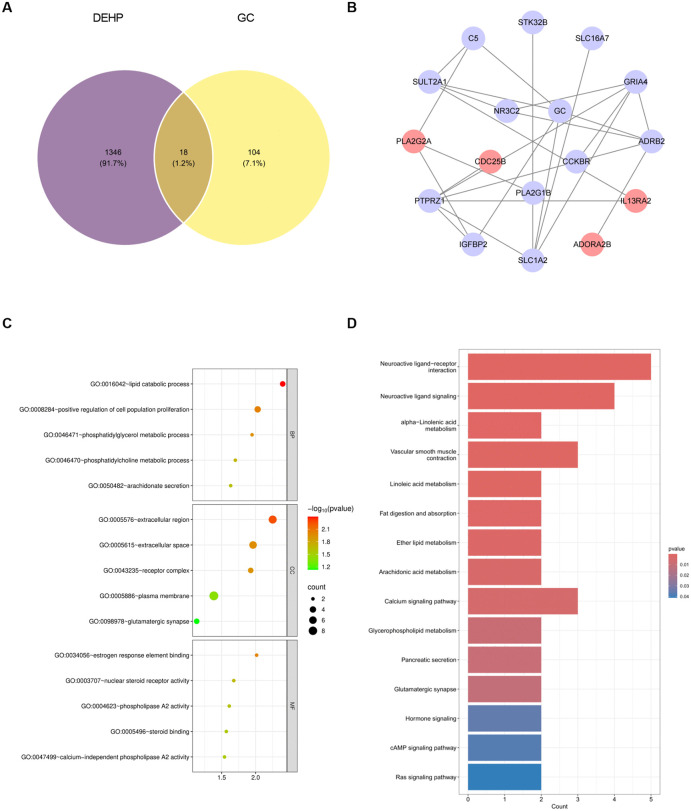
Identification of overlapping targets between DEHP and GC. A. Venn diagram of overlapping targets between DEHP and GC. B. PPI network of intersecting targets, with red representing up-regulation and purple representing down-regulation. C. GO analysis of the intersecting targets. D. KEGG analysis of the intersecting targets.

The 1,364 computationally predicted DEHP targets were intersected with the 122 GC-related candidate genes, yielding 18 overlapping genes (Fig 5A). These 18 genes were considered putative key targets linking DEHP exposure to GC. It should be noted that this represents a computational overlap rather than direct causal evidence. These 18 overlapping targets were imported into the STRING database to construct a PPI network, and the network was visualized using Cytoscape software (Fig 5B).

Subsequently, the 18 overlapping targets were imported into the DAVID database for GO enrichment analysis, and the results were visualized. In the BP category of GO analysis, the overlapping targets were mainly enriched in GO:0016042 ~ lipid catabolic process. In the CC category, they were primarily enriched in GO:0005576 ~ extracellular region. In the MF category, they were mainly enriched in GO:0034056 ~ estrogen response element binding (Fig 5C).

KEGG pathway analysis showed that the overlapping targets were mainly enriched in pathways such as vascular smooth muscle contraction, ether lipid metabolism, calcium signaling pathway, and cAMP signaling pathway (Fig 5D). These results suggested that metabolic reprogramming and multiple signaling pathways might be involved in DEHP-related GC.

### Identification of key targets in Di(2-ethylhexyl) phthalate-related gastric cancer

**Fig 6:**
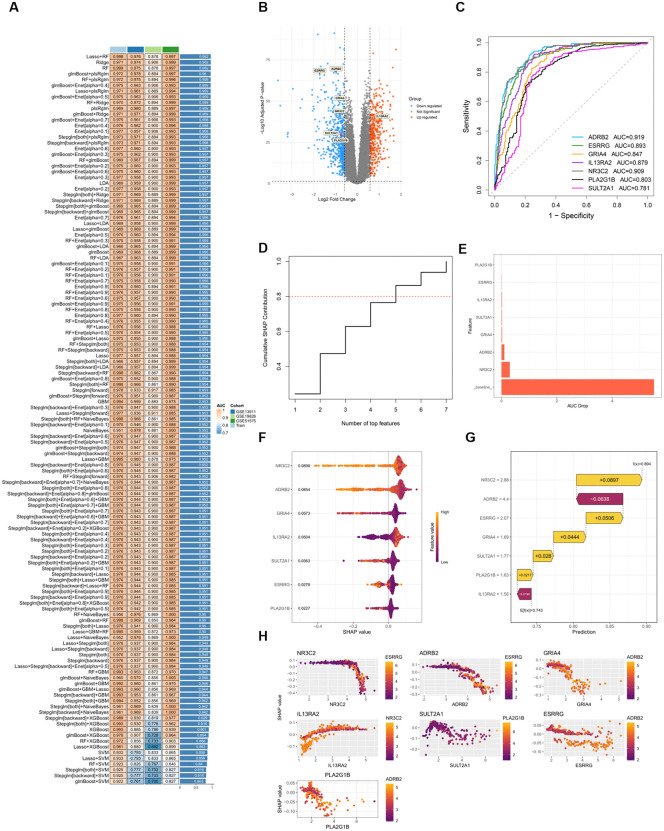
Machine learning in screening candidate diagnostic biomarkers. A. This heatmap presents the performance of different model combinations in relevant tasks. B. Volcano plot shows DEGs, with red representing up-regulation and blue representing down-regulation. C. ROC curves for key genes. D. Cumulative SHAP contribution curve of key targets. E. Permutation feature importance analysis of key targets. F. The SHAP summary plot shows the distribution of SHAP values for the top 7 genes, colored by feature value (yellow = high, purple = low), illustrating their impact on model prediction. G. The SHAP summary plot illustrates the contribution of genes to prediction results. H. SHAP value and associated categories for genes.

To identify candidate core genes associated with DEHP-related GC, machine learning models were constructed based on the 18 overlapping targets. High-performance models were defined as those containing 3–10 genes and achieving an AUC > 0.9 in both the training and validation cohorts. Among the 127 constructed models, the glmBoost+Enet [alpha = 0.5] model was selected as the optimal model (Fig 6A) (S2 Table). Based on this optimal model, 7 candidate core genes were ultimately identified: adrenoceptor Beta 2 (*ADRB2*), estrogen related receptor gamma (*ESRRG*), glutamate Ionotropic receptor AMPA type subunit 4 (*GRIA4*), interleukin 13 receptor subunit alpha 2 *(IL13RA2*), nuclear receptor subfamily 3 group C member 2 (*NR3C2*), phospholipase A2 group 1B (*PLA2G1B*), and sulfotransferase family 2A member 1 (*SULT2A1*). Differential expression analysis visualized in the volcano plot (Fig 6B) revealed that *ADRB2*, *ESRRG*, *GRIA4*, *NR3C2*, *PLA2G1B*, and *SULT2A1* were significantly downregulated in GC, whereas *IL13RA2* was markedly upregulated. ROC analysis confirmed their potential diagnostic value, with AUC values greater than 0.78 (Fig 6C).

To evaluate the contribution of each gene to model prediction, cumulative SHAP value distribution analysis was constructed. As shown in Fig 6D, the top three genes accounted for more than 80% of the total model importance, indicating that model prediction depended predominantly on a small subset of features. In line with this, permutation feature importance analysis (Fig 6E) demonstrated that permutation of *NR3C2* and *ADRB2* resulted in the largest reductions in AUC, underscoring their critical roles for model predictive performance.

*NR3C2* (SHAP value = 0.0898) and *ADRB2* (SHAP value = 0.0854) were the most impactful features for model prediction, followed by *GRIA4* (0.0573), *IL13RA2* (0.0540), *SULT2A1* (0.0363), *ESRRG* (0.0278), and *PLA2G1B* (0.0227) (Fig 6F). Further SHAP analysis revealed that an *ADRB2* expression value of 4.4 corresponded to a SHAP value of -0.0638, suggesting that elevated *ADRB2* expression was associated with a decreased predicted probability. Conversely, an *NR3C2* expression value of 2.88 corresponded to a SHAP value of 0.0897, indicating that higher *NR3C2* expression was linked to an increased predicted probability in this sample (Fig 6G).

As shown in Fig 6H, the SHAP value distribution across categories revealed that the predictive contributions of each gene varied substantially among GC subgroups. *ADRB2* and *GRIA4* exhibited both positive and negative SHAP values across multiple categories, indicating their divergent predictive effects and potential relevance to GC heterogeneity. In contrast, NR3C2, SULT2A1, and PLA2G1B predominantly displayed negative SHAP values, suggesting context-specific predictive patterns in specific categories. ESRRG showed context-dependent predictive effects across categories 2, 5, and 6, while IL13RA2 was mainly associated with negative predictive contributions in category 4. These findings emphasized that the predictive roles of the core genes were not uniform but depended on GC subtype context.

### Abnormal expression of the core target in gastric cancer

**Fig 7:**
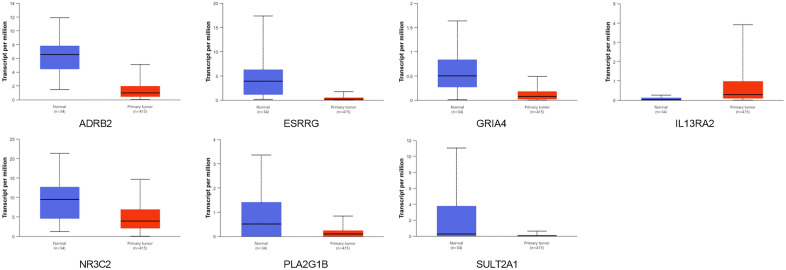
Abnormal expression of the core target in GC.

Furthermore, expression patterns of the 7 core genes were further validated using the UALCAN database. The results showed that *ADRB2*, *ESRRG*, *GRIA4*, *NR3C2*, *PLA2G1B*, and *SULT2A1* were downregulated, while *IL13RA2* was upregulated (Fig 7).

### Molecular docking

**Fig 8:**
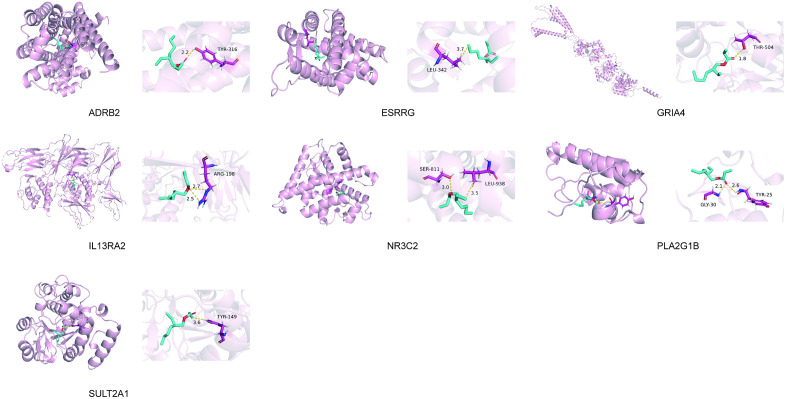
Molecular docking.

Molecular docking calculations were performed using AutoDock Vina software, and the optimal binding modes were visualized and analyzed using PyMOL software. The results showed that DEHP could form stable hydrogen bond interactions with 7 potential targets, namely ADRB2, ESRRG, GRIA4, IL13RA2, NR3C2, PLA2G1B, and SULT2A1. The details are as follows: in ADRB2, DEHP formed a hydrogen bond with TYR-316, with a bond length of 2.2 Å. In ESRRG, DEHP formed a weak hydrogen bond with LEU-342, with a bond length of 3.7 Å. In GRIA4, DEHP formed a hydrogen bond with THR-504, with a bond length of 1.8 Å. In IL13RA2, DEHP formed two hydrogen bonds with ARG-198, with bond lengths of 2.5 Å and 2.7 Å, respectively. In NR3C2, DEHP formed hydrogen bonds with SER-811 (3.0 Å) and LEU-938 (3.5 Å), respectively. In PLA2G1B, DEHP formed hydrogen bonds with GLY-30 (2.1 Å) and TYR-25 (2.6 Å). In SULT2A1, DEHP formed a hydrogen bond with TYR-149, with a bond length of 3.6 Å. All the above binding regions were located in the functionally active pocket regions of each protein, suggesting that DEHP may interact with the targets through specific binding sites, which may affect their biological functions (Table 1, Fig 8).

**Table 1:** Molecular docking binding energy results (kcal/mol).

Ligands	Receptors	Bind energy
DEHP	ADRB2 (6PS2)	-5.7
DEHP	ESRRG (2E2R)	-6.3
DEHP	GRIA4 (alphfold)	-4.6
DEHP	IL13RA2 (3LB6)	-4.4
DEHP	NR3C2 (4PF3)	-5.3
DEHP	PLA2G1B (3ELO)	-4.1
DEHP	SULT2A1 (1J99)	-6.2

### Molecular dynamics simulation and binding free energy calculation

**Fig 9:**
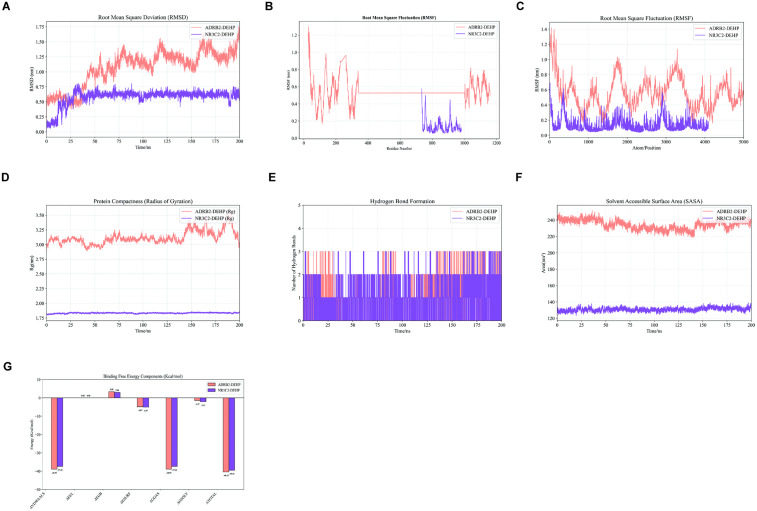
Structural dynamics and binding characteristics of ADRB2–DEHP and NR3C2–DEHP complexes during 200 ns molecular dynamics simulations. A Backbone RMSD. B-C. RMSF of residues and atoms. D. Rg E. Number of intermolecular hydrogen bonds. F. SASA. G. Binding free energy components.

In this study, 200 ns molecular dynamics simulations were performed to evaluate the structural stability, dynamic characteristics, and binding properties of two protein-ligand complexes, namely ADRB2-DEHP and NR3C2-DEHP. RMSD analysis revealed that the ADRB2-DEHP complex reached equilibrium after 50 ns of simulation, with backbone RMSD values stabilizing in the range of 1.0–1.5 nm during the equilibrium phase. In contrast, the backbone RMSD of the NR3C2-DEHP complex remained at a lower level of 0.25–0.75 nm throughout the simulation with smaller fluctuations, suggesting superior overall structural stability. RMSF results indicated that both residue and atomic flexibility were significantly higher in ADRB2-DEHP than in NR3C2-DEHP; the N-terminal region of ADRB2-DEHP exhibited high flexibility, whereas NR3C2-DEHP displayed stronger structural rigidity across the entire sequence. Rg analysis showed that the Rg values of ADRB2-DEHP and NR3C2-DEHP were stably maintained at 3.0–3.3 nm and 1.75–1.85 nm, respectively, with no obvious unfolding observed in either complex, indicating stable overall folding of the proteins. Hydrogen bond analysis demonstrated that the number of intermolecular hydrogen bonds was generally higher in the ADRB2-DEHP complex than in NR3C2-DEHP. SASA results revealed a significantly larger SASA in ADRB2-DEHP compared with NR3C2-DEHP, reflecting differences in solvent exposure between the two systems. Binding free energy decomposition analysis showed that the binding energies of both complexes were mainly driven by noncovalent interactions including van der Waals forces and electrostatic interactions, with distinct contribution patterns among individual energy components (Fig 9). Furthermore, to visually characterize ligand binding within the active site, snapshots extracted at 20 ns intervals during the simulation were superimposed in supplementary materials to verify the consistency and stability of the ligand-binding mode (S1 Fig).

## Discussion

GC remains one of the most prevalent and lethal malignancies worldwide. Changes in dietary habits, such as increased intake of processed foods and high-salt diets, along with modern lifestyle factors including irregular eating patterns and chronic psychological stress, have led to a rising incidence of gastritis and other gastrointestinal disorders, thereby further promoting the development of GC [10]. Notably, processed foods are often packaged in PVC materials containing plasticizers, which may serve as an underrecognized environmental risk factor for GC progression.

DEHP, a widely used high-molecular-weight plasticizer, is present in numerous everyday items such as food packaging, cosmetics, and medical equipment [8]. Human exposure occurs mainly through ingestion, inhalation, or dermal contact, with measurable metabolite levels detected in most general populations, raising consistent concerns about its chronic toxic effects [8]. In the present study, we performed integrative bioinformatic analyses to explore computationally predicted associations between DEHP and GC, rather than demonstrating direct causal effects. The gene expression signature was derived from tumor‑versus‑normal comparisons and reflects gastric cancer biology, not DEHP exposure biology. DEHP exposure damaged the intestinal barrier function and promoted intestinal inflammatory responses. Intestinal barrier damage and chronic inflammation were important pathological bases for GC occurrence, which indirectly supports a plausible biological basis for the potential association between DEHP exposure and GC [17]. Epidemiological evidence has linked environmental DEHP exposure to elevated cancer risk in multiple organs, including the breast, liver, and digestive system. Real-world data demonstrated that elevated baseline and annual average DEHP concentrations were associated with increased risks of benign, in situ, and malignant breast tumors in women; this association was not significantly modified by menopausal status or ethnicity but was more pronounced in younger women and oral contraceptive users, and remained consistent in sensitivity analyses excluding cases diagnosed within 2 years after baseline, supporting the need to regulate environmental DEHP emissions to reduce related breast tumor risks [18]. This cross-sectional study, based on a large sample (n = 6147) from the U.S. NHANES 2011–2018, confirmed that urinary concentrations of DEHP metabolites were significantly and positively associated with the overall prevalence of cancer, with adjusted OR values ranging from 1.17 to 1.29. These findings provide critical human population-based evidence supporting DEHP as a potential human carcinogen [19]. A meta-analysis including 11 studies (3,101 cancer cases and 6,858 controls) demonstrated that urinary levels of MEHHP, MECPP, DBP, and other metabolites were significantly associated with cancer risk [20]. Studies on the risk of DEHP-induced liver cancer in humans have shown that the risk of hepatoblastoma, a rare form of childhood liver cancer, is significantly increased in children with very low birth weight [21]. Several other studies have demonstrated that the duration of neonatal intensive care, which may involve extensive use of PVC-containing medical devices, is significantly associated with both the incidence [22] and severity [23] of hepatoblastoma. In gastric tissues, previous experimental studies suggested that DEHP may promote inflammatory responses, disrupt barrier function, and activate oncogenic signaling pathways even at low concentrations. Very low concentrations of DEHP promoted EMT in GC cells by activating the PI3K/AKT/mTOR and Smad2 signaling pathways, thereby significantly enhancing cell migration and invasion [10]. Previous studies have shown that even low concentrations of DEHP can enhance COX-2 expression in GC cells by activating ERK1/2 and NF-κB signaling [24]. Moreover, studies have found that DEHP can enhance the cytotoxicity of Helicobacter pylori and trigger apoptosis in gastric epithelial cells [9]. However, direct human evidence linking DEHP exposure to GC remains limited, and real-world exposure levels, dose-response relationships, and tissue-specific effects in the stomach are not fully established. In the present study, we performed integrative bioinformatic analyses to explore potential associations between DEHP and GC, rather than demonstrating direct causal effects.

Through an integrative analysis of compound- and disease-related targets, we identified 18 overlapping DEHP-GC targets, and machine learning further narrowed these to seven core genes (*ADRB2*, *ESRRG*, *GRIA4*, *IL13RA2*, *NR3C2*, *PLA2G1B*, and *SULT2A1*). Functional enrichment suggested that these genes are closely linked to lipid metabolic processes, extracellular region signaling, and estrogen response element binding, including metabolic reprogramming and altered signal transduction cascades. Given the well‑recognized molecular and histological heterogeneity of GC, the divergent predictive contributions of these genes across subgroups observed in SHAP analyses further support the notion that such targets may exert subtype‑specific effects rather than uniform functions. Although the present study did not directly infer cell‑type‑specific contributions, the enrichment of extracellular and membrane‑related processes suggests potential involvement of stromal components, epithelial cells, and signaling crosstalk between cell compartments in GC tissues.

Among the seven core genes, NR3C2 and ADRB2 showed relatively high predictive importance in our machine learning models. NR3C2, a nuclear transcription factor encoding the mineralocorticoid receptor (MR protein) [25], has been found to exhibit abnormal expression in GC [26]. For instance, overexpression of miR-21 in the general domain negatively regulated the expression of *CCL28*, *NR3C2*, and *SYNPO2*, thereby contributing to cancer progression [27]. Additionally, studies have demonstrated that NR3C2 can inhibit the proliferation and epithelial-mesenchymal transformation (EMT) of pancreatic cancer cells and enhance their sensitivity to certain chemotherapeutic drugs [28]. The *ADRB2* gene encodes the β2-adrenergic receptor, a member of the G protein-coupled receptor superfamily, and plays important roles in various cancers. It is involved in regulating GC progression [11]. Specifically, stress hormone-induced activation of the ADRB2 signaling pathway was critical for GC progression and metastasis [29]. Moreover, high ADRB2 protein levels were positively correlated with tumor-node-metastasis (TNM) staging and poor prognosis in GC patients [11]. In this study, to further validate the stable binding of DEHP to these core targets, particularly NR3C2 and ADRB2, we performed 200 ns all-atom molecular dynamics simulations. Molecular dynamics simulations confirmed stable binding of DEHP to ADRB2 and NR3C2, supporting structural plausibility. This did not confirm that DEHP exposure caused the observed gene expression changes in GC, as the DEG signature reflects GC biology rather than DEHP exposure biology, their direct functional roles in mediating potential DEHP‑associated gastric carcinogenesis remain to be experimentally validated.

*ESRRG*, an estrogen‑related receptor, has been shown to suppress gastric carcinogenesis via inhibition of Wnt signaling pathway [30]. *GRIA4* promoter hypermethylation has been proposed as an early diagnostic event in GC [31,32], consistent with its downregulation in our analysis. *IL13RA2*, a high-affinity membrane receptor for IL-13, is highly expressed in various tumors. It has been reported to accelerate the progression of infantile hemangioma by activating glycolysis and the Wnt/β-catenin signaling pathway [33]. Moreover, IL13RA2 was also highly expressed in several glioma subtypes [34]. *IL13RA2* promoted the proliferation and growth of brain metastases from breast cancer [35]. Phospholipase A2 (PLA2), a large enzyme family involved in inflammatory processes, catalyzes the hydrolysis of membrane phospholipids to produce free fatty acids and lysophospholipids, thereby initiating the arachidonic acid cascade [36]. The expression of PLA2 family members is related to the behavior of several cancer types [36]. Studies have shown that *PLA2G1B* gene polymorphism affects susceptibility to rectal cancer [37]. *SULT2A1*, a sulfotransferase involved in steroid and bile acid metabolism, was significantly downregulated in GC tissues in our analysis [38]. Compared with normal gastric mucosa tissues, *SULT2A1* expression was significantly decreased in GC tissues [39]. Although most functional evidence for SULT2A1 came from HCC studies [40,41], its role in bile acid homeostasis and lipid metabolism suggested potential relevance to gastric carcinogenesis, as dysregulated bile acid signaling and metabolic disruption were well‑established contributors to gastric inflammation and malignant transformation. Thus, downregulation of SULT2A1 in GC might reflect metabolic reprogramming in gastric epithelial cells, which warranted further investigation in the context of environmental exposure such as DEHP.

Collectively, these genes and their associated pathways suggested that DEHP-related GC might involve a complex network of metabolic dysregulation, hormone receptor signaling, inflammatory cascades, and subtype‑dependent transcriptional programs, rather than linear gene‑level effects.

Our study has several limitations that should be acknowledged.

First, all findings were derived from computational and predictive analyses, including target prediction, WGCNA, machine learning, and molecular docking. To provide further support for our computational predictions, we further performed MD simulations to validate DEHP binding stability to NR3C2 and ADRB2. No causal or functional evidence was directly demonstrated, and conclusions remain hypothetical until validated by *in vitro* and *in vivo* experiments. Second, GC is highly heterogeneous at molecular, histological, and clinical levels, and the present study did not fully stratify samples by molecular subtype, Lauren classification, H. pylori status, or clinical stage. Cell‑type composition and spatial heterogeneity within gastric tissues were not directly evaluated, which may influence interpretability. Third, real‑world DEHP exposure levels, duration, dose-response relationships, and co‑exposure to other environmental toxicants were not integrated. direct transcriptomic data from DEHP‑exposed gastric cells or animal models were unavailable in public datasets, and we were unable to identify relevant GEO datasets involving DEHP exposure; this limitation restricts the strength of causal inference between DEHP exposure and transcriptional alterations in GC. Fourth, although multiple machine learning models were used to enhance robustness, the feature set was restricted to 18 preselected genes, and validation in larger independent cohorts is needed to improve apparent performance in this study. Meanwhile, given the relatively limited feature space, we paid close attention to the potential risk of overfitting and <font color = “red”>employed k-fold cross-validation during the training phase for hyperparameter optimization in core algorithms (such as Elastic Net, XGBoost, and GBM) to mitigate this risk. Fifth, <font color=”red”>although we adopted cross-validation for hyperparameter tuning within single model training and separated the training and validation processes, the optimal model was selected from 127 candidate models based on predictive performance on the validation cohort. As the reviewer correctly pointed out, selecting the top model from a large candidate pool using validation performance is itself a form of optimistic selection. The validation cohort has effectively been used for model selection rather than as a purely held-out evaluation set. Therefore, the reported AUC is likely upwardly biased and should be regarded as a promising estimate rather than an absolute robust performance indicator. Claims about model “reliability” should be interpreted cautiously. Finally, the mechanistic interplay between the identified pathways, their regulation by DEHP, and tissue‑specific effects in the stomach remain incompletely understood. Future studies should combine *in vitro* and *in vivo* models of DEHP exposure, transcriptomic profiling, and functional validation to clarify the biological plausibility and causality of these targets and pathways in gastric carcinogenesis.

Future studies should conduct validation using in vitro cell models and in vivo animal models with exposure to DEHP and its active metabolite MEHP. *In vitro,* gastric epithelial cells and GC cell lines should be treated with environmentally relevant concentrations of DEHP/MEHP to detect the expression of NR3C2 and ADRB2, as well as changes in downstream signaling pathways, accompanied by functional assays including proliferation, apoptosis, migration, and invasion. *In vivo*, a chronic DEHP exposure model should be established to observe gastric mucosal injury and progression of precancerous lesions. Furthermore, functional rescue and intervention experiments should be performed to clarify the biological plausibility and causal relationship of the DEHP‑NR3C2/ADRB2 axis in GC, thus achieving a smooth transition from computational predictions to experimental validation.

## Materials and methods

### Acquisition of compound-related targets

The structural data of DEHP were obtained from the PubChem database (https://pubchem.ncbi.nlm.nih.gov/) [42]. Potential target proteins associated with DEHP were collected from four publicly available databases: ChEMBL (https://www.ebi.ac.uk/chembl/) [43], PharmMapper (https://lilab-ecust.cn/pharmmapper/index.html) [44] and SwissTargetPrediction (http://swisstargetprediction.ch/) [45] databases. The overall workflow employed in this study is schematically illustrated in Fig 1 [46].

### Data collection of gastric cancer

Whole-transcriptome sequencing (WTS) has become a powerful and widely used tool for cancer transcriptomic research due to its superior resolution in detecting novel transcripts, alternative splicing events and low-abundance RNAs, and we fully acknowledge its technical advantages in comprehensive transcriptome profiling. However, microarray datasets were selected for the present study to investigate the molecular mechanisms of DEHP-related GC based on three critical and study-specific considerations: first, the GEO database provides a large number of well-annotated GC microarray datasets with large sample sizes and paired GC/adjacent non-tumor tissues, which is essential for identifying robust differentially expressed genes (DEGs) and conducting performance co-expression network analysis. Second, these microarray datasets have been extensively used and validated in previous GC and environmental toxicant-related transcriptomic studies, ensuring the comparability of our results with existing literature on DEHP-related gene expression changes. Third, the selected microarray datasets are mostly based on consistent Affymetrix platforms, which facilitates efficient batch effect correction and data integration, thus reducing technical variation in cross-dataset analysis. All included microarray datasets met strict inclusion criteria: containing paired GC and adjacent non-tumor tissues, with complete sample annotation, and no obvious technical bias in raw data. Six microarray datasets related to GC, specifically GSE66229, GSE65801, GSE54129, GSE51575, GSE19826, and GSE13911, were retrieved from the GEO (https://www.ncbi.nlm.nih.gov/geo/) [47] database. The GSE66229 dataset comprises 400 samples, including 300 GC tissues and 100 adjacent non-tumor tissues, which were detected using the Affymetrix GPL570 platform. GSE65801 contains 64 samples, consisting of 32 GC tissues and 32 adjacent non-tumor tissues, detected using the Affymetrix GPL14550 platform. GSE54129 includes 132 samples, consisting of 111 GC tissues and 21 adjacent non-tumor tissues, analyzed on the Affymetrix GPL570 platform. GSE51575 consists of 52 samples, involving 26 GC tissues and 26 adjacent non-tumor tissues, detected based on the Affymetrix GPL13607 platform. GSE19826 contains 27 samples, with 12 GC tissues and 15 adjacent non-tumor tissues, analyzed using the Affymetrix GPL570 platform. GSE13911 comprises 69 samples, including 38 GC tissues and 31 adjacent non-tumor tissues, detected on the Affymetrix GPL570 platform. Among these datasets, GSE66229, GSE65801, and GSE54129 were designated as the training cohort, while GSE51575, GSE19826, and GSE13911 served as the validation cohort.

### GEO data preprocessing

Probe-to-gene mapping: Probe-level data were converted to gene-level using platform-specific GPL annotation files (GEO): Valid annotation rows were screened (row 11 for GPL570, row 7 for GPL13607, row 13 for GPL14550); empty rows/rows starting with “ID”/”!” were excluded. Gene symbols were cleaned (truncated at “///”, quotation marks removed, multi-gene annotations with spaces filtered out). For genes mapped to multiple probes, average expression was calculated; unmapped probes were excluded. Mapping results were saved for traceability. Normalization and batch correction: Raw expression values (≤0 set to 0) were log2-transformed. Cross-platform normalization was done via limma::normalizeBetweenArrays (quantile method). Batch effects (batch/platform differences) were corrected using SVA (surrogate variable estimation via num.sv/sva functions) and ComBat (par.prior = TRUE, mean.only = FALSE). PCA validated batch correction efficacy (PCA plots/scores saved); corrected data were saved as batch_corrected_data.csv. Dataset integration: Only common genes across all datasets were retained for integrative analysis to ensure consistency between training/validation cohorts.

### Analysis of gene differential expression

To analyze the gene expression profile data and identify DEGs, the limma package was employed. DEGs were identified using a screening threshold of adjusted *P*-value < 0.05 and |log_2_ fold change| > 0.585 (corresponding to a 1.5-fold change in expression level). The false discovery rate (FDR) method was used for multiple testing correction to calculate adjusted p-values. All genes in the batch-corrected expression matrix (without prior filtering) were included in the analysis, and the total number of genes before filtering was consistent with the number of common genes screened from the six GEO datasets. For visualizing these DEGs, the pheatmap package was used to generate heatmaps, while volcano plots were constructed using the ggplot2 package.

### Weighted gene co-expression network analysis and acquisition of key targets

Weighted gene co-expression network analysis (WGCNA) was performed using the R package WGCNA to identify key modules associated with GC. Initially, the pickSoftThreshold function was utilized to compute the optimal soft-thresholding parameter (ranging from 1 to 20), ensuring the network conformed to the scale-free topological property (scale-free R^2^ > 0.80). Prior to network construction, low-quality genes and outlier samples were filtered: genes with a standard deviation < 0.5 across samples were excluded, and sample outliers were identified via hierarchical clustering (cut height = 20000) and removed to ensure data robustness.

Following quality control, the adjacency matrix was constructed using the optimal soft-thresholding power, then transformed into a topological overlap matrix (TOM) to minimize the impact of noise and spurious correlations. The dissimilarity of TOM (1-TOM) was calculated and used for hierarchical clustering of genes. Gene modules were identified via the dynamic tree-cutting algorithm with the following parameters: minimum module size = 50, deepSplit = 2, and pamRespectsDendro = FALSE. Modules with high similarity (module eigengene dissimilarity < 0.25) were merged to reduce redundancy.

To prioritize biologically relevant modules, module-trait association analysis was conducted by calculating the Pearson correlation between module eigengenes and clinical traits (normal vs. disease status), with corresponding p-values determined via student’s t-test. For genes within trait-associated modules, module membership (MM) and gene significance (GS) were quantified:

MM was defined as the Pearson correlation between individual gene expression and the module eigengene (threshold: |MM| ≥ 0.8), reflecting the degree of gene connectivity within the module;

GS was defined as the Pearson correlation between individual gene expression and disease status (threshold: |GS| ≥ 0.2), reflecting the biological relevance of genes to the phenotype.

Genes meeting both MM (|MM| ≥ 0.8) and GS (|GS| ≥ 0.2) thresholds were designated as key module genes. Finally, to identify GC key targets, intersection analysis was performed between DEGs and key module genes derived from WGCNA. The overlap of gene sets was visualized using Venn diagrams and ComplexUpset plots, and the intersecting genes were extracted as core targets for subsequent analysis.

### Di(2-ethylhexyl) phthalate-related gastric cancer-related targets

An intersection analysis was conducted between DEHP-related targets and the key targets of GC, and the results were visualized by a Venn diagram.

### Protein-protein interaction network analysis

The intersection targets were imported into the STRING database (version 12.0, https://string-db.org/) [48] with the species restricted to Homo sapiens. All interaction evidence types (coexpression, experiment, database, textmining, and homology) were retained, and a functional association network was constructed with a combined score ≥ 0.15. The network was visualized using Cytoscape 3.8.2 [49].

### GO and KEGG analyses

An intersection analysis was performed between DEHP-related targets and the key targets of GC, and the results were visualized by a Venn diagram. The overlapping targets were uploaded to The Database for Annotation, Visualization and Integrated Discovery (DAVID, https://david.ncifcrf.gov/) [50] to perform Gene Ontology (GO) enrichment analysis, and the results were visualized using the online platform (http://www.bioinformatics.com.cn/) [51,52]. Subsequently, Kyoto Encyclopedia of Genes and Genomes (KEGG) pathway analysis was conducted using the clusterProfiler package in R software. Gene symbols were mapped to Entrez IDs via org.Hs.egSYMBOL2EG; initial enrichment used *P*-value < 0.5, and significant pathways were filtered by *P*-value < 0.05 and q-value (Benjamini-Hochberg adjustment). Bubble charts were used to present the top 15 GO terms, covering biological process (BP), cellular component (CC), and molecular function (MF), whereas bar charts were employed to visualize the results of the top 15 pathway analyses.

### Machine learning and model construction

To further identify the key targets involved in DEHP‑related GC, an integrated machine learning‑based prediction framework was established using multiple algorithms. A total of 127 machine learning models were constructed in R software, including Elastic Net, Random Forest, XGBoost, and other classical approaches, to screen core features and systematically evaluate model performance. All 18 candidate genes were used as the initial input features, and no additional feature selection procedure was performed prior to model training. To ensure data comparability and improve model convergence, feature scaling and standardization were applied to both the training and validation cohorts before model construction, which is particularly critical for regularized regression methods such as Elastic Net and machine learning algorithms sensitive to feature scales. To enhance reproducibility, a fixed random seed (123) was set throughout the analysis.

Regarding hyperparameter optimization and cross-validation: To mitigate overfitting risk, a k‑fold cross‑validation strategy was adopted during the model training phase for hyperparameter optimization in core algorithms. Specifically, Elastic Net, Lasso, and Ridge employed 10-fold CV (nfolds = 10) to determine the optimal lambda; GBM utilized 10-fold CV (cv.folds = 10) to select the best iteration; XGBoost applied 5-fold CV to identify the optimal number of rounds; glmBoost used k-fold CV to determine the optimal mstop; and LDA used CV within the caret framework. For algorithms like SVM, Naive Bayes, and Stepglm, which relied on default settings or AIC/BIC criteria for variable selection in the current framework, explicit cross-validation tuning was not applied; Random Forest utilized out-of-bag (OOB) error for internal evaluation. Since the 18 candidate genes were predefined by prior bioinformatic analyses, no feature selection was conducted within the cross-validation loop. The parameter min.selected.var = 2 was used to exclude overly simplistic models with ≤2 features.

Model selection strategy: < font color = “red” > The optimal glmBoost+Enet model was selected from 127 candidates based on its AUC performance in the validation cohort. It should be clearly noted that using the validation set to select the best model from a large candidate pool means the validation cohort was effectively used for model selection rather than as a purely held-out evaluation set. This approach may introduce mild optimistic bias and lead to upwardly biased AUC estimates. We did not perform nested cross-validation or reserve an additional fully independent GEO dataset for out-of-sample testing. The 127 models represented comprehensive combinations of distinct machine learning algorithms and parameter settings. The pROC package was used to calculate the AUC, and models with AUC > 0.9 were defined as high‑performance models. Core genes were identified according to their occurrence frequency in these high‑performance models. Furthermore, the SHAP package was applied to compute SHAP values based on the optimal machine learning model and the full dataset. Dependence plots and force plots were generated to quantify each gene’s predictive contribution, reveal the relationship between gene expression levels and SHAP values, and visualize the directional effects of individual genes in sample‑specific predictions.

### Analysis of abnormal gene expression

The University of ALabama at Birmingham CANcer data analysis Portal (UALCAN, https://ualcan.path.uab.edu/) [53] was utilized to explore the abnormal expression patterns of the 7 core targets. The analysis was based on the TCGA GC dataset, which included 449 samples in total: 415 GC tissues and 34 normal tissues. Unpaired two‑sample t‑test was employed to evaluate the significance of expression differences between cancer and normal groups, with *P* < 0.05 considered statistically significant.

### Molecular docking

To further evaluate the binding affinity between the active components of the compound and the corresponding targets, molecular docking validation was performed. In this study, an automated molecular docking strategy based on a semi-empirical scoring function was employed to investigate the interaction mechanism and binding affinity between small-molecule ligands and target protein receptors. The 2D structures of small-molecule ligands were downloaded from the PubChem database (https://pubchem.ncbi.nlm.nih.gov/) [54], and the crystal structures of target proteins were obtained from the Protein Data Bank (PDB, http://www.rcsb.org/pdb/) [55]. First, the target protein crystal structures were preprocessed using the PyMOL molecular graphics system to remove crystal water molecules, auxiliary ions and irrelevant ligands, and protonation state optimization was completed by adding polar hydrogen atoms and assigning Gasteiger charges. For ligand molecules, the Open Babel toolkit was used for 3D conformation construction, energy minimization and torsion bond identification to ensure the stereochemical rationality of the input structures. Subsequently, the docking search space was defined. A grid box sufficiently covering the potential binding region was set centered on the known active site or cofactor-binding pocket, with a grid spacing of 0.375 Å to ensure the accuracy and efficiency of conformational sampling. Docking calculations were strictly performed on the AutoDock Vina software platform, which adopts an improved gradient-optimized global search strategy that effectively balances the depth of conformational space exploration and computational cost. To ensure the consistency of the results, the mode with the lowest binding energy and the highest conformational clustering compactness (Mode 1) was selected as the optimal binding conformation for subsequent analysis. To further clarify the structural basis of molecular recognition, the optimal docking complex was imported into PyMOL for high-precision 3D visualization, which intuitively presented the orientation of the ligand in the binding pocket and its spatial matching with key catalytic residues.

### Molecular dynamics simulation and binding free energy calculation

To assess the dynamic stability of the screened compounds within the protein binding cavity and further support the molecular docking results, 200 ns all-atom molecular dynamics simulations were performed using the GROMACS 2023.2 software package with the CHARMM36 force field and TIP3P water model. The initial protein–ligand complex structures were derived from docking conformations. Each system was solvated in a cubic box with a 1.0 nm buffer distance and neutralized with Na^+^ /Cl^‒^ ions to achieve a physiological ionic strength of 0.15 mol·L^‒1^. Energy minimization was conducted using the steepest descent algorithm until the maximum force was below 1000 kJ·mol^‒1^·nm^‒1^. Subsequently, 100 ps NVT equilibration at 300 K (V-rescale thermostat, τ_T = 0.1 ps) and 100 ps NPT equilibration at 1 bar (Parrinello–Rahman barostat, τ_P = 2.0 ps) were performed with position restraints on the protein backbone.

Unrestrained 200 ns production MD simulations were then carried out with a 2 fs time step. Bond lengths were constrained using the LINCS algorithm. A cutoff of 1.2 nm was applied for nonbonded interactions, and long-range electrostatic interactions were calculated using the Particle-Mesh Ewald (PME) method. Trajectory frames were saved every 10 ps for subsequent analysis.

Structural stability was evaluated by root-mean-square deviation (RMSD), root-mean-square fluctuation (RMSF), radius of gyration (Rg), solvent-accessible surface area (SASA), and hydrogen bond occupancy. To visually confirm the consistency of ligand binding, structural snapshots were extracted at 20 ns intervals throughout the 200 ns simulation and superimposed using PyMOL 2.5. Principal component analysis (PCA) was performed to characterize global conformational motions and construct two-dimensional free energy surfaces (FES).

To further quantify binding affinity in a rigorous manner, molecular mechanics Poisson–Boltzmann surface area (MM-PBSA) analysis was performed using the g_mmpbsa module implemented in GROMACS. The binding free energy (ΔG_total) and its energetic components, including van der Waals (ΔE_vdW), electrostatic (ΔE_ele), polar solvation (ΔE_GB), and nonpolar solvation (ΔE_surf) contributions, were calculated to validate the stability and energetic favorability of protein–ligand binding. All MD trajectories were visualized using VMD 1.9.4 and PyMOL 2.5.

### Statistical analysis

All data analyses were performed using R software (version 4.5.0).

## Supporting information

S1 Fig Superimposed snapshots of ADRB2–DEHP and N3C2–DEHP complexes extracted at 20 ns intervals during 200 ns MD simulations. (TIF)

S1 Table List of top DEGs. (DOCX)

S2 Table Performance of the 127 constructed prediction models. (DOCX)
