# Correction: Screening of core targets for Di(2-ethylhexyl) Phthalate-related gastric cancer based on machine learning, molecular docking, and SHAP analysis

**DOI:** 10.1371/journal.pcbi.1014783

**Published:** 2026-09-14

**Authors:** Shenghao Li, Qing Peng, Liyuan Hao, Jingyu Mao, Bingjie Huo

In the Results subsection of the Abstract, there is an error in the third sentence of the paragraph. The correct sentence is: Through machine learning analyses, 7 key genes (ADRB2, ESRRG, GRIA4, IL13RA2, NR3C2, PLA2G1B, and SULT2A1) were identified as core targets in GC.

In the Discussion section, there is an error in the second paragraph. The full redundant duplicate last sentence should be remove.

In the Discussion section, there is an error in the last sentence of the fourth paragraph. The correct sentence is: This did not confirm that DEHP exposure caused the observed gene expression changes in GC, as the DEG signature reflects GC biology rather than DEHP exposure biology. Their direct functional roles in mediating potential DEHP associated gastric carcinogenesis remain to be experimentally validated.

In the Discussion section, there is an error in the first sentence of the fifth paragraph. The correct sentence is: ESRRG, an estrogen related receptor, has been shown to suppress gastric carcinogenesis via inhibition of the Wnt signaling pathway.

In the Discussion section, there is an error in the last sentence of the fifth paragraph. The correct sentence is: Thus, downregulation of SULT2A1 in GC might reflect metabolic reprogramming in gastric epithelial cells, which warrants further investigation in the context of environmental exposure such as DEHP.

In the Discussion section, there are errors in the eight paragraph. The correct paragraph is: First, all findings were derived from computational and predictive analyses, including target prediction, WGCNA, machine learning, and molecular docking. To provide further support for our computational predictions, we further performed MD simulations to validate DEHP binding stability to NR3C2 and ADRB2. No causal or functional evidence was directly demonstrated, and conclusions remain hypothetical until validated by in vitro and in vivo experiments. Second, GC is highly heterogeneous at molecular, histological, and clinical levels, and the present study did not fully stratify samples by molecular subtype, Lauren classification, H. pylori status, or clinical stage. Cell‑type composition and spatial heterogeneity within gastric tissues were not directly evaluated, which may influence interpretability. Third, real‑world DEHP exposure levels, duration, dose-response relationships, and co‑exposure to other environmental toxicants were not integrated. direct transcriptomic data from DEHP‑exposed gastric cells or animal models were unavailable in public datasets, and we were unable to identify relevant GEO datasets involving DEHP exposure; this limitation restricts the strength of causal inference between DEHP exposure and transcriptional alterations in GC. Fourth, although multiple machine learning models were used to enhance robustness, the feature set was restricted to 18 preselected genes, and validation in larger independent cohorts is needed to improve apparent performance in this study. Meanwhile, given the relatively limited feature space, we paid close attention to the potential risk of overfitting and employed k-fold cross-validation during the training phase for hyperparameter optimization in core algorithms (such as Elastic Net, XGBoost, and GBM) to mitigate this risk. Fifth, although we adopted cross-validation for hyperparameter tuning within single model training and separated the training and validation processes, the optimal model was selected from 127 candidate models based on predictive performance on the validation cohort. Selecting the top model from a large candidate pool using validation performance is itself a form of optimistic selection. The validation cohort has effectively been used for model selection rather than as a purely held-out evaluation set. Therefore, the reported AUC is likely upwardly biased and should be regarded as a promising estimate rather than an absolute robust performance indicator. Claims about model “reliability” should be interpreted cautiously. Finally, the mechanistic interplay between the identified pathways, their regulation by DEHP, and tissue‑specific effects in the stomach remain incompletely understood. Future studies should combine in vitro and in vivo models of DEHP exposure, transcriptomic profiling, and functional validation to clarify the biological plausibility and causality of these targets and pathways in gastric carcinogenesis.

In the Acquisition of targets with significant disease-related changes subsection of the Results, there is an error in the last paragraph. The correct paragraph is: Hub genes in the MEgreen module were screened using the criteria |MM| ≥ 0.8 and |GS| ≥ 0.2. By intersecting these genes with the 863 DEGs, 122 overlapping genes were identified, which were regarded as candidate core dysregulated genes in GC (Fig 4E), suggesting that these genes are core co expressed DEGs and may play key regulatory roles in the induction and progression of GC.

In the Machine learning and model construction subsection of the Materials and methods, there is an error in the first sentence of the third paragraph. The correct sentence is: Model selection strategy: The optimal glmBoost+Enet model was selected from 127 candidates based on its AUC performance in the validation cohort.

The Data Availability statement for this article is incorrect. The correct statement is: All data generated or analyzed during this study are included in the published article and/or its supporting information files. The gastric cancer related gene expression data used in this study are available from the Gene Expression Omnibus (GEO) database (https://www.ncbi.nlm.nih.gov/geo/), with the accession numbers GSE66229, GSE65801, GSE54129, GSE51575, GSE19826 and GSE13911. The structural information of Di(2 ethylhexyl) phthalate (DEHP) is available from the PubChem database (PubChem CID: 8343, https://pubchem.ncbi.nlm.nih.gov/). R analysis scripts containing detailed parameter settings, the table result files S1 S19, molecular docking files and molecular dynamics simulation parameter documents are archived in a dedicated protocol hosted on protocols.io (DOI: https://dx.doi.org/10.17504/protocols.io.81wgbmdmyvpk/v1).
