# Supplementary material for: Screening of core targets for Di(2-ethylhexyl) Phthalate-related gastric cancer based on machine learning, molecular docking, and SHAP analysis
Source: PLoS Comput Biol. 2026 Jul 16;22(7):e1014514. doi: 10.1371/journal.pcbi.1014514 (PMC13374966; doi:10.1371/journal.pcbi.1014514)
Supplement: S1 Table — (DOCX) [file pcbi.1014514.s002.docx]

**S1 Table List of top DEGs**

| Gene | log_2_ fold change | SE | AveExpr | t | P.Value | adj.P.Val | B |
| --- | --- | --- | --- | --- | --- | --- | --- |
| KCNMB2 | -1.05376 | 0.041745 | 2.067756 | -25.2431 | 3.24E-96 | 5.62E-92 | 208.6313 |
| MAL | -1.80812 | 0.072197 | 3.83459 | -25.0444 | 3.68E-95 | 3.19E-91 | 206.2178 |
| ARHGEF37 | -0.79578 | 0.033873 | 3.633212 | -23.4932 | 6.42E-87 | 3.71E-83 | 187.3675 |
| GRIA3 | -0.6022 | 0.025865 | 1.778655 | -23.2828 | 8.40E-86 | 3.64E-82 | 184.8121 |
| INHBA | 1.771569 | 0.076225 | 3.451773 | 23.24139 | 1.39E-85 | 4.84E-82 | 184.3087 |
| TEAD4 | 0.732005 | 0.032122 | 3.528566 | 22.78795 | 3.55E-83 | 1.03E-79 | 178.8052 |
| PLCXD3 | -1.35796 | 0.062879 | 2.36903 | -21.5964 | 7.15E-77 | 1.55E-73 | 164.3825 |
| SERTM1 | -0.63819 | 0.029654 | 1.405648 | -21.5212 | 1.78E-76 | 3.43E-73 | 163.4758 |
| C16orf89 | -1.67766 | 0.079004 | 3.983047 | -21.2353 | 5.72E-75 | 9.93E-72 | 160.0281 |
| CLDN1 | 1.625588 | 0.077031 | 3.314066 | 21.10312 | 2.84E-74 | 4.48E-71 | 158.4368 |
| VSTM2A | -0.78236 | 0.037088 | 1.788953 | -21.0944 | 3.16E-74 | 4.56E-71 | 158.3316 |
| PAIP2B | -1.02613 | 0.049648 | 3.115255 | -20.6681 | 5.47E-72 | 6.78E-69 | 153.2093 |
| ADRB2 | -0.96615 | 0.047204 | 3.63115 | -20.4673 | 6.17E-71 | 6.82E-68 | 150.8015 |
| UBE2QL1 | -1.07543 | 0.052548 | 2.238473 | -20.4657 | 6.29E-71 | 6.82E-68 | 150.7832 |
| ADHFE1 | -0.88357 | 0.043449 | 3.433614 | -20.3361 | 3.00E-70 | 2.89E-67 | 149.231 |
| ESRRG | -1.72557 | 0.085283 | 3.022507 | -20.2335 | 1.03E-69 | 9.41E-67 | 148.0042 |
| CKMT2 | -1.509 | 0.074722 | 3.081188 | -20.1947 | 1.64E-69 | 1.43E-66 | 147.5404 |
| MAMDC2 | -1.71375 | 0.086476 | 3.143556 | -19.8176 | 1.52E-67 | 1.15E-64 | 143.0423 |
| PLAU | 1.009552 | 0.051646 | 3.940891 | 19.54744 | 3.85E-66 | 2.79E-63 | 139.8301 |
| ATP4A | -3.63846 | 0.186216 | 3.520331 | -19.5389 | 4.27E-66 | 2.96E-63 | 139.7289 |
| ASPA | -0.87115 | 0.044635 | 2.819682 | -19.5174 | 5.52E-66 | 3.68E-63 | 139.4729 |
| SLC26A7 | -1.10763 | 0.057442 | 1.728605 | -19.2826 | 9.08E-65 | 5.25E-62 | 136.6901 |
| ALDH6A1 | -0.63238 | 0.032991 | 3.714485 | -19.1681 | 3.55E-64 | 1.92E-61 | 135.3366 |
| TMEM158 | 1.072745 | 0.056189 | 3.877937 | 19.09179 | 8.79E-64 | 4.62E-61 | 134.4345 |
| SIDT2 | -0.62068 | 0.032677 | 4.787074 | -18.9944 | 2.80E-63 | 1.39E-60 | 133.2848 |
| DPT | -1.52353 | 0.080374 | 4.133811 | -18.9555 | 4.44E-63 | 2.14E-60 | 132.8266 |
| LIFR | -0.9832 | 0.051924 | 3.093465 | -18.9352 | 5.64E-63 | 2.65E-60 | 132.5875 |
| PLA2G7 | 1.365041 | 0.072177 | 3.8787 | 18.91244 | 7.40E-63 | 3.38E-60 | 132.3188 |
| RPS6KA6 | -0.59462 | 0.031822 | 1.79251 | -18.6862 | 1.08E-61 | 4.56E-59 | 129.6573 |
| LRP8 | 1.013414 | 0.054442 | 3.025285 | 18.61453 | 2.51E-61 | 1.04E-58 | 128.8158 |
| MYZAP | -0.93687 | 0.050462 | 3.708751 | -18.5659 | 4.46E-61 | 1.76E-58 | 128.2458 |
| ATP4B | -2.95002 | 0.159378 | 3.133208 | -18.5096 | 8.67E-61 | 3.34E-58 | 127.5852 |
| SERPINH1 | 0.896251 | 0.048473 | 5.053666 | 18.48988 | 1.09E-60 | 4.13E-58 | 127.3543 |
| CDH3 | 1.147358 | 0.062096 | 3.609311 | 18.47723 | 1.27E-60 | 4.69E-58 | 127.2061 |
| PLEKHG1 | 0.64621 | 0.035067 | 3.496719 | 18.4276 | 2.28E-60 | 8.24E-58 | 126.6251 |
| DNER | -1.65683 | 0.090149 | 2.300608 | -18.3787 | 4.05E-60 | 1.43E-57 | 126.0535 |
| FBP2 | -2.06488 | 0.112418 | 2.897507 | -18.3679 | 4.60E-60 | 1.60E-57 | 125.9264 |
| FJX1 | 0.815508 | 0.04441 | 3.315455 | 18.3633 | 4.86E-60 | 1.65E-57 | 125.8729 |
| GPR155 | -0.96941 | 0.052891 | 3.965356 | -18.3286 | 7.31E-60 | 2.35E-57 | 125.467 |
| LRRC3B | -0.78276 | 0.043162 | 1.662445 | -18.1356 | 7.06E-59 | 2.15E-56 | 123.2146 |
| ADH7 | -1.42423 | 0.078558 | 2.028161 | -18.1298 | 7.55E-59 | 2.26E-56 | 123.1473 |
| APOBEC2 | -1.50384 | 0.083005 | 2.996802 | -18.1175 | 8.72E-59 | 2.52E-56 | 123.0041 |
| CTHRC1 | 1.491769 | 0.082486 | 4.762959 | 18.08518 | 1.27E-58 | 3.62E-56 | 122.6279 |
| SH3GL2 | -1.47914 | 0.081991 | 1.871427 | -18.0403 | 2.15E-58 | 6.03E-56 | 122.106 |
| PMEPA1 | 0.861145 | 0.047957 | 4.451076 | 17.9566 | 5.74E-58 | 1.56E-55 | 121.1325 |
| LIPG | 1.213018 | 0.067606 | 3.151505 | 17.94233 | 6.78E-58 | 1.79E-55 | 120.9667 |
| KAT2B | -0.62641 | 0.034913 | 4.2563 | -17.9421 | 6.80E-58 | 1.79E-55 | 120.9642 |
| GRIA4 | -0.79575 | 0.044554 | 1.739307 | -17.8604 | 1.77E-57 | 4.38E-55 | 120.0155 |
| ERO1LB | -0.88276 | 0.049456 | 3.09687 | -17.8495 | 2.01E-57 | 4.90E-55 | 119.8893 |
| SDS | 0.758037 | 0.042529 | 2.958707 | 17.82416 | 2.70E-57 | 6.50E-55 | 119.5955 |
| CPA2 | -2.06527 | 0.116493 | 3.056893 | -17.7287 | 8.21E-57 | 1.95E-54 | 118.4894 |
| LY6E | 1.004714 | 0.056722 | 4.25272 | 17.71284 | 9.87E-57 | 2.28E-54 | 118.3063 |
| ENC1 | 0.861982 | 0.048914 | 4.257714 | 17.62233 | 2.83E-56 | 6.29E-54 | 117.2599 |
| LIF | 0.892326 | 0.050658 | 3.289578 | 17.6147 | 3.09E-56 | 6.79E-54 | 117.1717 |
| TNFRSF12A | 0.781372 | 0.044501 | 4.726395 | 17.55846 | 5.94E-56 | 1.27E-53 | 116.5225 |
| GRIN2D | 1.072755 | 0.061103 | 2.371486 | 17.5566 | 6.07E-56 | 1.27E-53 | 116.5011 |
| CHI3L1 | 1.638649 | 0.093365 | 3.281365 | 17.55093 | 6.49E-56 | 1.34E-53 | 116.4356 |
| ASXL3 | -0.69387 | 0.039664 | 2.374958 | -17.4934 | 1.26E-55 | 2.58E-53 | 115.7725 |
| CWH43 | -1.81459 | 0.103825 | 2.09966 | -17.4774 | 1.52E-55 | 3.07E-53 | 115.5884 |
| RNASE1 | -0.69829 | 0.039972 | 4.381474 | -17.4694 | 1.67E-55 | 3.33E-53 | 115.4957 |
| ENPP6 | -0.80923 | 0.046347 | 1.680208 | -17.4602 | 1.86E-55 | 3.67E-53 | 115.3895 |
| UBL3 | -0.61973 | 0.035507 | 5.071092 | -17.4538 | 2.00E-55 | 3.90E-53 | 115.3157 |
| SIGLEC11 | -1.15222 | 0.066143 | 2.237689 | -17.4202 | 2.96E-55 | 5.64E-53 | 114.9289 |
| TRIM50 | -1.33913 | 0.076877 | 2.483718 | -17.419 | 3.00E-55 | 5.65E-53 | 114.9155 |
| PPP1R3C | -1.02156 | 0.058747 | 3.314103 | -17.3892 | 4.23E-55 | 7.65E-53 | 114.5726 |
| DGKD | -0.76926 | 0.044401 | 4.738934 | -17.3252 | 8.88E-55 | 1.57E-52 | 113.8364 |
| ZNF662 | -0.88754 | 0.051284 | 2.908717 | -17.3063 | 1.10E-54 | 1.94E-52 | 113.6197 |
| ANGPT2 | 0.775121 | 0.044813 | 2.943659 | 17.29675 | 1.23E-54 | 2.14E-52 | 113.5098 |
| GPD1L | -0.64263 | 0.037184 | 4.840583 | -17.2823 | 1.46E-54 | 2.50E-52 | 113.3438 |
| SPINK2 | -1.20432 | 0.069728 | 2.605 | -17.2718 | 1.65E-54 | 2.80E-52 | 113.223 |
| ACADL | -0.93479 | 0.054235 | 1.933226 | -17.236 | 2.49E-54 | 4.15E-52 | 112.8126 |
| SCNN1B | -1.18606 | 0.068863 | 2.288243 | -17.2235 | 2.87E-54 | 4.75E-52 | 112.6696 |
| MYRIP | -0.99761 | 0.05794 | 2.756779 | -17.218 | 3.06E-54 | 5.01E-52 | 112.6064 |
| AQP4 | -1.47856 | 0.085887 | 1.74429 | -17.2152 | 3.16E-54 | 5.13E-52 | 112.5745 |
| KCNE2 | -2.52374 | 0.146864 | 3.882424 | -17.1842 | 4.53E-54 | 7.27E-52 | 112.2188 |
| BID | 0.601704 | 0.035217 | 4.398026 | 17.08545 | 1.41E-53 | 2.23E-51 | 111.0885 |
| SCN3A | -0.75181 | 0.044073 | 2.542008 | -17.0583 | 1.93E-53 | 3.02E-51 | 110.7776 |
| TPX2 | 1.143944 | 0.067083 | 3.989782 | 17.05258 | 2.06E-53 | 3.19E-51 | 110.7126 |
| CCKBR | -1.68896 | 0.099223 | 2.560347 | -17.0218 | 2.94E-53 | 4.47E-51 | 110.3608 |
| PMCH | 0.655241 | 0.038508 | 2.209876 | 17.01592 | 3.14E-53 | 4.74E-51 | 110.2938 |
| CLDN7 | 1.402993 | 0.082629 | 4.678169 | 16.97936 | 4.78E-53 | 7.03E-51 | 109.8765 |
| CNTN3 | -1.14281 | 0.067317 | 1.902553 | -16.9766 | 4.94E-53 | 7.20E-51 | 109.8452 |
| THSD4 | -0.6311 | 0.037197 | 2.454569 | -16.9666 | 5.54E-53 | 7.94E-51 | 109.7305 |
| KIAA2022 | -0.84864 | 0.050031 | 1.926128 | -16.9623 | 5.82E-53 | 8.28E-51 | 109.6817 |
| SERPINE1 | 1.180407 | 0.069916 | 3.161029 | 16.8833 | 1.44E-52 | 2.00E-50 | 108.7814 |
| UBE2S | 0.641772 | 0.03802 | 4.475302 | 16.88002 | 1.50E-52 | 2.04E-50 | 108.744 |
| AJUBA | 0.667363 | 0.039644 | 2.920958 | 16.83392 | 2.54E-52 | 3.44E-50 | 108.2193 |
| MYOC | -1.80494 | 0.107505 | 3.227422 | -16.7893 | 4.23E-52 | 5.69E-50 | 107.7121 |
| TIMP1 | 1.006318 | 0.06 | 6.109641 | 16.77197 | 5.16E-52 | 6.88E-50 | 107.5149 |
| PPP2R3A | -0.80235 | 0.047973 | 3.634915 | -16.7251 | 8.81E-52 | 1.15E-49 | 106.9825 |
| RPRM | -1.13534 | 0.067923 | 2.623278 | -16.7151 | 9.87E-52 | 1.28E-49 | 106.8696 |
| STIL | 0.911936 | 0.054669 | 3.539258 | 16.68097 | 1.46E-51 | 1.83E-49 | 106.4819 |
| ADAMTS12 | 0.681311 | 0.040893 | 2.531759 | 16.66074 | 1.84E-51 | 2.25E-49 | 106.2525 |
| SLC25A4 | -0.69436 | 0.041849 | 3.785594 | -16.5919 | 4.03E-51 | 4.82E-49 | 105.4727 |
| BUB1 | 0.88852 | 0.053691 | 2.432692 | 16.5489 | 6.57E-51 | 7.76E-49 | 104.9864 |
| STK32B | -0.6485 | 0.039243 | 2.667296 | -16.5252 | 8.61E-51 | 1.01E-48 | 104.7185 |
| CXCL1 | 1.511717 | 0.091549 | 4.484646 | 16.5127 | 9.93E-51 | 1.14E-48 | 104.5772 |
| NTN4 | -0.84048 | 0.050947 | 4.268897 | -16.497 | 1.19E-50 | 1.35E-48 | 104.4002 |
| MCM2 | 0.726561 | 0.044085 | 4.223838 | 16.48104 | 1.42E-50 | 1.61E-48 | 104.2196 |
| COL1A1 | 1.164133 | 0.070636 | 4.926853 | 16.48077 | 1.43E-50 | 1.61E-48 | 104.2165 |
| DTL | 0.889371 | 0.054061 | 3.624552 | 16.45127 | 2.00E-50 | 2.22E-48 | 103.8835 |
| SNX10 | 0.923278 | 0.056297 | 3.805327 | 16.40009 | 3.57E-50 | 3.89E-48 | 103.3065 |
| FBXL13 | -0.87397 | 0.053299 | 2.314701 | -16.3976 | 3.67E-50 | 3.98E-48 | 103.278 |
| ANO5 | -1.03972 | 0.063435 | 3.206399 | -16.3902 | 3.99E-50 | 4.30E-48 | 103.195 |
| EXO1 | 0.951487 | 0.058089 | 2.971065 | 16.37981 | 4.49E-50 | 4.81E-48 | 103.078 |
| GIF | -3.5257 | 0.215438 | 3.641137 | -16.3653 | 5.30E-50 | 5.60E-48 | 102.9141 |
| HOXC6 | 1.523243 | 0.0933 | 3.257102 | 16.32628 | 8.24E-50 | 8.61E-48 | 102.4754 |
| KCNJ16 | -2.00997 | 0.12361 | 2.342124 | -16.2606 | 1.73E-49 | 1.79E-47 | 101.7369 |
| KIF14 | 1.086906 | 0.066914 | 3.092958 | 16.24334 | 2.11E-49 | 2.15E-47 | 101.5432 |
| DISP1 | -0.64032 | 0.039432 | 3.546762 | -16.2386 | 2.22E-49 | 2.25E-47 | 101.4904 |
| GINS4 | 0.600466 | 0.036987 | 2.486691 | 16.23437 | 2.33E-49 | 2.35E-47 | 101.4425 |
| UPP1 | 0.716177 | 0.04414 | 3.53381 | 16.22504 | 2.59E-49 | 2.60E-47 | 101.3378 |
| SYT4 | -1.08163 | 0.066704 | 2.382434 | -16.2153 | 2.89E-49 | 2.88E-47 | 101.2287 |
| KIF2C | 0.854134 | 0.052688 | 3.734803 | 16.21103 | 3.04E-49 | 2.99E-47 | 101.1804 |
| CLDN4 | 0.792847 | 0.048918 | 3.267502 | 16.20774 | 3.15E-49 | 3.09E-47 | 101.1436 |
| CACNA2D2 | -0.58887 | 0.036452 | 3.303375 | -16.155 | 5.72E-49 | 5.47E-47 | 100.5518 |
| LAIR2 | 1.035275 | 0.064086 | 2.779441 | 16.15459 | 5.74E-49 | 5.47E-47 | 100.5477 |
| ESM1 | 0.84042 | 0.052217 | 2.198191 | 16.09488 | 1.13E-48 | 1.06E-46 | 99.87909 |
| SST | -2.61022 | 0.162276 | 3.802194 | -16.085 | 1.26E-48 | 1.17E-46 | 99.76898 |
| CKB | -1.31212 | 0.081576 | 4.646755 | -16.0845 | 1.26E-48 | 1.17E-46 | 99.76296 |
| IGF2BP3 | 1.101741 | 0.068538 | 2.867271 | 16.07482 | 1.41E-48 | 1.30E-46 | 99.65466 |
| CDCA5 | 0.732299 | 0.045594 | 3.433336 | 16.06137 | 1.64E-48 | 1.50E-46 | 99.50437 |
| PRIMA1 | -1.12494 | 0.070315 | 3.54249 | -15.9987 | 3.32E-48 | 2.92E-46 | 98.80457 |
| PSAPL1 | -1.98909 | 0.12474 | 3.591397 | -15.9459 | 6.01E-48 | 5.24E-46 | 98.21561 |
| ACACB | -0.61879 | 0.038819 | 3.370771 | -15.9403 | 6.40E-48 | 5.55E-46 | 98.15309 |
| SUCLG2 | -0.59723 | 0.037601 | 5.27169 | -15.8834 | 1.21E-47 | 1.03E-45 | 97.51853 |
| SPP1 | 1.495139 | 0.094155 | 3.398902 | 15.87961 | 1.26E-47 | 1.07E-45 | 97.47693 |
| CENPF | 0.830752 | 0.052383 | 2.920429 | 15.85917 | 1.59E-47 | 1.33E-45 | 97.24953 |
| LEF1 | 0.654083 | 0.041271 | 3.044522 | 15.8485 | 1.79E-47 | 1.48E-45 | 97.13083 |
| CDC25B | 0.654624 | 0.041326 | 4.629045 | 15.84059 | 1.96E-47 | 1.60E-45 | 97.04291 |
| LGI1 | -0.92341 | 0.058485 | 1.784854 | -15.7889 | 3.49E-47 | 2.82E-45 | 96.46819 |
| PHYHD1 | -0.61018 | 0.038749 | 3.069028 | -15.7469 | 5.58E-47 | 4.46E-45 | 96.00256 |
| NCAM1 | -0.84325 | 0.053562 | 2.588564 | -15.7435 | 5.80E-47 | 4.59E-45 | 95.9649 |
| TMEM100 | -1.3199 | 0.084054 | 3.791424 | -15.703 | 9.11E-47 | 7.12E-45 | 95.5163 |
| PTPRZ1 | -1.21772 | 0.077567 | 3.027779 | -15.699 | 9.53E-47 | 7.41E-45 | 95.47154 |
| SOSTDC1 | -2.04354 | 0.130189 | 3.674319 | -15.6967 | 9.77E-47 | 7.57E-45 | 95.44658 |
| TRIP13 | 0.853789 | 0.054408 | 3.583622 | 15.69231 | 1.03E-46 | 7.91E-45 | 95.39747 |
| TMEM27 | -1.08661 | 0.069369 | 2.268283 | -15.6642 | 1.40E-46 | 1.07E-44 | 95.08577 |
| MEST | 0.893949 | 0.057128 | 4.677457 | 15.64822 | 1.68E-46 | 1.27E-44 | 94.90938 |
| SORBS2 | -0.61961 | 0.039669 | 3.024074 | -15.6197 | 2.31E-46 | 1.72E-44 | 94.59373 |
| GSTA3 | -0.93815 | 0.06007 | 2.351577 | -15.6175 | 2.36E-46 | 1.76E-44 | 94.56989 |
| GHR | -1.1141 | 0.071415 | 2.867605 | -15.6004 | 2.86E-46 | 2.11E-44 | 94.3811 |
| UBE2C | 1.121207 | 0.07193 | 4.785715 | 15.58747 | 3.30E-46 | 2.42E-44 | 94.23796 |
| MICALL1 | -0.63702 | 0.040922 | 4.073937 | -15.5667 | 4.16E-46 | 3.02E-44 | 94.0084 |
| NPY | -0.89563 | 0.057562 | 3.028064 | -15.5594 | 4.51E-46 | 3.24E-44 | 93.92839 |
| WNT2 | 1.036753 | 0.066645 | 2.564942 | 15.55633 | 4.66E-46 | 3.34E-44 | 93.8942 |
| HOMER2 | -1.10925 | 0.071353 | 2.597148 | -15.546 | 5.23E-46 | 3.72E-44 | 93.77981 |
| ID4 | -0.64301 | 0.041367 | 3.484938 | -15.5439 | 5.35E-46 | 3.79E-44 | 93.75722 |
| FMO6P | -1.12211 | 0.072363 | 2.22824 | -15.5068 | 8.09E-46 | 5.59E-44 | 93.34755 |
| IL13RA2 | 1.140529 | 0.073564 | 2.450791 | 15.50381 | 8.36E-46 | 5.75E-44 | 93.31501 |
| CCDC110 | -0.65481 | 0.042323 | 1.91446 | -15.4718 | 1.19E-45 | 8.11E-44 | 92.96273 |
| CKAP2L | 0.953711 | 0.061663 | 3.088986 | 15.46645 | 1.27E-45 | 8.57E-44 | 92.90353 |
| GCNT2 | -0.61642 | 0.040045 | 2.71979 | -15.3932 | 2.85E-45 | 1.88E-43 | 92.09814 |
| TOX | -0.74886 | 0.04885 | 3.204719 | -15.3298 | 5.74E-45 | 3.72E-43 | 91.40223 |
| PCSK2 | -0.95633 | 0.062441 | 2.05244 | -15.3156 | 6.71E-45 | 4.33E-43 | 91.24657 |
| CCKAR | -0.85882 | 0.056102 | 2.333446 | -15.3082 | 7.29E-45 | 4.68E-43 | 91.16517 |
| SCARA5 | -0.67657 | 0.044262 | 3.171627 | -15.2856 | 9.35E-45 | 5.97E-43 | 90.9174 |
| IL32 | 0.87411 | 0.057237 | 4.496075 | 15.27166 | 1.09E-44 | 6.93E-43 | 90.7651 |
| ABCA8 | -0.74813 | 0.049013 | 2.634886 | -15.2638 | 1.19E-44 | 7.53E-43 | 90.67928 |
| LPHN3 | -0.67555 | 0.044264 | 2.575111 | -15.2619 | 1.21E-44 | 7.64E-43 | 90.65769 |
| MFI2 | 0.67676 | 0.044345 | 2.393412 | 15.26142 | 1.22E-44 | 7.65E-43 | 90.65295 |
| HOXC9 | 1.324792 | 0.087115 | 2.256635 | 15.20743 | 2.21E-44 | 1.34E-42 | 90.06247 |
| KIF23 | 0.756768 | 0.049792 | 2.873825 | 15.1985 | 2.44E-44 | 1.47E-42 | 89.96499 |
| ECT2 | 0.602913 | 0.039719 | 3.154097 | 15.17961 | 3.00E-44 | 1.80E-42 | 89.75863 |
| ATAD2 | 0.636 | 0.041902 | 3.788404 | 15.17811 | 3.05E-44 | 1.83E-42 | 89.74223 |
| SLC2A12 | -1.00769 | 0.066438 | 2.803091 | -15.1675 | 3.43E-44 | 2.05E-42 | 89.62592 |
| NDC80 | 0.750339 | 0.049487 | 3.998778 | 15.16244 | 3.63E-44 | 2.16E-42 | 89.57115 |
| TACC3 | 0.618912 | 0.040884 | 3.609267 | 15.13824 | 4.73E-44 | 2.78E-42 | 89.30713 |
| SLC39A10 | 0.592805 | 0.039186 | 3.77961 | 15.1278 | 5.31E-44 | 3.09E-42 | 89.19331 |
| NR3C2 | -0.80988 | 0.05359 | 3.997075 | -15.1125 | 6.28E-44 | 3.64E-42 | 89.02686 |
| MCM10 | 0.713844 | 0.047236 | 2.749828 | 15.11234 | 6.29E-44 | 3.64E-42 | 89.02481 |
| DIO2 | 0.661758 | 0.043806 | 3.128911 | 15.10642 | 6.71E-44 | 3.87E-42 | 88.96033 |
| P2RY14 | -0.93631 | 0.062093 | 4.095739 | -15.0793 | 9.04E-44 | 5.16E-42 | 88.66491 |
| CENPW | 0.645683 | 0.042834 | 4.671551 | 15.07419 | 9.56E-44 | 5.42E-42 | 88.60924 |
| SLC16A7 | -0.7936 | 0.052813 | 2.961417 | -15.0266 | 1.61E-43 | 8.90E-42 | 88.09172 |
| ANKRD29 | -0.83602 | 0.055694 | 3.295963 | -15.0109 | 1.91E-43 | 1.05E-41 | 87.92052 |
| FAP | 1.378821 | 0.091859 | 3.926285 | 15.01021 | 1.93E-43 | 1.05E-41 | 87.91352 |
| WISP1 | 0.905688 | 0.060518 | 2.468942 | 14.96561 | 3.14E-43 | 1.70E-41 | 87.42922 |
| CD36 | -0.76751 | 0.051301 | 3.397519 | -14.9608 | 3.31E-43 | 1.79E-41 | 87.37717 |
| AMPD1 | -1.19799 | 0.08018 | 2.672654 | -14.9412 | 4.10E-43 | 2.21E-41 | 87.1649 |
| SLC7A8 | -0.58711 | 0.039297 | 3.253591 | -14.9403 | 4.14E-43 | 2.22E-41 | 87.15438 |
| TREM2 | 0.770019 | 0.051544 | 3.413662 | 14.93896 | 4.20E-43 | 2.25E-41 | 87.1402 |
| KANK4 | -0.96594 | 0.064751 | 2.723774 | -14.9178 | 5.29E-43 | 2.82E-41 | 86.91059 |
| AURKB | 0.907152 | 0.060864 | 2.58876 | 14.90461 | 6.11E-43 | 3.22E-41 | 86.76792 |
| NUF2 | 1.084721 | 0.072852 | 3.480834 | 14.88945 | 7.21E-43 | 3.76E-41 | 86.60383 |
| COL4A1 | 0.945287 | 0.063498 | 5.21312 | 14.88684 | 7.42E-43 | 3.83E-41 | 86.57555 |
| UBE2T | 0.8274 | 0.05571 | 4.265704 | 14.85189 | 1.09E-42 | 5.56E-41 | 86.19745 |
| PLEKHG4 | 0.600197 | 0.040488 | 3.446257 | 14.82425 | 1.47E-42 | 7.49E-41 | 85.89867 |
| SLC1A2 | -0.90774 | 0.061241 | 1.934857 | -14.8225 | 1.50E-42 | 7.61E-41 | 85.87948 |
| SLC5A7 | -0.62811 | 0.04239 | 1.725862 | -14.8175 | 1.58E-42 | 8.01E-41 | 85.82557 |
| FANCI | 0.687344 | 0.046399 | 3.711651 | 14.81367 | 1.65E-42 | 8.30E-41 | 85.7844 |
| FAM189A2 | -0.85125 | 0.057545 | 3.411388 | -14.7927 | 2.07E-42 | 1.04E-40 | 85.55803 |
| RDH12 | -1.51509 | 0.102619 | 2.929437 | -14.7641 | 2.82E-42 | 1.40E-40 | 85.2497 |
| PDGFD | -0.75106 | 0.051043 | 3.376194 | -14.7141 | 4.85E-42 | 2.36E-40 | 84.71107 |
| HPSE | 0.767624 | 0.052242 | 3.063386 | 14.69356 | 6.07E-42 | 2.92E-40 | 84.48966 |
| NEK2 | 1.080513 | 0.073552 | 3.11977 | 14.69057 | 6.27E-42 | 3.01E-40 | 84.45746 |
| AKR1C1 | -0.81076 | 0.055222 | 3.791393 | -14.6818 | 6.89E-42 | 3.30E-40 | 84.36323 |
| WNT5A | 0.666107 | 0.045437 | 4.188329 | 14.65994 | 8.73E-42 | 4.15E-40 | 84.12816 |
| PACRG | -0.66949 | 0.045774 | 2.310102 | -14.6259 | 1.26E-41 | 5.89E-40 | 83.76222 |
| GSG2 | 0.691164 | 0.047343 | 2.135193 | 14.59917 | 1.69E-41 | 7.76E-40 | 83.47563 |
| BGN | 1.043153 | 0.071492 | 3.882132 | 14.59127 | 1.84E-41 | 8.40E-40 | 83.3909 |
| COL5A2 | 0.807611 | 0.055361 | 5.24015 | 14.58796 | 1.90E-41 | 8.69E-40 | 83.35539 |
| SHISA6 | -0.85823 | 0.058877 | 2.087695 | -14.5768 | 2.14E-41 | 9.77E-40 | 83.23624 |
| MMP3 | 1.600605 | 0.109973 | 3.937864 | 14.55455 | 2.73E-41 | 1.23E-39 | 82.99732 |
| CHGA | -1.93373 | 0.13298 | 4.336073 | -14.5414 | 3.14E-41 | 1.41E-39 | 82.85703 |
| HOXA10 | 1.002025 | 0.068961 | 3.376217 | 14.53037 | 3.54E-41 | 1.57E-39 | 82.73846 |
| CYP27B1 | 0.744425 | 0.051284 | 2.520245 | 14.51583 | 4.14E-41 | 1.83E-39 | 82.58287 |
| KRT24 | -1.00209 | 0.069057 | 2.051008 | -14.5111 | 4.36E-41 | 1.92E-39 | 82.53175 |
| FUT9 | -1.12909 | 0.077839 | 2.053367 | -14.5054 | 4.63E-41 | 2.04E-39 | 82.47164 |
| ARRDC4 | -0.62171 | 0.042888 | 4.629998 | -14.4961 | 5.13E-41 | 2.25E-39 | 82.37146 |
| FAM150B | -1.04647 | 0.072194 | 2.297423 | -14.4953 | 5.17E-41 | 2.26E-39 | 82.3637 |
| HMGB3 | 0.621035 | 0.042878 | 3.910553 | 14.48374 | 5.85E-41 | 2.55E-39 | 82.23974 |
| KIF4A | 0.901281 | 0.062282 | 3.392908 | 14.47107 | 6.71E-41 | 2.90E-39 | 82.1044 |
| COL10A1 | 1.500635 | 0.103898 | 3.548322 | 14.44333 | 9.04E-41 | 3.88E-39 | 81.80818 |
| NEDD4L | -0.66125 | 0.04583 | 3.837296 | -14.4284 | 1.06E-40 | 4.55E-39 | 81.64868 |
| APOC1 | 1.080008 | 0.074989 | 4.587107 | 14.40214 | 1.41E-40 | 6.00E-39 | 81.36894 |
| PRC1 | 0.78585 | 0.054635 | 4.490124 | 14.38362 | 1.72E-40 | 7.27E-39 | 81.17161 |
| IL11 | 1.061203 | 0.073817 | 2.525301 | 14.37617 | 1.86E-40 | 7.83E-39 | 81.09223 |
| DUSP26 | -0.62159 | 0.043239 | 3.229945 | -14.3756 | 1.87E-40 | 7.86E-39 | 81.08628 |
| HMP19 | -0.71848 | 0.049997 | 2.382635 | -14.3705 | 1.98E-40 | 8.29E-39 | 81.03166 |
| RAB27A | -0.73829 | 0.051413 | 4.674288 | -14.3602 | 2.21E-40 | 9.19E-39 | 80.92182 |
| ZNF469 | 0.605822 | 0.042236 | 3.598482 | 14.34384 | 2.63E-40 | 1.09E-38 | 80.74818 |
| BRCA2 | 0.613173 | 0.042757 | 2.260159 | 14.34084 | 2.72E-40 | 1.12E-38 | 80.71629 |
| KIF20A | 0.871027 | 0.060754 | 3.463277 | 14.337 | 2.83E-40 | 1.17E-38 | 80.67537 |
| THY1 | 1.020787 | 0.071204 | 4.578575 | 14.33618 | 2.86E-40 | 1.17E-38 | 80.66671 |
| C11orf92 | -1.23799 | 0.086421 | 2.967468 | -14.3251 | 3.22E-40 | 1.32E-38 | 80.54898 |
| FCGR1B | 0.993428 | 0.069354 | 3.700656 | 14.32396 | 3.26E-40 | 1.33E-38 | 80.53682 |
| ULBP2 | 0.715367 | 0.049976 | 2.552738 | 14.31407 | 3.62E-40 | 1.47E-38 | 80.43167 |
| FNDC5 | -0.76273 | 0.053325 | 2.760251 | -14.3034 | 4.06E-40 | 1.64E-38 | 80.31874 |
| SPC25 | 1.119475 | 0.078338 | 3.085953 | 14.29026 | 4.67E-40 | 1.88E-38 | 80.17879 |
| TMED6 | -1.73116 | 0.121321 | 3.154762 | -14.2693 | 5.84E-40 | 2.32E-38 | 79.95658 |
| GPX3 | -0.77917 | 0.054635 | 4.300867 | -14.2614 | 6.36E-40 | 2.51E-38 | 79.87223 |
| TOP2A | 0.923982 | 0.064819 | 3.554675 | 14.25479 | 6.82E-40 | 2.67E-38 | 79.80236 |
| MFAP2 | 0.984266 | 0.069169 | 3.8006 | 14.22985 | 8.91E-40 | 3.46E-38 | 79.53805 |
| HPGD | -1.41401 | 0.099389 | 4.52273 | -14.227 | 9.19E-40 | 3.57E-38 | 79.50746 |
| ZNF385B | -1.08935 | 0.076591 | 2.200784 | -14.2229 | 9.60E-40 | 3.72E-38 | 79.46395 |
| RAI2 | -0.75721 | 0.053278 | 3.919788 | -14.2125 | 1.07E-39 | 4.13E-38 | 79.35388 |
| ZNF204P | -0.68451 | 0.048168 | 3.267308 | -14.2109 | 1.09E-39 | 4.20E-38 | 79.33716 |
| AGPAT9 | -0.85925 | 0.060515 | 3.84273 | -14.1988 | 1.24E-39 | 4.73E-38 | 79.2093 |
| FOXM1 | 0.848882 | 0.059834 | 3.524674 | 14.18727 | 1.40E-39 | 5.33E-38 | 79.08724 |
| BIRC5 | 0.927783 | 0.06546 | 3.756582 | 14.17326 | 1.63E-39 | 6.17E-38 | 78.939 |
| PLP1 | -1.27182 | 0.090014 | 2.784007 | -14.1292 | 2.60E-39 | 9.65E-38 | 78.47349 |
| POLQ | 0.716925 | 0.05075 | 2.98878 | 14.12664 | 2.68E-39 | 9.90E-38 | 78.44638 |
| KL | -0.69712 | 0.049403 | 2.283065 | -14.111 | 3.16E-39 | 1.16E-37 | 78.28135 |
| ANLN | 0.946879 | 0.067106 | 3.743172 | 14.11025 | 3.19E-39 | 1.17E-37 | 78.27343 |
| CHGB | -1.43267 | 0.10154 | 2.843846 | -14.1094 | 3.21E-39 | 1.18E-37 | 78.26437 |
| CDCA2 | 0.929744 | 0.066105 | 3.219472 | 14.06456 | 5.18E-39 | 1.87E-37 | 77.79176 |
| SCUBE2 | -0.88262 | 0.06287 | 3.126767 | -14.0387 | 6.81E-39 | 2.44E-37 | 77.51951 |
| CAPN13 | -1.24747 | 0.088998 | 2.875558 | -14.0168 | 8.59E-39 | 3.07E-37 | 77.28864 |
| SORCS1 | -0.73681 | 0.052641 | 2.111195 | -13.9968 | 1.06E-38 | 3.78E-37 | 77.07868 |
| MYOT | -1.01834 | 0.072775 | 2.274672 | -13.9929 | 1.11E-38 | 3.92E-37 | 77.03829 |
| LONRF2 | -0.92752 | 0.06629 | 2.826721 | -13.9918 | 1.12E-38 | 3.96E-37 | 77.0267 |
| ISL1 | -0.89379 | 0.063921 | 3.852167 | -13.9827 | 1.23E-38 | 4.36E-37 | 76.93087 |
| TREM1 | 0.974608 | 0.069702 | 3.051547 | 13.98242 | 1.24E-38 | 4.36E-37 | 76.92768 |
| MDK | 0.671975 | 0.048246 | 4.673147 | 13.92799 | 2.20E-38 | 7.65E-37 | 76.35654 |
| SHCBP1 | 0.760535 | 0.054605 | 3.116965 | 13.92797 | 2.20E-38 | 7.65E-37 | 76.35641 |
| SPAG5 | 0.756724 | 0.054338 | 3.862356 | 13.92626 | 2.24E-38 | 7.77E-37 | 76.33849 |
| CXCL6 | 1.1149 | 0.080141 | 2.475414 | 13.91173 | 2.61E-38 | 9.02E-37 | 76.18623 |
| FAM64A | 0.710197 | 0.051124 | 3.122813 | 13.89158 | 3.23E-38 | 1.11E-36 | 75.97514 |
| FGF14 | -0.62597 | 0.045169 | 1.883897 | -13.8584 | 4.58E-38 | 1.55E-36 | 75.62759 |
| RAD54L | 0.6983 | 0.05041 | 3.276811 | 13.85235 | 4.88E-38 | 1.65E-36 | 75.56477 |
| MKI67 | 0.789188 | 0.057045 | 3.492967 | 13.83446 | 5.90E-38 | 1.97E-36 | 75.37782 |
| CFD | -1.04922 | 0.075878 | 5.233714 | -13.8276 | 6.34E-38 | 2.10E-36 | 75.30639 |
| C2 | 0.591032 | 0.042852 | 3.193597 | 13.79236 | 9.18E-38 | 3.00E-36 | 74.93838 |
| IL2RA | 0.664377 | 0.048183 | 2.59817 | 13.78857 | 9.56E-38 | 3.12E-36 | 74.89885 |
| MFSD4 | -1.366 | 0.099083 | 3.367725 | -13.7864 | 9.78E-38 | 3.18E-36 | 74.87619 |
| SKA3 | 0.735475 | 0.053386 | 3.311651 | 13.77651 | 1.08E-37 | 3.50E-36 | 74.77312 |
| CDC45 | 0.779011 | 0.05676 | 3.551973 | 13.72458 | 1.87E-37 | 5.92E-36 | 74.23231 |
| FAM107A | -0.69454 | 0.050662 | 3.755387 | -13.7093 | 2.20E-37 | 6.88E-36 | 74.07344 |
| OLR1 | 1.018517 | 0.074327 | 2.185491 | 13.70323 | 2.34E-37 | 7.32E-36 | 74.01023 |
| KIF18A | 0.908006 | 0.066406 | 2.798853 | 13.67361 | 3.19E-37 | 9.91E-36 | 73.70252 |
| ORC1 | 0.625806 | 0.045818 | 2.396384 | 13.65854 | 3.74E-37 | 1.15E-35 | 73.54604 |
| CKS2 | 0.797707 | 0.058504 | 5.216244 | 13.63506 | 4.78E-37 | 1.45E-35 | 73.30251 |
| PDPN | 0.594734 | 0.043685 | 3.229526 | 13.61407 | 5.95E-37 | 1.79E-35 | 73.08502 |
| NME5 | -0.6439 | 0.047399 | 2.976068 | -13.5848 | 8.07E-37 | 2.41E-35 | 72.78211 |
| CAPN9 | -1.82613 | 0.134535 | 3.446148 | -13.5736 | 9.07E-37 | 2.70E-35 | 72.66646 |
| FAM149A | -0.67224 | 0.049535 | 3.31722 | -13.571 | 9.32E-37 | 2.77E-35 | 72.63915 |
| ADH1B | -1.10367 | 0.081363 | 3.025522 | -13.5648 | 9.94E-37 | 2.95E-35 | 72.57545 |
| ZNF536 | -0.65615 | 0.048437 | 2.007253 | -13.5464 | 1.20E-36 | 3.54E-35 | 72.38497 |
| HJURP | 0.963109 | 0.07115 | 3.586502 | 13.53639 | 1.34E-36 | 3.90E-35 | 72.28143 |
| AURKA | 0.71928 | 0.053271 | 3.622166 | 13.50227 | 1.91E-36 | 5.53E-35 | 71.92925 |
| SCNN1G | -0.89858 | 0.066724 | 1.715517 | -13.4671 | 2.75E-36 | 7.83E-35 | 71.5671 |
| LYVE1 | -1.06896 | 0.079383 | 3.129469 | -13.4659 | 2.78E-36 | 7.91E-35 | 71.55478 |
| SLC12A8 | 0.62392 | 0.046352 | 3.162531 | 13.46041 | 2.95E-36 | 8.35E-35 | 71.49781 |
| EEF1A2 | -0.91279 | 0.067818 | 3.138613 | -13.4595 | 2.97E-36 | 8.42E-35 | 71.48827 |
| PXMP2 | -0.64191 | 0.047711 | 4.137522 | -13.4541 | 3.14E-36 | 8.86E-35 | 71.43301 |
| KIT | -0.82654 | 0.061528 | 3.986506 | -13.4337 | 3.89E-36 | 1.09E-34 | 71.22247 |
| TNFRSF11B | 1.033077 | 0.07697 | 2.632728 | 13.42181 | 4.40E-36 | 1.22E-34 | 71.10053 |
| GTSE1 | 0.751733 | 0.056055 | 3.152565 | 13.41069 | 4.93E-36 | 1.37E-34 | 70.98624 |
| RERGL | -0.90801 | 0.067923 | 2.546134 | -13.3683 | 7.65E-36 | 2.08E-34 | 70.55124 |
| HOXC10 | 1.137119 | 0.085091 | 2.447182 | 13.3635 | 8.04E-36 | 2.18E-34 | 70.50167 |
| CD300LF | 0.709062 | 0.053068 | 3.144576 | 13.36136 | 8.22E-36 | 2.23E-34 | 70.4798 |
| CDC6 | 0.74772 | 0.055964 | 2.880941 | 13.3607 | 8.28E-36 | 2.24E-34 | 70.47302 |
| PRDM16 | -0.6576 | 0.049244 | 2.870612 | -13.354 | 8.88E-36 | 2.40E-34 | 70.40377 |
| CADM2 | -0.70612 | 0.052937 | 1.661824 | -13.3389 | 1.04E-35 | 2.77E-34 | 70.24939 |
| NUSAP1 | 0.825744 | 0.061913 | 4.020434 | 13.33727 | 1.05E-35 | 2.82E-34 | 70.23273 |
| RAD51 | 0.621124 | 0.046571 | 2.933393 | 13.3371 | 1.06E-35 | 2.82E-34 | 70.23101 |
| OSMR | 0.636089 | 0.047699 | 3.304943 | 13.33548 | 1.07E-35 | 2.86E-34 | 70.21438 |
| PTGER3 | -0.6183 | 0.046371 | 2.55044 | -13.3339 | 1.09E-35 | 2.90E-34 | 70.19856 |
| PIK3AP1 | 0.621541 | 0.046635 | 3.255544 | 13.32791 | 1.16E-35 | 3.07E-34 | 70.13685 |
| ADIPOQ | -1.38168 | 0.103705 | 2.12262 | -13.3232 | 1.22E-35 | 3.21E-34 | 70.08856 |
| RAB37 | -0.6103 | 0.045827 | 3.144437 | -13.3174 | 1.29E-35 | 3.40E-34 | 70.02937 |
| CST1 | 1.634247 | 0.122836 | 3.026414 | 13.30428 | 1.48E-35 | 3.89E-34 | 69.89494 |
| BEX5 | -0.85373 | 0.064208 | 3.821631 | -13.2964 | 1.61E-35 | 4.20E-34 | 69.8139 |
| LAMP3 | 0.917556 | 0.069067 | 3.866836 | 13.28494 | 1.81E-35 | 4.70E-34 | 69.6971 |
| TRIB3 | 0.63148 | 0.047539 | 3.362345 | 13.28352 | 1.84E-35 | 4.76E-34 | 69.68251 |
| C2orf40 | -1.76396 | 0.133095 | 4.457086 | -13.2534 | 2.51E-35 | 6.43E-34 | 69.37444 |
| TMEM220 | -0.64892 | 0.04899 | 3.245484 | -13.2459 | 2.71E-35 | 6.91E-34 | 69.29831 |
| PTTG1 | 0.703121 | 0.053088 | 5.052363 | 13.24449 | 2.75E-35 | 7.01E-34 | 69.28378 |
| KBTBD12 | -0.86786 | 0.065587 | 2.400053 | -13.2322 | 3.12E-35 | 7.92E-34 | 69.15809 |
| IL1A | 0.626722 | 0.047447 | 2.002278 | 13.20877 | 3.96E-35 | 9.98E-34 | 68.91943 |
| SULT2A1 | -1.19115 | 0.090185 | 2.089409 | -13.2078 | 4.00E-35 | 1.01E-33 | 68.90949 |
| IRX3 | -1.13721 | 0.086326 | 3.332195 | -13.1735 | 5.70E-35 | 1.41E-33 | 68.55999 |
| BANF1 | 0.612254 | 0.046483 | 4.620394 | 13.17171 | 5.80E-35 | 1.44E-33 | 68.54185 |
| NFE2L3 | 0.678709 | 0.051552 | 3.709288 | 13.16543 | 6.19E-35 | 1.53E-33 | 68.47793 |
| CPXM1 | 0.908374 | 0.069021 | 3.096134 | 13.16083 | 6.49E-35 | 1.60E-33 | 68.43116 |
| COL12A1 | 0.810541 | 0.061637 | 3.62534 | 13.1503 | 7.23E-35 | 1.77E-33 | 68.32408 |
| BUB1B | 0.841207 | 0.064039 | 3.940912 | 13.13583 | 8.38E-35 | 2.04E-33 | 68.177 |
| CPE | -0.93383 | 0.071105 | 4.686922 | -13.1331 | 8.62E-35 | 2.10E-33 | 68.14873 |
| XK | -1.00896 | 0.076885 | 4.323192 | -13.123 | 9.56E-35 | 2.31E-33 | 68.04689 |
| E2F7 | 0.601243 | 0.045837 | 2.571824 | 13.11684 | 1.02E-34 | 2.45E-33 | 67.98405 |
| BTC | -0.72036 | 0.054922 | 3.196591 | -13.1161 | 1.03E-34 | 2.47E-33 | 67.97679 |
| GPR84 | 0.712757 | 0.054363 | 2.429363 | 13.11114 | 1.08E-34 | 2.59E-33 | 67.92613 |
| RNASE4 | -0.70893 | 0.054149 | 5.059439 | -13.0922 | 1.31E-34 | 3.12E-33 | 67.73363 |
| HMGA1 | 0.604872 | 0.046235 | 4.049524 | 13.08244 | 1.45E-34 | 3.43E-33 | 67.635 |
| FSCN1 | 0.658715 | 0.050355 | 3.622187 | 13.08149 | 1.46E-34 | 3.46E-33 | 67.62534 |
| GPR64 | -1.30401 | 0.099713 | 2.779129 | -13.0777 | 1.52E-34 | 3.59E-33 | 67.58705 |
| DEPDC1B | 0.929279 | 0.071207 | 3.09091 | 13.05041 | 2.01E-34 | 4.71E-33 | 67.31041 |
| GINS1 | 0.85584 | 0.065588 | 4.124377 | 13.04877 | 2.04E-34 | 4.78E-33 | 67.29378 |
| COL4A6 | -0.75566 | 0.057912 | 2.487036 | -13.0484 | 2.05E-34 | 4.79E-33 | 67.28981 |
| F13A1 | -0.90128 | 0.06909 | 4.142508 | -13.045 | 2.12E-34 | 4.95E-33 | 67.25563 |
| TCEAL2 | -1.20514 | 0.092449 | 3.258292 | -13.0358 | 2.33E-34 | 5.43E-33 | 67.16252 |
| LOC100505501 | -0.6738 | 0.051853 | 2.512283 | -12.9944 | 3.56E-34 | 8.15E-33 | 66.74389 |
| GREM2 | -0.94887 | 0.073043 | 2.763736 | -12.9905 | 3.70E-34 | 8.47E-33 | 66.70491 |
| HOXA13 | 1.340361 | 0.10341 | 2.678687 | 12.96162 | 4.97E-34 | 1.12E-32 | 66.41291 |
| PLK4 | 0.601681 | 0.046562 | 2.424752 | 12.92214 | 7.42E-34 | 1.66E-32 | 66.01499 |
| TFAP2A | 0.586919 | 0.045513 | 2.119881 | 12.89576 | 9.69E-34 | 2.16E-32 | 65.74947 |
| HCAR3 | 1.232175 | 0.095564 | 3.217759 | 12.89374 | 9.90E-34 | 2.20E-32 | 65.72917 |
| GPRC5A | 0.902145 | 0.069978 | 4.012119 | 12.89189 | 1.01E-33 | 2.24E-32 | 65.71055 |
| CHIA | -1.886 | 0.146476 | 2.970458 | -12.8758 | 1.19E-33 | 2.62E-32 | 65.54889 |
| EZH2 | 0.753926 | 0.058554 | 3.738267 | 12.87564 | 1.19E-33 | 2.62E-32 | 65.54717 |
| PTN | -0.70702 | 0.054921 | 3.644734 | -12.8735 | 1.22E-33 | 2.67E-32 | 65.5254 |
| WISP2 | -0.82685 | 0.064252 | 3.113628 | -12.8688 | 1.27E-33 | 2.79E-32 | 65.47894 |
| CDC20 | 0.722518 | 0.056174 | 4.083918 | 12.86221 | 1.36E-33 | 2.97E-32 | 65.41224 |
| COL18A1 | 0.683125 | 0.053132 | 4.539963 | 12.85716 | 1.43E-33 | 3.12E-32 | 65.36152 |
| MAD2L1 | 0.674918 | 0.052501 | 3.632619 | 12.85531 | 1.46E-33 | 3.18E-32 | 65.34294 |
| RCAN2 | -0.8578 | 0.066731 | 4.436569 | -12.8546 | 1.47E-33 | 3.20E-32 | 65.33558 |
| LOC400043 | -0.98003 | 0.076255 | 4.713506 | -12.8519 | 1.51E-33 | 3.27E-32 | 65.30914 |
| HOXB9 | 1.036604 | 0.080818 | 2.731953 | 12.82637 | 1.96E-33 | 4.20E-32 | 65.05255 |
| MMP9 | 0.997429 | 0.077819 | 4.390975 | 12.81723 | 2.15E-33 | 4.59E-32 | 64.96088 |
| BCL2A1 | 0.932944 | 0.072866 | 4.006441 | 12.80354 | 2.47E-33 | 5.23E-32 | 64.82367 |
| CBR1 | -0.63305 | 0.049468 | 4.565031 | -12.7973 | 2.63E-33 | 5.57E-32 | 64.76108 |
| SULF1 | 1.195161 | 0.093396 | 4.676509 | 12.79676 | 2.64E-33 | 5.59E-32 | 64.75572 |
| ASPM | 0.891291 | 0.069679 | 2.981476 | 12.79147 | 2.79E-33 | 5.88E-32 | 64.70277 |
| SRPX2 | 0.615113 | 0.048114 | 2.704039 | 12.78437 | 2.99E-33 | 6.30E-32 | 64.63167 |
| PIRT | -0.82883 | 0.065067 | 2.217368 | -12.7381 | 4.77E-33 | 9.93E-32 | 64.16886 |
| FABP4 | -1.12423 | 0.088258 | 2.964054 | -12.738 | 4.78E-33 | 9.93E-32 | 64.16764 |
| PLA2G1B | -0.75759 | 0.059487 | 2.064855 | -12.7355 | 4.90E-33 | 1.02E-31 | 64.14258 |
| F2RL2 | 0.640597 | 0.05035 | 2.77796 | 12.72281 | 5.57E-33 | 1.15E-31 | 64.0163 |
| GKN2 | -3.17565 | 0.249658 | 4.796919 | -12.72 | 5.73E-33 | 1.18E-31 | 63.98822 |
| ZNF300P1 | -0.65974 | 0.051942 | 2.676762 | -12.7017 | 6.89E-33 | 1.41E-31 | 63.80523 |
| CDCA8 | 0.72385 | 0.057032 | 3.987342 | 12.69204 | 7.59E-33 | 1.55E-31 | 63.7094 |
| OXCT1 | -0.65524 | 0.051708 | 4.527596 | -12.672 | 9.28E-33 | 1.89E-31 | 63.51005 |
| COL4A5 | -0.7273 | 0.05749 | 3.340874 | -12.651 | 1.15E-32 | 2.33E-31 | 63.30072 |
| CDKN3 | 0.752479 | 0.059619 | 3.972621 | 12.62154 | 1.54E-32 | 3.11E-31 | 63.00769 |
| LOC340340 | 0.756427 | 0.060023 | 2.15265 | 12.60225 | 1.87E-32 | 3.74E-31 | 62.81605 |
| PDK4 | -0.94346 | 0.074956 | 3.988221 | -12.5869 | 2.18E-32 | 4.34E-31 | 62.66392 |
| KRT80 | 1.075321 | 0.085433 | 2.400702 | 12.5867 | 2.18E-32 | 4.35E-31 | 62.66176 |
| LRRC17 | -0.60482 | 0.048073 | 2.685864 | -12.5813 | 2.30E-32 | 4.57E-31 | 62.6083 |
| CDC25C | 0.692348 | 0.055074 | 2.351766 | 12.57119 | 2.55E-32 | 5.05E-31 | 62.50789 |
| CCNB2 | 0.696123 | 0.055467 | 3.207413 | 12.55031 | 3.14E-32 | 6.20E-31 | 62.30105 |
| KIF15 | 0.756164 | 0.060284 | 3.361547 | 12.54335 | 3.37E-32 | 6.64E-31 | 62.23208 |
| GDF15 | 0.649484 | 0.051849 | 3.401655 | 12.52638 | 3.99E-32 | 7.83E-31 | 62.06414 |
| PMAIP1 | 0.757071 | 0.060542 | 3.654091 | 12.50488 | 4.94E-32 | 9.63E-31 | 61.85154 |
| NETO2 | 0.626726 | 0.050122 | 3.072596 | 12.5041 | 4.98E-32 | 9.69E-31 | 61.84378 |
| PDILT | -1.30464 | 0.104365 | 2.019156 | -12.5008 | 5.15E-32 | 9.99E-31 | 61.81125 |
| KCNJ15 | -0.94832 | 0.075876 | 2.75048 | -12.4982 | 5.28E-32 | 1.02E-30 | 61.7856 |
| F12 | 0.694216 | 0.055592 | 3.103363 | 12.48763 | 5.87E-32 | 1.13E-30 | 61.68115 |
| TDO2 | 0.780822 | 0.062548 | 2.318682 | 12.48351 | 6.12E-32 | 1.17E-30 | 61.64046 |
| GKN1 | -3.19565 | 0.256297 | 5.264907 | -12.4686 | 7.10E-32 | 1.36E-30 | 61.49291 |
| ERCC6L | 0.610549 | 0.048971 | 2.744162 | 12.4676 | 7.17E-32 | 1.37E-30 | 61.48337 |
| CEP55 | 0.939602 | 0.075773 | 3.866103 | 12.40021 | 1.40E-31 | 2.61E-30 | 60.81952 |
| TRIM29 | 1.055184 | 0.085174 | 2.976989 | 12.38857 | 1.57E-31 | 2.91E-30 | 60.70507 |
| EGFL6 | 0.992453 | 0.080192 | 3.384479 | 12.37594 | 1.78E-31 | 3.29E-30 | 60.58097 |
| LOC284578 | -0.66328 | 0.053649 | 2.159642 | -12.3633 | 2.02E-31 | 3.72E-30 | 60.45666 |
| SLAMF8 | 0.625343 | 0.050635 | 3.578857 | 12.35004 | 2.30E-31 | 4.21E-30 | 60.3267 |
| MAOA | -0.74481 | 0.060373 | 4.467292 | -12.3367 | 2.63E-31 | 4.81E-30 | 60.19559 |
| SCN7A | -1.00378 | 0.081434 | 2.344834 | -12.3262 | 2.91E-31 | 5.31E-30 | 60.09323 |
| RBPMS2 | -1.08126 | 0.08773 | 3.864939 | -12.3248 | 2.96E-31 | 5.38E-30 | 60.07922 |
| FOXS1 | 0.70342 | 0.057192 | 2.810024 | 12.2992 | 3.81E-31 | 6.85E-30 | 59.82848 |
| MSR1 | 0.61209 | 0.049822 | 2.791102 | 12.28552 | 4.36E-31 | 7.81E-30 | 59.69469 |
| APOE | 0.774182 | 0.063017 | 4.995273 | 12.28521 | 4.37E-31 | 7.83E-30 | 59.69165 |
| MMP12 | 1.666432 | 0.135737 | 4.410795 | 12.27688 | 4.75E-31 | 8.44E-30 | 59.61018 |
| RAD51AP1 | 0.649722 | 0.052927 | 3.795136 | 12.27572 | 4.80E-31 | 8.52E-30 | 59.59881 |
| CHSY3 | 0.688429 | 0.056092 | 2.628067 | 12.27323 | 4.92E-31 | 8.71E-30 | 59.57451 |
| NCAPG | 0.801658 | 0.065373 | 3.14069 | 12.26274 | 5.46E-31 | 9.61E-30 | 59.47191 |
| GSTM5 | -0.59224 | 0.048388 | 3.384216 | -12.2395 | 6.86E-31 | 1.20E-29 | 59.24454 |
| PTPRN2 | -0.68217 | 0.055792 | 3.624048 | -12.227 | 7.76E-31 | 1.35E-29 | 59.12326 |
| ADORA2B | 0.824506 | 0.067452 | 3.262058 | 12.22365 | 8.02E-31 | 1.39E-29 | 59.09043 |
| CLDN3 | 1.492894 | 0.122144 | 3.855259 | 12.22246 | 8.11E-31 | 1.41E-29 | 59.0788 |
| ITGA8 | -0.73611 | 0.060231 | 3.255639 | -12.2215 | 8.19E-31 | 1.42E-29 | 59.06963 |
| NOX4 | 0.704385 | 0.057675 | 2.056603 | 12.21305 | 8.90E-31 | 1.54E-29 | 58.98702 |
| KLF4 | -0.6706 | 0.054924 | 4.852632 | -12.2096 | 9.21E-31 | 1.59E-29 | 58.95328 |
| SELENBP1 | -0.6958 | 0.057002 | 4.977498 | -12.2067 | 9.48E-31 | 1.63E-29 | 58.92473 |
| CDK1 | 0.800939 | 0.065734 | 3.850235 | 12.18457 | 1.18E-30 | 2.01E-29 | 58.70965 |
| SMAD9 | -0.6003 | 0.049272 | 3.295302 | -12.1832 | 1.19E-30 | 2.04E-29 | 58.69643 |
| LOC643201 | -0.61622 | 0.050676 | 2.299276 | -12.1601 | 1.50E-30 | 2.53E-29 | 58.4713 |
| SFRP1 | -0.92256 | 0.075897 | 3.087242 | -12.1553 | 1.57E-30 | 2.64E-29 | 58.42528 |
| LDHD | -0.73678 | 0.060768 | 3.176932 | -12.1246 | 2.12E-30 | 3.51E-29 | 58.12677 |
| MAP7D2 | -1.26615 | 0.104624 | 2.902262 | -12.1018 | 2.65E-30 | 4.37E-29 | 57.90583 |
| LIPF | -3.19026 | 0.263684 | 4.791744 | -12.0988 | 2.73E-30 | 4.48E-29 | 57.87664 |
| CDT1 | 0.761136 | 0.062939 | 3.252006 | 12.09318 | 2.88E-30 | 4.72E-29 | 57.82215 |
| OVOL1 | 1.009606 | 0.083914 | 3.049901 | 12.03146 | 5.27E-30 | 8.52E-29 | 57.22505 |
| MELK | 0.935956 | 0.077857 | 4.170982 | 12.02152 | 5.80E-30 | 9.36E-29 | 57.12914 |
| SGOL2 | 0.588615 | 0.049029 | 3.059538 | 12.00543 | 6.79E-30 | 1.08E-28 | 56.97379 |
| CCL4 | 0.73245 | 0.061058 | 4.65508 | 11.99598 | 7.44E-30 | 1.18E-28 | 56.88272 |
| MND1 | 0.647231 | 0.054029 | 2.97217 | 11.97932 | 8.75E-30 | 1.38E-28 | 56.72216 |
| GPM6A | -0.73882 | 0.061687 | 2.234044 | -11.9769 | 8.96E-30 | 1.42E-28 | 56.69928 |
| HKDC1 | 0.695587 | 0.058096 | 2.97681 | 11.97305 | 9.30E-30 | 1.47E-28 | 56.66173 |
| CD55 | 0.718902 | 0.060083 | 5.233337 | 11.96518 | 1.00E-29 | 1.58E-28 | 56.58598 |
| TMEM35 | -1.08667 | 0.090856 | 2.789111 | -11.9604 | 1.05E-29 | 1.65E-28 | 56.54011 |
| RSPO2 | -0.98484 | 0.082365 | 1.882116 | -11.9571 | 1.09E-29 | 1.70E-28 | 56.50823 |
| CCNA2 | 0.709478 | 0.059466 | 3.626811 | 11.93078 | 1.40E-29 | 2.18E-28 | 56.25509 |
| IL4I1 | 0.640191 | 0.053701 | 3.387806 | 11.92135 | 1.54E-29 | 2.38E-28 | 56.16453 |
| SRPX | -0.94943 | 0.079723 | 4.217774 | -11.9091 | 1.73E-29 | 2.68E-28 | 56.04686 |
| S100A2 | 0.652942 | 0.054832 | 3.399391 | 11.90798 | 1.75E-29 | 2.70E-28 | 56.03617 |
| SLC28A3 | 0.851942 | 0.071551 | 2.432116 | 11.90683 | 1.77E-29 | 2.73E-28 | 56.02511 |
| ADH1A | -0.86685 | 0.072804 | 3.112354 | -11.9066 | 1.77E-29 | 2.73E-28 | 56.02332 |
| LAMC2 | 0.730774 | 0.061504 | 3.319318 | 11.8818 | 2.25E-29 | 3.45E-28 | 55.78504 |
| VSIG2 | -1.51873 | 0.128004 | 4.327578 | -11.8647 | 2.66E-29 | 4.05E-28 | 55.62136 |
| NAP1L2 | -0.78585 | 0.066242 | 2.391156 | -11.8633 | 2.70E-29 | 4.10E-28 | 55.6074 |
| OIP5 | 0.766855 | 0.064812 | 3.704547 | 11.83204 | 3.65E-29 | 5.50E-28 | 55.30863 |
| RGN | -0.80006 | 0.067716 | 3.551154 | -11.8148 | 4.30E-29 | 6.46E-28 | 55.14396 |
| GJB2 | 0.939583 | 0.079546 | 4.099107 | 11.81184 | 4.43E-29 | 6.64E-28 | 55.11566 |
| CLEC5A | 0.754294 | 0.063878 | 2.045485 | 11.8084 | 4.58E-29 | 6.84E-28 | 55.08275 |
| SPTSSB | -0.65594 | 0.055588 | 2.775685 | -11.8 | 4.97E-29 | 7.41E-28 | 55.00218 |
| DLGAP5 | 0.988613 | 0.08379 | 3.716966 | 11.79866 | 5.03E-29 | 7.48E-28 | 54.9898 |
| SSTR1 | -1.11316 | 0.094379 | 3.038717 | -11.7946 | 5.23E-29 | 7.77E-28 | 54.95076 |
| JAM2 | -0.75174 | 0.063773 | 3.759274 | -11.7878 | 5.59E-29 | 8.29E-28 | 54.88602 |
| SULF2 | 0.592807 | 0.050361 | 4.870831 | 11.77111 | 6.56E-29 | 9.68E-28 | 54.72708 |
| GJB3 | 0.784526 | 0.06677 | 2.831641 | 11.7497 | 8.06E-29 | 1.18E-27 | 54.52318 |
| PI16 | -0.77818 | 0.066243 | 3.830073 | -11.7473 | 8.24E-29 | 1.21E-27 | 54.50077 |
| CKM | -1.17109 | 0.099716 | 2.406912 | -11.7442 | 8.49E-29 | 1.24E-27 | 54.47084 |
| CCNB1 | 0.69481 | 0.059164 | 4.104486 | 11.74385 | 8.52E-29 | 1.24E-27 | 54.46744 |
| OXTR | 0.646143 | 0.055031 | 2.268489 | 11.7414 | 8.73E-29 | 1.27E-27 | 54.44412 |
| BMP5 | -0.63425 | 0.054105 | 2.292867 | -11.7224 | 1.05E-28 | 1.52E-27 | 54.2634 |
| SETBP1 | -0.68566 | 0.05854 | 3.678051 | -11.7127 | 1.15E-28 | 1.66E-27 | 54.17098 |
| SORBS1 | -0.6837 | 0.058468 | 3.734904 | -11.6935 | 1.38E-28 | 1.98E-27 | 53.98913 |
| GC | -1.73041 | 0.148358 | 2.708068 | -11.6637 | 1.84E-28 | 2.60E-27 | 53.70661 |
| ZBTB16 | -0.90371 | 0.077558 | 3.235 | -11.652 | 2.06E-28 | 2.90E-27 | 53.59512 |
| SCG2 | -0.92699 | 0.079651 | 2.83746 | -11.6381 | 2.35E-28 | 3.29E-27 | 53.4637 |
| SCGB2A1 | -1.8405 | 0.158305 | 3.180377 | -11.6262 | 2.63E-28 | 3.67E-27 | 53.3517 |
| GPM6B | -0.87841 | 0.075594 | 3.210254 | -11.6201 | 2.79E-28 | 3.88E-27 | 53.29369 |
| CENPE | 0.650212 | 0.055989 | 3.515936 | 11.61323 | 2.98E-28 | 4.14E-27 | 53.22878 |
| SERPINB5 | 1.294283 | 0.111599 | 3.222182 | 11.59767 | 3.45E-28 | 4.79E-27 | 53.08178 |
| STMN2 | -0.87125 | 0.075243 | 3.060107 | -11.5792 | 4.12E-28 | 5.69E-27 | 52.9075 |
| SLC26A9 | -1.60155 | 0.138323 | 3.300335 | -11.5784 | 4.15E-28 | 5.73E-27 | 52.89957 |
| CCDC146 | -0.6576 | 0.056824 | 3.538328 | -11.5727 | 4.38E-28 | 6.04E-27 | 52.84601 |
| ITGA2 | 0.603414 | 0.052268 | 3.689195 | 11.54462 | 5.72E-28 | 7.82E-27 | 52.58164 |
| PBK | 1.041811 | 0.090706 | 4.131184 | 11.48562 | 1.00E-27 | 1.35E-26 | 52.02708 |
| MMP7 | 1.664402 | 0.145052 | 4.313268 | 11.47454 | 1.11E-27 | 1.49E-26 | 51.92315 |
| FAM19A4 | -1.13158 | 0.098668 | 1.826794 | -11.4685 | 1.18E-27 | 1.58E-26 | 51.86674 |
| CDH19 | -0.87408 | 0.076216 | 2.497537 | -11.4684 | 1.18E-27 | 1.58E-26 | 51.86576 |
| GHRL | -1.35509 | 0.1182 | 3.188559 | -11.4645 | 1.22E-27 | 1.63E-26 | 51.82871 |
| C5 | -0.59532 | 0.051945 | 3.277732 | -11.4606 | 1.27E-27 | 1.69E-26 | 51.79275 |
| CXCL9 | 1.069978 | 0.093394 | 4.564014 | 11.45663 | 1.32E-27 | 1.75E-26 | 51.75531 |
| TMOD1 | -0.62818 | 0.054833 | 3.294881 | -11.4562 | 1.32E-27 | 1.76E-26 | 51.75092 |
| CDHR3 | -0.62378 | 0.054492 | 2.32315 | -11.4472 | 1.44E-27 | 1.91E-26 | 51.66695 |
| SH3BGR | -0.71833 | 0.062773 | 3.270984 | -11.4433 | 1.50E-27 | 1.98E-26 | 51.63043 |
| WDR72 | 0.814933 | 0.071317 | 2.311428 | 11.42696 | 1.74E-27 | 2.29E-26 | 51.47756 |
| SLAIN1 | -0.60086 | 0.052676 | 2.958122 | -11.4066 | 2.11E-27 | 2.76E-26 | 51.28759 |
| MT1M | -1.05346 | 0.092442 | 4.286983 | -11.3959 | 2.34E-27 | 3.05E-26 | 51.18699 |
| ALDH3A1 | -1.29429 | 0.11382 | 3.82664 | -11.3714 | 2.95E-27 | 3.81E-26 | 50.95856 |
| NTN1 | -0.65137 | 0.057328 | 2.969854 | -11.3622 | 3.21E-27 | 4.15E-26 | 50.87254 |
| LINGO2 | -0.66397 | 0.058475 | 2.210869 | -11.3549 | 3.44E-27 | 4.43E-26 | 50.80467 |
| ATP1A2 | -1.04464 | 0.092057 | 2.851591 | -11.3477 | 3.68E-27 | 4.73E-26 | 50.73823 |
| TREH | -0.64948 | 0.057258 | 2.058133 | -11.3429 | 3.85E-27 | 4.94E-26 | 50.69345 |
| CENPA | 0.696385 | 0.061438 | 3.198169 | 11.3347 | 4.16E-27 | 5.33E-26 | 50.6168 |
| FGF13 | -0.72989 | 0.064433 | 3.535974 | -11.3279 | 4.44E-27 | 5.66E-26 | 50.55342 |
| FHL1 | -0.96093 | 0.085092 | 4.350774 | -11.2928 | 6.17E-27 | 7.78E-26 | 50.22713 |
| RELN | -0.67461 | 0.059988 | 2.570135 | -11.2458 | 9.58E-27 | 1.18E-25 | 49.79137 |
| KLK10 | 0.710716 | 0.06321 | 2.612095 | 11.24371 | 9.77E-27 | 1.20E-25 | 49.77239 |
| SKA1 | 0.69194 | 0.061564 | 2.270096 | 11.23933 | 1.02E-26 | 1.25E-25 | 49.73183 |
| EPHA7 | -0.91241 | 0.081273 | 2.106999 | -11.2265 | 1.15E-26 | 1.40E-25 | 49.61363 |
| ABCA6 | -0.58738 | 0.052353 | 2.733558 | -11.2196 | 1.22E-26 | 1.50E-25 | 49.54895 |
| PPP1R1A | -0.6282 | 0.056035 | 2.318397 | -11.2108 | 1.33E-26 | 1.61E-25 | 49.46831 |
| HDC | -0.72088 | 0.064374 | 3.612657 | -11.1984 | 1.49E-26 | 1.81E-25 | 49.35372 |
| WASF3 | -0.68849 | 0.061582 | 3.577204 | -11.1801 | 1.77E-26 | 2.13E-25 | 49.18506 |
| RARRES1 | 1.050239 | 0.094037 | 4.153514 | 11.16833 | 1.97E-26 | 2.37E-25 | 49.07613 |
| PDIA2 | -1.04708 | 0.093862 | 3.087869 | -11.1555 | 2.22E-26 | 2.65E-25 | 48.95839 |
| CLU | -0.68366 | 0.061424 | 4.292329 | -11.1301 | 2.81E-26 | 3.33E-25 | 48.72462 |
| EREG | 0.721369 | 0.064829 | 2.351652 | 11.12724 | 2.89E-26 | 3.42E-25 | 48.69796 |
| ANXA9 | 0.741527 | 0.066662 | 2.576386 | 11.12361 | 2.99E-26 | 3.52E-25 | 48.66458 |
| TTK | 0.908007 | 0.081662 | 3.529997 | 11.1191 | 3.12E-26 | 3.67E-25 | 48.62308 |
| IGFBP6 | -0.72343 | 0.065166 | 4.278 | -11.1012 | 3.68E-26 | 4.29E-25 | 48.45894 |
| CARTPT | -1.06277 | 0.09575 | 2.95397 | -11.0994 | 3.74E-26 | 4.36E-25 | 48.44232 |
| AGMAT | 0.713844 | 0.064488 | 3.179528 | 11.06937 | 4.95E-26 | 5.69E-25 | 48.16684 |
| AKR7A3 | -0.85423 | 0.077194 | 4.922457 | -11.0661 | 5.10E-26 | 5.86E-25 | 48.13702 |
| ETV4 | 0.63646 | 0.057541 | 3.032486 | 11.06102 | 5.34E-26 | 6.12E-25 | 48.09036 |
| FAM46C | -0.67688 | 0.061207 | 4.37346 | -11.0589 | 5.45E-26 | 6.24E-25 | 48.07077 |
| IL24 | 0.670274 | 0.060652 | 2.277538 | 11.05112 | 5.86E-26 | 6.67E-25 | 47.99965 |
| PART1 | -0.62841 | 0.056873 | 2.3058 | -11.0493 | 5.95E-26 | 6.78E-25 | 47.98324 |
| MEIS2 | -0.64113 | 0.058154 | 4.643304 | -11.0248 | 7.47E-26 | 8.43E-25 | 47.75927 |
| BLM | 0.586489 | 0.053251 | 3.513658 | 11.01372 | 8.27E-26 | 9.28E-25 | 47.65781 |
| MUM1L1 | -0.76799 | 0.069805 | 1.849551 | -11.002 | 9.21E-26 | 1.03E-24 | 47.55102 |
| GATA5 | -1.15275 | 0.104855 | 2.594235 | -10.9938 | 9.94E-26 | 1.11E-24 | 47.47607 |
| NPTX1 | -0.9682 | 0.088132 | 3.040786 | -10.9858 | 1.07E-25 | 1.19E-24 | 47.4027 |
| PGM5 | -0.78069 | 0.071117 | 3.07798 | -10.9776 | 1.15E-25 | 1.28E-24 | 47.3287 |
| PLIN1 | -0.95044 | 0.086603 | 2.705436 | -10.9748 | 1.18E-25 | 1.32E-24 | 47.30234 |
| MARVELD3 | 0.759978 | 0.069373 | 2.942046 | 10.95494 | 1.42E-25 | 1.57E-24 | 47.12192 |
| KLK6 | 1.091944 | 0.099692 | 2.825276 | 10.95323 | 1.44E-25 | 1.59E-24 | 47.10636 |
| COL11A1 | 0.833391 | 0.076223 | 2.072944 | 10.93359 | 1.73E-25 | 1.90E-24 | 46.9278 |
| MYH11 | -1.11674 | 0.102251 | 4.671415 | -10.9215 | 1.93E-25 | 2.11E-24 | 46.81802 |
| PRKAA2 | -0.66574 | 0.061114 | 2.400921 | -10.8933 | 2.50E-25 | 2.72E-24 | 46.5624 |
| OLFML2B | 0.736854 | 0.067656 | 3.811004 | 10.89122 | 2.55E-25 | 2.76E-24 | 46.54319 |
| FOXF2 | -0.69495 | 0.064062 | 4.024127 | -10.8481 | 3.78E-25 | 4.05E-24 | 46.15334 |
| B3GAT1 | -0.85397 | 0.078867 | 2.889827 | -10.8279 | 4.55E-25 | 4.84E-24 | 45.96996 |
| XKR4 | -0.80811 | 0.07469 | 2.102546 | -10.8195 | 4.91E-25 | 5.20E-24 | 45.89441 |
| C14orf132 | -0.67503 | 0.062439 | 3.527031 | -10.8109 | 5.31E-25 | 5.61E-24 | 45.8173 |
| PSCA | -1.96475 | 0.181793 | 4.476614 | -10.8076 | 5.48E-25 | 5.77E-24 | 45.78754 |
| SCGN | -0.87571 | 0.081065 | 3.071171 | -10.8026 | 5.73E-25 | 6.03E-24 | 45.74234 |
| PRKAR2B | -0.68827 | 0.063887 | 4.165816 | -10.7732 | 7.49E-25 | 7.80E-24 | 45.47701 |
| MNX1 | 0.95679 | 0.088883 | 3.463581 | 10.76464 | 8.10E-25 | 8.40E-24 | 45.40033 |
| VLDLR | -0.66404 | 0.061746 | 3.063698 | -10.7543 | 8.90E-25 | 9.19E-24 | 45.30728 |
| HLF | -0.89054 | 0.082824 | 2.796275 | -10.7522 | 9.07E-25 | 9.35E-24 | 45.28863 |
| CXCL10 | 0.95725 | 0.08916 | 4.275084 | 10.73629 | 1.05E-24 | 1.08E-23 | 45.14563 |
| CHRDL1 | -1.13655 | 0.105957 | 3.580337 | -10.7265 | 1.15E-24 | 1.17E-23 | 45.05768 |
| RRM2 | 0.749563 | 0.069922 | 4.58523 | 10.71993 | 1.22E-24 | 1.24E-23 | 44.99875 |
| KIFC1 | 0.641209 | 0.059816 | 3.539595 | 10.71968 | 1.22E-24 | 1.24E-23 | 44.99656 |
| PKIB | -1.06295 | 0.09919 | 3.159918 | -10.7163 | 1.26E-24 | 1.28E-23 | 44.96613 |
| LDB3 | -0.60588 | 0.05658 | 2.824646 | -10.7085 | 1.35E-24 | 1.37E-23 | 44.89632 |
| DRD5 | -1.05691 | 0.098785 | 2.364247 | -10.6991 | 1.47E-24 | 1.49E-23 | 44.8124 |
| TNFSF4 | 0.631297 | 0.059434 | 2.892653 | 10.62177 | 2.95E-24 | 2.94E-23 | 44.1212 |
| NEB | 0.588177 | 0.055377 | 2.10091 | 10.62139 | 2.96E-24 | 2.95E-23 | 44.1178 |
| ITIH5 | -0.64246 | 0.060503 | 3.151207 | -10.6187 | 3.03E-24 | 3.02E-23 | 44.09393 |
| ARMCX1 | -0.59452 | 0.056047 | 4.358193 | -10.6076 | 3.35E-24 | 3.32E-23 | 43.99501 |
| MMRN1 | -0.73675 | 0.069721 | 2.797385 | -10.5671 | 4.83E-24 | 4.73E-23 | 43.63475 |
| NCF2 | 0.588465 | 0.05571 | 3.687757 | 10.56298 | 5.01E-24 | 4.91E-23 | 43.59817 |
| MFAP5 | -0.8188 | 0.077527 | 4.020984 | -10.5616 | 5.07E-24 | 4.97E-23 | 43.5856 |
| UGT2B15 | -1.33845 | 0.126801 | 3.408645 | -10.5556 | 5.35E-24 | 5.22E-23 | 43.53259 |
| ZNF415 | -0.58573 | 0.055493 | 2.878243 | -10.5552 | 5.37E-24 | 5.24E-23 | 43.52885 |
| HSD17B6 | -0.59486 | 0.056575 | 3.172922 | -10.5145 | 7.73E-24 | 7.46E-23 | 43.16879 |
| SLC4A11 | 0.633166 | 0.060339 | 3.02496 | 10.49352 | 9.33E-24 | 8.96E-23 | 42.98287 |
| MMP10 | 0.953814 | 0.091081 | 2.694358 | 10.4721 | 1.13E-23 | 1.08E-22 | 42.7937 |
| CELA3B | -0.84505 | 0.080909 | 2.457919 | -10.4445 | 1.45E-23 | 1.37E-22 | 42.54999 |
| GUCA2B | -0.66326 | 0.06352 | 2.639788 | -10.4417 | 1.48E-23 | 1.40E-22 | 42.52591 |
| CASQ2 | -1.02918 | 0.098601 | 3.093632 | -10.4378 | 1.53E-23 | 1.45E-22 | 42.49117 |
| SALL4 | 0.825719 | 0.079255 | 3.087682 | 10.41855 | 1.82E-23 | 1.71E-22 | 42.32183 |
| ZWINT | 0.663824 | 0.063751 | 4.683411 | 10.41284 | 1.92E-23 | 1.80E-22 | 42.27164 |
| TRIM15 | 0.786486 | 0.075548 | 3.303092 | 10.41036 | 1.96E-23 | 1.84E-22 | 42.24986 |
| NEFM | -0.60046 | 0.057755 | 2.096632 | -10.3968 | 2.21E-23 | 2.07E-22 | 42.13113 |
| ANGPTL1 | -0.98323 | 0.094572 | 2.690129 | -10.3966 | 2.21E-23 | 2.07E-22 | 42.12904 |
| CIDEC | -0.58798 | 0.056564 | 3.745182 | -10.3948 | 2.25E-23 | 2.10E-22 | 42.11303 |
| CST2 | 0.774589 | 0.074578 | 2.95541 | 10.3863 | 2.42E-23 | 2.26E-22 | 42.03855 |
| PBLD | -0.74866 | 0.072157 | 3.764901 | -10.3755 | 2.67E-23 | 2.48E-22 | 41.9434 |
| HSPB8 | -0.78639 | 0.075799 | 3.630998 | -10.3748 | 2.69E-23 | 2.50E-22 | 41.93753 |
| C1QTNF7 | -0.77379 | 0.074631 | 2.551457 | -10.3682 | 2.85E-23 | 2.64E-22 | 41.87978 |
| CXCL3 | 1.045514 | 0.100993 | 4.291058 | 10.35232 | 3.28E-23 | 3.02E-22 | 41.74062 |
| GAD1 | 0.706217 | 0.068301 | 1.890177 | 10.3398 | 3.66E-23 | 3.37E-22 | 41.63105 |
| PGC | -2.23191 | 0.21592 | 5.66963 | -10.3367 | 3.76E-23 | 3.46E-22 | 41.60398 |
| LY6H | -0.63741 | 0.06171 | 2.471912 | -10.3292 | 4.02E-23 | 3.69E-22 | 41.53871 |
| VMP1 | 0.605988 | 0.05871 | 4.520521 | 10.32175 | 4.29E-23 | 3.93E-22 | 41.47325 |
| ANG | -0.66989 | 0.064956 | 4.716503 | -10.313 | 4.64E-23 | 4.24E-22 | 41.39634 |
| PEG3 | -0.6453 | 0.062602 | 2.379913 | -10.3081 | 4.85E-23 | 4.42E-22 | 41.35373 |
| FPR2 | 0.616189 | 0.059781 | 2.781763 | 10.30749 | 4.87E-23 | 4.44E-22 | 41.34863 |
| KRT6B | 1.221275 | 0.1185 | 2.833134 | 10.30614 | 4.93E-23 | 4.49E-22 | 41.33689 |
| PMP2 | -0.65702 | 0.063864 | 1.599946 | -10.2877 | 5.80E-23 | 5.25E-22 | 41.1757 |
| PLIN4 | -0.85523 | 0.083184 | 3.567231 | -10.2812 | 6.14E-23 | 5.56E-22 | 41.11913 |
| PIK3C2G | -1.31455 | 0.128004 | 3.237254 | -10.2696 | 6.80E-23 | 6.14E-22 | 41.01843 |
| KLK8 | 0.98414 | 0.096046 | 2.4145 | 10.24659 | 8.33E-23 | 7.44E-22 | 40.81808 |
| SDPR | -0.70229 | 0.068584 | 3.738449 | -10.2397 | 8.85E-23 | 7.89E-22 | 40.75845 |
| PCOLCE2 | -0.8792 | 0.085959 | 2.911949 | -10.2281 | 9.80E-23 | 8.67E-22 | 40.65765 |
| KIF11 | 0.654002 | 0.063954 | 3.726365 | 10.2261 | 9.98E-23 | 8.82E-22 | 40.64008 |
| MUC5AC | -1.3275 | 0.130003 | 4.253854 | -10.2113 | 1.14E-22 | 9.98E-22 | 40.51166 |
| RYR3 | -0.79416 | 0.077787 | 2.172993 | -10.2093 | 1.16E-22 | 1.01E-21 | 40.49456 |
| MACC1 | 0.891694 | 0.087341 | 3.104549 | 10.2093 | 1.16E-22 | 1.01E-21 | 40.4943 |
| RAB9B | -0.6511 | 0.063788 | 3.250939 | -10.2073 | 1.18E-22 | 1.03E-21 | 40.47711 |
| C6orf58 | -1.55329 | 0.152196 | 3.150537 | -10.2058 | 1.19E-22 | 1.04E-21 | 40.46439 |
| HMMR | 0.733057 | 0.071862 | 4.05284 | 10.20091 | 1.24E-22 | 1.09E-21 | 40.42156 |
| CXCL17 | -1.80697 | 0.177222 | 4.217158 | -10.1961 | 1.30E-22 | 1.14E-21 | 40.37979 |
| TNFSF9 | 0.626103 | 0.061495 | 2.477167 | 10.1813 | 1.48E-22 | 1.29E-21 | 40.25175 |
| SGCG | -0.64277 | 0.063219 | 1.859394 | -10.1674 | 1.67E-22 | 1.45E-21 | 40.13172 |
| CD86 | 0.699856 | 0.068908 | 3.558714 | 10.15639 | 1.84E-22 | 1.59E-21 | 40.03631 |
| IDO1 | 0.904492 | 0.089084 | 4.09985 | 10.15327 | 1.89E-22 | 1.63E-21 | 40.00936 |
| VIP | -1.09517 | 0.107872 | 2.406261 | -10.1525 | 1.90E-22 | 1.64E-21 | 40.00293 |
| HOTAIR | 0.623159 | 0.061462 | 1.608057 | 10.13886 | 2.14E-22 | 1.84E-21 | 39.88495 |
| SYNM | -1.37407 | 0.135533 | 4.785602 | -10.1383 | 2.15E-22 | 1.85E-21 | 39.87979 |
| BEX2 | -0.75057 | 0.074113 | 3.579685 | -10.1274 | 2.37E-22 | 2.02E-21 | 39.78624 |
| NBEA | -0.73188 | 0.072515 | 3.512131 | -10.0927 | 3.20E-22 | 2.72E-21 | 39.48759 |
| CLRN3 | 1.378766 | 0.136696 | 4.029446 | 10.08638 | 3.38E-22 | 2.87E-21 | 39.43285 |
| ARHGEF26 | -0.62081 | 0.061561 | 2.770083 | -10.0845 | 3.44E-22 | 2.91E-21 | 39.41656 |
| ADAMTS18 | 0.617579 | 0.061247 | 1.886482 | 10.08343 | 3.47E-22 | 2.93E-21 | 39.40755 |
| CTSG | -0.71054 | 0.070468 | 3.638918 | -10.083 | 3.48E-22 | 2.94E-21 | 39.40396 |
| C5AR1 | 0.670956 | 0.066687 | 3.490669 | 10.06123 | 4.21E-22 | 3.53E-21 | 39.21684 |
| COL2A1 | -1.15289 | 0.114731 | 2.055448 | -10.0486 | 4.70E-22 | 3.92E-21 | 39.10852 |
| MUC6 | -1.30083 | 0.12955 | 3.718147 | -10.0411 | 5.01E-22 | 4.17E-21 | 39.0444 |
| SERPINB2 | 0.619963 | 0.061752 | 1.895677 | 10.03959 | 5.08E-22 | 4.22E-21 | 39.03124 |
| KCNA5 | -0.77496 | 0.077264 | 2.48905 | -10.0301 | 5.52E-22 | 4.58E-21 | 38.95 |
| AQP9 | 0.808373 | 0.080673 | 3.600196 | 10.02033 | 6.00E-22 | 4.97E-21 | 38.86627 |
| RFX6 | -1.1016 | 0.110052 | 2.348569 | -10.0098 | 6.57E-22 | 5.43E-21 | 38.77652 |
| GUCA1C | -0.5944 | 0.059395 | 1.578252 | -10.0076 | 6.70E-22 | 5.53E-21 | 38.75747 |
| PTCHD1 | -0.92559 | 0.092491 | 1.924529 | -10.0074 | 6.72E-22 | 5.54E-21 | 38.75529 |
| COL1A2 | 0.738775 | 0.073866 | 5.953036 | 10.00155 | 7.06E-22 | 5.80E-21 | 38.7057 |
| BEX4 | -0.58564 | 0.058653 | 3.90238 | -9.98489 | 8.16E-22 | 6.68E-21 | 38.56335 |
| GGH | 0.628545 | 0.063071 | 4.581926 | 9.965736 | 9.62E-22 | 7.83E-21 | 38.39996 |
| KIAA0101 | 0.701406 | 0.070434 | 4.409513 | 9.95831 | 1.03E-21 | 8.32E-21 | 38.33667 |
| SLC28A2 | -1.27143 | 0.128019 | 2.812006 | -9.93158 | 1.29E-21 | 1.04E-20 | 38.10914 |
| PDZRN4 | -1.19555 | 0.120485 | 2.613067 | -9.92274 | 1.39E-21 | 1.12E-20 | 38.034 |
| KRT23 | 0.888143 | 0.089524 | 2.488425 | 9.920739 | 1.42E-21 | 1.14E-20 | 38.01698 |
| MSLN | 1.028881 | 0.104237 | 2.935148 | 9.870587 | 2.18E-21 | 1.72E-20 | 37.59159 |
| ONECUT2 | 0.769968 | 0.07806 | 3.029325 | 9.863765 | 2.31E-21 | 1.82E-20 | 37.53384 |
| WIF1 | -0.7586 | 0.076976 | 1.867878 | -9.85499 | 2.49E-21 | 1.96E-20 | 37.4596 |
| MTUS2 | -0.63117 | 0.064116 | 2.128244 | -9.84409 | 2.74E-21 | 2.14E-20 | 37.36745 |
| NEUROD1 | -0.85002 | 0.086391 | 1.88113 | -9.83928 | 2.85E-21 | 2.23E-20 | 37.32685 |
| HOXA11 | 0.609294 | 0.061975 | 2.333921 | 9.8313 | 3.05E-21 | 2.38E-20 | 37.25944 |
| FOXF1 | -0.61817 | 0.062883 | 4.639087 | -9.8305 | 3.07E-21 | 2.40E-20 | 37.2527 |
| BCHE | -1.08427 | 0.110498 | 2.828156 | -9.8126 | 3.58E-21 | 2.77E-20 | 37.10167 |
| MMP1 | 1.192777 | 0.121567 | 5.236547 | 9.811711 | 3.61E-21 | 2.79E-20 | 37.09418 |
| GRP | -0.84591 | 0.086247 | 2.2609 | -9.80809 | 3.72E-21 | 2.88E-20 | 37.06364 |
| GPRASP1 | -0.69203 | 0.070647 | 3.603284 | -9.79569 | 4.14E-21 | 3.19E-20 | 36.95922 |
| ABI3BP | -0.6572 | 0.067132 | 2.744656 | -9.78975 | 4.35E-21 | 3.35E-20 | 36.90915 |
| PLN | -1.1943 | 0.122059 | 3.583389 | -9.78463 | 4.55E-21 | 3.49E-20 | 36.86608 |
| FXYD6 | -0.72117 | 0.0738 | 4.153249 | -9.77199 | 5.06E-21 | 3.87E-20 | 36.75978 |
| CXCL12 | -0.6926 | 0.07092 | 4.187762 | -9.76591 | 5.33E-21 | 4.07E-20 | 36.70867 |
| CXCL5 | 0.915996 | 0.09384 | 3.755153 | 9.761202 | 5.55E-21 | 4.23E-20 | 36.66916 |
| RAB26 | -0.75594 | 0.07767 | 3.267766 | -9.73261 | 7.08E-21 | 5.35E-20 | 36.42924 |
| LINC00261 | -1.51872 | 0.156261 | 3.906389 | -9.71915 | 7.93E-21 | 5.99E-20 | 36.31652 |
| CMA1 | -0.66677 | 0.068655 | 2.418892 | -9.712 | 8.43E-21 | 6.35E-20 | 36.25668 |
| REEP1 | -0.78475 | 0.080915 | 3.630705 | -9.69853 | 9.45E-21 | 7.11E-20 | 36.14398 |
| HOXB7 | 0.637167 | 0.066081 | 3.406271 | 9.642148 | 1.52E-20 | 1.13E-19 | 35.67367 |
| CA9 | -1.40975 | 0.146211 | 3.820478 | -9.64188 | 1.52E-20 | 1.13E-19 | 35.67145 |
| C4BPA | 0.635715 | 0.06596 | 3.008192 | 9.637848 | 1.58E-20 | 1.17E-19 | 35.63788 |
| FER1L4 | -0.64325 | 0.066812 | 3.04569 | -9.62768 | 1.72E-20 | 1.27E-19 | 35.55332 |
| LRRN4CL | -0.64799 | 0.067415 | 3.480696 | -9.61202 | 1.96E-20 | 1.44E-19 | 35.4232 |
| COLEC12 | -0.65398 | 0.068087 | 3.750362 | -9.6051 | 2.08E-20 | 1.53E-19 | 35.36572 |
| DIRAS3 | -0.58707 | 0.061171 | 2.931894 | -9.59715 | 2.22E-20 | 1.63E-19 | 35.29977 |
| KCNB1 | -0.74387 | 0.077706 | 2.378501 | -9.5729 | 2.72E-20 | 1.98E-19 | 35.09877 |
| DPCR1 | -1.76134 | 0.184154 | 4.407256 | -9.5645 | 2.92E-20 | 2.12E-19 | 35.02923 |
| GPT | -0.64554 | 0.067567 | 3.13633 | -9.55404 | 3.19E-20 | 2.30E-19 | 34.94272 |
| COL8A1 | 0.776183 | 0.081318 | 3.071126 | 9.545023 | 3.44E-20 | 2.48E-19 | 34.86815 |
| C8orf12 | -0.59969 | 0.062874 | 1.81211 | -9.53797 | 3.65E-20 | 2.62E-19 | 34.8099 |
| SYNPO2 | -1.0147 | 0.106401 | 4.00504 | -9.53661 | 3.69E-20 | 2.65E-19 | 34.79863 |
| IL33 | -0.71258 | 0.074976 | 4.131481 | -9.50422 | 4.84E-20 | 3.45E-19 | 34.53151 |
| C7 | -0.86753 | 0.091326 | 3.642395 | -9.49929 | 5.04E-20 | 3.59E-19 | 34.49096 |
| CLDN2 | 0.984407 | 0.103788 | 3.172257 | 9.484791 | 5.69E-20 | 4.03E-19 | 34.37161 |
| UCA1 | 0.624558 | 0.065985 | 2.914739 | 9.465186 | 6.69E-20 | 4.72E-19 | 34.21049 |
| MFAP4 | -0.72336 | 0.076456 | 4.738706 | -9.46117 | 6.92E-20 | 4.87E-19 | 34.17754 |
| PRELP | -0.715 | 0.075575 | 3.323986 | -9.46078 | 6.94E-20 | 4.89E-19 | 34.1743 |
| ASF1B | 0.616568 | 0.06522 | 3.828493 | 9.453653 | 7.37E-20 | 5.18E-19 | 34.11583 |
| MT1F | -0.75744 | 0.080272 | 5.203429 | -9.4359 | 8.54E-20 | 5.96E-19 | 33.97023 |
| STK31 | 0.723496 | 0.076895 | 2.641771 | 9.408903 | 1.07E-19 | 7.41E-19 | 33.74932 |
| IGFBP2 | -0.65233 | 0.069349 | 5.233552 | -9.40653 | 1.09E-19 | 7.55E-19 | 33.7299 |
| ZIC2 | 0.652362 | 0.069425 | 1.842971 | 9.396672 | 1.18E-19 | 8.17E-19 | 33.64936 |
| MT1G | -0.94352 | 0.100619 | 4.157956 | -9.37711 | 1.39E-19 | 9.54E-19 | 33.48972 |
| FXYD1 | -0.60794 | 0.064861 | 3.547598 | -9.37291 | 1.44E-19 | 9.86E-19 | 33.45546 |
| AGT | 0.754282 | 0.080478 | 4.465833 | 9.372535 | 1.44E-19 | 9.89E-19 | 33.4524 |
| CNTN1 | -0.63235 | 0.067497 | 1.970861 | -9.36864 | 1.49E-19 | 1.02E-18 | 33.42069 |
| LMOD1 | -0.97706 | 0.104406 | 3.704605 | -9.35835 | 1.62E-19 | 1.11E-18 | 33.33681 |
| AOC3 | -0.82668 | 0.088352 | 4.46154 | -9.35671 | 1.64E-19 | 1.12E-18 | 33.32344 |
| MGAM | -0.74168 | 0.079422 | 2.593073 | -9.33852 | 1.91E-19 | 1.29E-18 | 33.17548 |
| ADH1C | -1.18342 | 0.126896 | 5.423873 | -9.32597 | 2.12E-19 | 1.43E-18 | 33.07346 |
| ASB5 | -1.25128 | 0.134248 | 2.071879 | -9.32069 | 2.21E-19 | 1.49E-18 | 33.03064 |
| PI3 | 1.05998 | 0.113876 | 4.257293 | 9.308171 | 2.45E-19 | 1.65E-18 | 32.92902 |
| CDX2 | 0.95713 | 0.102867 | 2.917114 | 9.304498 | 2.53E-19 | 1.70E-18 | 32.89923 |
| VSIG1 | -1.56977 | 0.168957 | 4.053888 | -9.29093 | 2.83E-19 | 1.89E-18 | 32.78925 |
| HPN | -0.92726 | 0.100126 | 2.970903 | -9.26092 | 3.61E-19 | 2.40E-18 | 32.54652 |
| C2CD4A | 0.837921 | 0.090691 | 3.123078 | 9.239347 | 4.31E-19 | 2.83E-18 | 32.37235 |
| LTF | -1.4679 | 0.158889 | 4.807597 | -9.23851 | 4.34E-19 | 2.85E-18 | 32.3656 |
| FAM129A | -0.69718 | 0.075482 | 4.923567 | -9.23636 | 4.42E-19 | 2.90E-18 | 32.34826 |
| TNS4 | 0.683959 | 0.074155 | 3.258064 | 9.223416 | 4.91E-19 | 3.21E-18 | 32.24394 |
| OGN | -1.23438 | 0.134077 | 3.895145 | -9.20646 | 5.64E-19 | 3.67E-18 | 32.10747 |
| SCIN | -0.75159 | 0.082042 | 3.24735 | -9.16106 | 8.16E-19 | 5.25E-18 | 31.74291 |
| KCNA1 | -0.61196 | 0.066802 | 2.012991 | -9.16069 | 8.19E-19 | 5.27E-18 | 31.73997 |
| HYAL1 | -0.66037 | 0.072109 | 3.728158 | -9.15796 | 8.37E-19 | 5.38E-18 | 31.71808 |
| HOXB13 | 0.83315 | 0.091067 | 2.468065 | 9.148767 | 9.02E-19 | 5.78E-18 | 31.64444 |
| RNF150 | -0.67591 | 0.074218 | 2.921929 | -9.10705 | 1.26E-18 | 8.02E-18 | 31.31102 |
| KRT20 | -1.66387 | 0.183434 | 4.360765 | -9.07065 | 1.70E-18 | 1.06E-17 | 31.02097 |
| BEX1 | -0.84527 | 0.093538 | 3.63916 | -9.03657 | 2.23E-18 | 1.39E-17 | 30.75032 |
| ARSE | 0.614485 | 0.068231 | 3.154567 | 9.005955 | 2.86E-18 | 1.76E-17 | 30.50777 |
| MYOCD | -0.81125 | 0.090092 | 3.398984 | -9.00468 | 2.88E-18 | 1.78E-17 | 30.49767 |
| KRT18 | 0.783624 | 0.087041 | 6.343283 | 9.002914 | 2.93E-18 | 1.81E-17 | 30.48372 |
| ACTG2 | -0.83946 | 0.093314 | 4.493358 | -8.99606 | 3.09E-18 | 1.90E-17 | 30.42949 |
| AGTR1 | -0.78851 | 0.087761 | 2.425213 | -8.98479 | 3.38E-18 | 2.07E-17 | 30.34046 |
| CPB1 | -0.75248 | 0.083752 | 2.034247 | -8.98457 | 3.39E-18 | 2.08E-17 | 30.33872 |
| MT1H | -0.71858 | 0.079988 | 5.486134 | -8.98359 | 3.42E-18 | 2.09E-17 | 30.33097 |
| LIMS2 | -0.6787 | 0.075609 | 3.921446 | -8.97647 | 3.62E-18 | 2.21E-17 | 30.27477 |
| KCNMA1 | -0.71067 | 0.079234 | 2.725132 | -8.96927 | 3.83E-18 | 2.33E-17 | 30.21801 |
| ASCL2 | 0.605174 | 0.067678 | 2.931703 | 8.942021 | 4.76E-18 | 2.88E-17 | 30.00331 |
| HRASLS2 | -0.88456 | 0.098978 | 3.157259 | -8.93688 | 4.96E-18 | 3.00E-17 | 29.96287 |
| MORN5 | -1.04401 | 0.117083 | 2.47829 | -8.91687 | 5.82E-18 | 3.50E-17 | 29.80561 |
| PDZD3 | -0.60544 | 0.067936 | 3.035669 | -8.91189 | 6.06E-18 | 3.64E-17 | 29.76655 |
| BCAS1 | -1.09318 | 0.122671 | 4.115686 | -8.9115 | 6.08E-18 | 3.65E-17 | 29.76348 |
| TMPRSS4 | 0.869872 | 0.097923 | 4.121232 | 8.883193 | 7.61E-18 | 4.53E-17 | 29.54157 |
| FMO5 | -0.71637 | 0.080678 | 3.226946 | -8.87933 | 7.85E-18 | 4.67E-17 | 29.51131 |
| TNFRSF17 | -1.08248 | 0.121987 | 3.397259 | -8.87369 | 8.21E-18 | 4.88E-17 | 29.46719 |
| CTNNA3 | -0.61289 | 0.069192 | 1.727729 | -8.85778 | 9.31E-18 | 5.51E-17 | 29.3428 |
| CFL2 | -0.71882 | 0.081279 | 4.090925 | -8.84393 | 1.04E-17 | 6.13E-17 | 29.23467 |
| SULT1B1 | -0.83436 | 0.094421 | 3.334443 | -8.83655 | 1.10E-17 | 6.48E-17 | 29.17711 |
| COL6A5 | -0.62244 | 0.070449 | 1.784368 | -8.83532 | 1.11E-17 | 6.53E-17 | 29.16755 |
| SOX2 | -0.60513 | 0.068644 | 2.901665 | -8.81542 | 1.30E-17 | 7.60E-17 | 29.01251 |
| FCGBP | -1.25771 | 0.142724 | 4.7772 | -8.81218 | 1.34E-17 | 7.79E-17 | 28.98728 |
| CIDEA | -0.608 | 0.069153 | 2.530794 | -8.79212 | 1.56E-17 | 9.07E-17 | 28.83134 |
| FNDC1 | 1.065984 | 0.12136 | 3.208559 | 8.783654 | 1.67E-17 | 9.66E-17 | 28.7656 |
| CCL18 | 0.858424 | 0.09785 | 4.514997 | 8.772824 | 1.82E-17 | 1.05E-16 | 28.68158 |
| GJB5 | 0.595179 | 0.06803 | 2.023195 | 8.748789 | 2.20E-17 | 1.26E-16 | 28.49539 |
| CEACAM6 | 1.450325 | 0.166048 | 5.061166 | 8.734397 | 2.46E-17 | 1.41E-16 | 28.3841 |
| HPDL | 0.58826 | 0.067371 | 3.266962 | 8.731669 | 2.52E-17 | 1.43E-16 | 28.36302 |
| CLIC6 | -0.96569 | 0.110773 | 3.693952 | -8.7178 | 2.80E-17 | 1.59E-16 | 28.25594 |
| SOX9 | 0.728218 | 0.083581 | 4.495873 | 8.712675 | 2.92E-17 | 1.65E-16 | 28.21639 |
| S100A9 | 0.721823 | 0.082848 | 4.602701 | 8.712613 | 2.92E-17 | 1.65E-16 | 28.21591 |
| SULT1C2 | -1.1136 | 0.128023 | 4.357323 | -8.69844 | 3.26E-17 | 1.84E-16 | 28.10668 |
| HAPLN1 | -0.74731 | 0.086184 | 2.371696 | -8.6711 | 4.04E-17 | 2.26E-16 | 27.89633 |
| HAND2 | -0.65636 | 0.075791 | 2.70187 | -8.6601 | 4.40E-17 | 2.46E-16 | 27.81178 |
| TNFAIP6 | 0.647703 | 0.07496 | 3.827506 | 8.640687 | 5.12E-17 | 2.84E-16 | 27.6629 |
| TGFB1I1 | -0.61269 | 0.070998 | 5.161743 | -8.62974 | 5.58E-17 | 3.08E-16 | 27.57908 |
| CSTA | -0.64263 | 0.074528 | 4.542551 | -8.62277 | 5.89E-17 | 3.25E-16 | 27.52568 |
| EPCAM | 0.979662 | 0.113905 | 6.33351 | 8.600708 | 6.98E-17 | 3.83E-16 | 27.35705 |
| CCL21 | -0.67018 | 0.077948 | 3.959454 | -8.59777 | 7.15E-17 | 3.91E-16 | 27.3346 |
| ALDH1A1 | -0.69385 | 0.080715 | 5.551711 | -8.59626 | 7.23E-17 | 3.95E-16 | 27.32306 |
| RGMA | -0.6049 | 0.070398 | 3.320677 | -8.5926 | 7.44E-17 | 4.06E-16 | 27.29519 |
| MAGEA6 | 1.281274 | 0.149451 | 2.574158 | 8.573193 | 8.64E-17 | 4.70E-16 | 27.1472 |
| TAC1 | -0.90275 | 0.105307 | 2.084313 | -8.57259 | 8.68E-17 | 4.72E-16 | 27.14259 |
| CRYAB | -0.76426 | 0.089329 | 4.385655 | -8.55564 | 9.90E-17 | 5.35E-16 | 27.01358 |
| CNN1 | -1.09447 | 0.12802 | 5.204 | -8.54919 | 1.04E-16 | 5.62E-16 | 26.96454 |
| CYS1 | -0.60757 | 0.071177 | 3.172275 | -8.53613 | 1.15E-16 | 6.19E-16 | 26.86536 |
| AKR1B10 | -1.52687 | 0.179125 | 5.450626 | -8.52408 | 1.26E-16 | 6.76E-16 | 26.77392 |
| RBM24 | -0.75458 | 0.088663 | 2.423158 | -8.51067 | 1.40E-16 | 7.47E-16 | 26.6723 |
| SMYD1 | -1.02599 | 0.120705 | 2.459246 | -8.49995 | 1.52E-16 | 8.10E-16 | 26.59118 |
| TFF2 | -1.65974 | 0.195333 | 5.929394 | -8.49696 | 1.56E-16 | 8.27E-16 | 26.56851 |
| CDH17 | 1.228416 | 0.145 | 3.996647 | 8.471833 | 1.89E-16 | 9.97E-16 | 26.37868 |
| SGK2 | -0.67823 | 0.080211 | 3.374684 | -8.45549 | 2.14E-16 | 1.13E-15 | 26.25546 |
| RERG | -0.76084 | 0.09057 | 3.588375 | -8.4006 | 3.25E-16 | 1.69E-15 | 25.84289 |
| CCL20 | 1.149009 | 0.136831 | 4.391101 | 8.397265 | 3.33E-16 | 1.73E-15 | 25.81788 |
| PLA2G2A | 1.119555 | 0.133452 | 4.228672 | 8.3892 | 3.55E-16 | 1.84E-15 | 25.75746 |
| ASB2 | -0.63773 | 0.076155 | 3.491308 | -8.37413 | 3.98E-16 | 2.06E-15 | 25.6447 |
| THBS2 | 0.950516 | 0.113589 | 4.247684 | 8.368002 | 4.17E-16 | 2.15E-15 | 25.59887 |
| LAD1 | 0.808137 | 0.096808 | 4.013924 | 8.347863 | 4.85E-16 | 2.49E-15 | 25.4485 |
| ANGPTL7 | -0.71174 | 0.085396 | 2.357972 | -8.33462 | 5.36E-16 | 2.74E-15 | 25.34974 |
| EML1 | -0.59574 | 0.071608 | 3.595966 | -8.31948 | 6.02E-16 | 3.06E-15 | 25.23705 |
| BARX1 | -0.69201 | 0.083301 | 3.289257 | -8.30741 | 6.59E-16 | 3.35E-15 | 25.14733 |
| TCEAL7 | -0.61686 | 0.074503 | 3.129591 | -8.27969 | 8.12E-16 | 4.10E-15 | 24.9416 |
| CYP2C19 | -0.7557 | 0.091351 | 2.534509 | -8.27248 | 8.58E-16 | 4.33E-15 | 24.88818 |
| PRRX1 | 0.66662 | 0.080652 | 3.390479 | 8.26542 | 9.04E-16 | 4.55E-15 | 24.83591 |
| PPP1R1B | 0.793922 | 0.096122 | 3.739137 | 8.259509 | 9.45E-16 | 4.75E-15 | 24.79217 |
| LYPD6B | -0.98723 | 0.11986 | 3.536702 | -8.23656 | 1.12E-15 | 5.59E-15 | 24.62263 |
| IGJ | -0.99889 | 0.121548 | 6.095069 | -8.21805 | 1.29E-15 | 6.40E-15 | 24.48612 |
| KLK7 | 0.723757 | 0.088519 | 2.823843 | 8.176273 | 1.76E-15 | 8.68E-15 | 24.17893 |
| DES | -0.79669 | 0.097693 | 3.822227 | -8.15505 | 2.07E-15 | 1.01E-14 | 24.02339 |
| HHIP | -0.60927 | 0.074979 | 2.548468 | -8.12585 | 2.57E-15 | 1.25E-14 | 23.80983 |
| ITPKA | -0.67073 | 0.083311 | 3.512398 | -8.05093 | 4.47E-15 | 2.14E-14 | 23.26487 |
| NEXN | -0.73761 | 0.091644 | 4.297182 | -8.04867 | 4.54E-15 | 2.17E-14 | 23.2485 |
| SMPX | -1.20958 | 0.150779 | 2.665619 | -8.02225 | 5.52E-15 | 2.62E-14 | 23.05728 |
| MUC13 | 0.934002 | 0.117266 | 4.218626 | 7.964789 | 8.41E-15 | 3.93E-14 | 22.64324 |
| AADAC | -1.10114 | 0.138733 | 4.059873 | -7.93711 | 1.03E-14 | 4.78E-14 | 22.44462 |
| CYP4X1 | -0.6817 | 0.086238 | 3.414761 | -7.90486 | 1.30E-14 | 6.01E-14 | 22.21398 |
| MAGEA12 | 1.013554 | 0.128987 | 2.101352 | 7.857779 | 1.83E-14 | 8.36E-14 | 21.87855 |
| CA2 | -1.10451 | 0.14065 | 5.837948 | -7.8529 | 1.90E-14 | 8.64E-14 | 21.84388 |
| HSPB6 | -0.78633 | 0.100412 | 3.076709 | -7.83102 | 2.22E-14 | 1.01E-13 | 21.68862 |
| VIL1 | 0.866114 | 0.111157 | 3.577242 | 7.791839 | 2.94E-14 | 1.32E-13 | 21.41153 |
| MYLK | -0.64934 | 0.083549 | 4.224373 | -7.77206 | 3.39E-14 | 1.52E-13 | 21.27205 |
| ZFPM2 | -0.66221 | 0.085553 | 3.080381 | -7.74036 | 4.26E-14 | 1.89E-13 | 21.04918 |
| PKP3 | 0.68894 | 0.089592 | 4.057766 | 7.689754 | 6.11E-14 | 2.68E-13 | 20.69485 |
| CEACAM7 | 0.702123 | 0.091423 | 2.650289 | 7.679905 | 6.55E-14 | 2.87E-13 | 20.62612 |
| PCSK9 | 0.616511 | 0.080414 | 2.980326 | 7.666731 | 7.19E-14 | 3.13E-13 | 20.53429 |
| PENK | -0.70906 | 0.092506 | 2.405115 | -7.66501 | 7.28E-14 | 3.17E-13 | 20.52227 |
| CXCL11 | 0.815956 | 0.106683 | 3.222652 | 7.648423 | 8.19E-14 | 3.55E-13 | 20.40689 |
| CYP2C9 | -0.83389 | 0.109056 | 3.216547 | -7.64641 | 8.31E-14 | 3.60E-13 | 20.39287 |
| SMTN | -0.61164 | 0.080173 | 3.914528 | -7.62895 | 9.40E-14 | 4.04E-13 | 20.27166 |
| PROK2 | 0.587005 | 0.077111 | 2.135462 | 7.61244 | 1.06E-13 | 4.52E-13 | 20.15724 |
| SLCO1B3 | 0.687241 | 0.090321 | 2.031787 | 7.608893 | 1.08E-13 | 4.63E-13 | 20.13268 |
| THOC6 | 0.644099 | 0.084677 | 3.565754 | 7.606515 | 1.10E-13 | 4.71E-13 | 20.11621 |
| ANXA13 | 0.900332 | 0.118405 | 3.674397 | 7.603843 | 1.12E-13 | 4.79E-13 | 20.09772 |
| RORC | -0.6599 | 0.086803 | 3.340628 | -7.60234 | 1.13E-13 | 4.84E-13 | 20.08732 |
| FGA | -0.68825 | 0.090856 | 2.116626 | -7.57523 | 1.37E-13 | 5.82E-13 | 19.9001 |
| FAM3B | -1.18346 | 0.156736 | 4.75903 | -7.55064 | 1.63E-13 | 6.88E-13 | 19.73073 |
| EHF | 0.809504 | 0.107825 | 3.653515 | 7.5076 | 2.20E-13 | 9.18E-13 | 19.43535 |
| PP7080 | -0.6658 | 0.089229 | 3.767017 | -7.46162 | 3.03E-13 | 1.25E-12 | 19.12133 |
| GGT6 | -0.5897 | 0.079199 | 3.364248 | -7.44577 | 3.39E-13 | 1.39E-12 | 19.01348 |
| REG3A | -1.20135 | 0.161418 | 3.67368 | -7.44245 | 3.47E-13 | 1.42E-12 | 18.99095 |
| CSRP1 | -0.59021 | 0.080201 | 5.187291 | -7.35916 | 6.16E-13 | 2.48E-12 | 18.42746 |
| CYP2C18 | -1.0419 | 0.141723 | 3.815563 | -7.35168 | 6.48E-13 | 2.60E-12 | 18.37711 |
| SLC9A2 | -0.63162 | 0.086088 | 2.271363 | -7.33686 | 7.17E-13 | 2.87E-12 | 18.27747 |
| RASSF6 | -0.63954 | 0.087241 | 3.958322 | -7.33067 | 7.48E-13 | 2.99E-12 | 18.23593 |
| DUOX1 | -0.61991 | 0.084842 | 2.470892 | -7.30665 | 8.82E-13 | 3.50E-12 | 18.07489 |
| MRGPRF | -0.64813 | 0.088755 | 3.886401 | -7.30245 | 9.08E-13 | 3.60E-12 | 18.0468 |
| MLK7-AS1 | 0.689876 | 0.094747 | 2.249696 | 7.281281 | 1.05E-12 | 4.14E-12 | 17.90535 |
| VILL | -0.60288 | 0.082813 | 4.449118 | -7.28003 | 1.06E-12 | 4.17E-12 | 17.89702 |
| B3GNT6 | -0.6497 | 0.089361 | 2.792755 | -7.27054 | 1.13E-12 | 4.43E-12 | 17.83369 |
| CAPN8 | -1.02185 | 0.140744 | 4.25436 | -7.26031 | 1.21E-12 | 4.74E-12 | 17.76555 |
| PRSS3 | 0.675483 | 0.093633 | 4.61406 | 7.214141 | 1.65E-12 | 6.41E-12 | 17.45898 |
| MIR100HG | -0.72855 | 0.103309 | 3.648422 | -7.05219 | 4.89E-12 | 1.82E-11 | 16.39663 |
| SFRP4 | 0.923124 | 0.13146 | 3.248289 | 7.022092 | 5.97E-12 | 2.21E-11 | 16.20141 |
| MYL9 | -0.64802 | 0.092333 | 4.373594 | -7.01831 | 6.12E-12 | 2.26E-11 | 16.17693 |
| MRAP2 | -0.59284 | 0.084708 | 3.662806 | -6.99855 | 6.97E-12 | 2.57E-11 | 16.04921 |
| A4GNT | -0.78623 | 0.112343 | 2.877785 | -6.99849 | 6.98E-12 | 2.57E-11 | 16.04881 |
| FLNC | -0.64887 | 0.093074 | 3.925494 | -6.97148 | 8.34E-12 | 3.05E-11 | 15.87479 |
| AZGP1 | -0.83829 | 0.120284 | 3.665979 | -6.96919 | 8.46E-12 | 3.09E-11 | 15.86006 |
| EPN3 | -0.78695 | 0.113056 | 3.511279 | -6.96071 | 8.95E-12 | 3.27E-11 | 15.80549 |
| AXDND1 | -0.70245 | 0.101192 | 2.188438 | -6.94175 | 1.01E-11 | 3.68E-11 | 15.68381 |
| RBFOX3 | -0.85611 | 0.123539 | 2.181085 | -6.92991 | 1.09E-11 | 3.97E-11 | 15.60796 |
| AKR1C3 | -0.6267 | 0.090595 | 5.976555 | -6.9176 | 1.19E-11 | 4.29E-11 | 15.52927 |
| TFCP2L1 | -0.67865 | 0.098127 | 3.509143 | -6.91602 | 1.20E-11 | 4.33E-11 | 15.51917 |
| PRUNE2 | -0.6991 | 0.101104 | 3.43635 | -6.91464 | 1.21E-11 | 4.37E-11 | 15.51034 |
| COL21A1 | -0.63254 | 0.091697 | 3.365509 | -6.89812 | 1.35E-11 | 4.85E-11 | 15.40486 |
| TFF1 | -1.18923 | 0.172567 | 6.391863 | -6.89145 | 1.41E-11 | 5.05E-11 | 15.36239 |
| FILIP1 | -0.61195 | 0.089038 | 2.788512 | -6.87294 | 1.59E-11 | 5.67E-11 | 15.24457 |
| KLK11 | -0.89647 | 0.132235 | 3.823375 | -6.77936 | 2.91E-11 | 1.02E-10 | 14.65319 |
| LRRC66 | -0.68529 | 0.101352 | 3.2167 | -6.76148 | 3.26E-11 | 1.14E-10 | 14.54098 |
| SLC27A2 | 0.602177 | 0.089073 | 3.4395 | 6.760482 | 3.28E-11 | 1.15E-10 | 14.53473 |
| ANXA10 | -1.33577 | 0.197843 | 5.317551 | -6.75167 | 3.47E-11 | 1.21E-10 | 14.47951 |
| OLFM4 | 1.403095 | 0.208687 | 5.002793 | 6.723441 | 4.16E-11 | 1.44E-10 | 14.30312 |
| TRIM31 | 0.79503 | 0.11923 | 3.699724 | 6.668062 | 5.91E-11 | 2.02E-10 | 13.95889 |
| FOLR1 | -0.71323 | 0.107065 | 3.506649 | -6.66168 | 6.16E-11 | 2.10E-10 | 13.91939 |
| COMP | 0.66626 | 0.100636 | 3.07768 | 6.620473 | 7.99E-11 | 2.70E-10 | 13.66504 |
| PDZK1IP1 | 0.779472 | 0.117761 | 4.552485 | 6.61911 | 8.06E-11 | 2.73E-10 | 13.65665 |
| NRXN3 | -0.6233 | 0.094452 | 2.840166 | -6.59915 | 9.13E-11 | 3.08E-10 | 13.53396 |
| OMD | -0.63117 | 0.096196 | 2.669118 | -6.56133 | 1.16E-10 | 3.87E-10 | 13.30239 |
| NMU | 0.774552 | 0.118624 | 3.87559 | 6.529485 | 1.41E-10 | 4.68E-10 | 13.10827 |
| DSG3 | 0.639283 | 0.098973 | 2.261329 | 6.459173 | 2.19E-10 | 7.15E-10 | 12.68259 |
| PHGR1 | -0.65344 | 0.101824 | 3.127156 | -6.41732 | 2.83E-10 | 9.15E-10 | 12.43109 |
| CFTR | 0.632867 | 0.098651 | 2.448915 | 6.415238 | 2.86E-10 | 9.26E-10 | 12.41864 |
| HSPB3 | -0.59671 | 0.094097 | 2.291875 | -6.34143 | 4.50E-10 | 1.43E-09 | 11.97872 |
| TAGLN | -0.61181 | 0.096798 | 5.383419 | -6.32045 | 5.11E-10 | 1.61E-09 | 11.85455 |
| IL1R2 | -0.69184 | 0.109966 | 4.141383 | -6.29138 | 6.09E-10 | 1.91E-09 | 11.68296 |
| SELE | 0.600693 | 0.095596 | 2.620069 | 6.283666 | 6.38E-10 | 2.00E-09 | 11.63758 |
| ODAM | -0.66325 | 0.106499 | 3.180264 | -6.22779 | 8.93E-10 | 2.77E-09 | 11.31014 |
| KIAA1324 | -0.61167 | 0.098598 | 3.762566 | -6.20361 | 1.03E-09 | 3.18E-09 | 11.16927 |
| NR0B2 | -0.62152 | 0.100614 | 3.665275 | -6.17727 | 1.21E-09 | 3.70E-09 | 11.01635 |
| CILP | -0.63588 | 0.10343 | 4.012458 | -6.14794 | 1.44E-09 | 4.37E-09 | 10.84674 |
| MUC17 | 0.703242 | 0.114401 | 2.993201 | 6.147156 | 1.44E-09 | 4.39E-09 | 10.84221 |
| C1orf106 | 0.615928 | 0.101464 | 4.711585 | 6.070423 | 2.27E-09 | 6.79E-09 | 10.40187 |
| PNLIPRP2 | -0.67776 | 0.111696 | 2.296076 | -6.0679 | 2.30E-09 | 6.88E-09 | 10.38747 |
| CAP2 | -0.59255 | 0.098169 | 3.358382 | -6.03599 | 2.78E-09 | 8.23E-09 | 10.20585 |
| UPK1B | -0.75447 | 0.125105 | 2.965987 | -6.03071 | 2.86E-09 | 8.48E-09 | 10.17592 |
| DMBT1 | 0.887531 | 0.148102 | 4.56267 | 5.992706 | 3.57E-09 | 1.05E-08 | 9.960881 |
| TTR | -0.63313 | 0.106147 | 3.515828 | -5.96462 | 4.20E-09 | 1.23E-08 | 9.802735 |
| GAST | -0.81159 | 0.140046 | 3.480349 | -5.79515 | 1.11E-08 | 3.13E-08 | 8.862604 |
| DKK1 | 0.726731 | 0.126949 | 2.849109 | 5.72459 | 1.64E-08 | 4.59E-08 | 8.478317 |
| FGG | -0.77014 | 0.139072 | 2.193899 | -5.53772 | 4.60E-08 | 1.23E-07 | 7.480979 |
| APOBEC1 | -0.65999 | 0.120284 | 3.039946 | -5.4869 | 6.05E-08 | 1.61E-07 | 7.2149 |
| APOD | -0.60631 | 0.111431 | 4.147265 | -5.44112 | 7.74E-08 | 2.04E-07 | 6.977099 |
| GUCY2C | 0.621274 | 0.115182 | 3.208964 | 5.393855 | 9.95E-08 | 2.60E-07 | 6.733506 |
| GPR128 | 0.642037 | 0.119175 | 2.784283 | 5.387353 | 1.03E-07 | 2.69E-07 | 6.700143 |
| GCG | -0.61229 | 0.114294 | 2.175716 | -5.35715 | 1.21E-07 | 3.14E-07 | 6.545644 |
| MSMB | -1.13926 | 0.213643 | 3.797857 | -5.33257 | 1.38E-07 | 3.55E-07 | 6.420499 |
| REG1A | -1.09177 | 0.207816 | 5.676985 | -5.25353 | 2.08E-07 | 5.28E-07 | 6.021647 |
| TMC5 | 0.676302 | 0.128903 | 4.522444 | 5.246578 | 2.16E-07 | 5.47E-07 | 5.986813 |
| TMEM45B | 0.634699 | 0.121466 | 4.741187 | 5.225337 | 2.41E-07 | 6.08E-07 | 5.880673 |
| ELF3 | 0.613917 | 0.117496 | 4.690487 | 5.224999 | 2.41E-07 | 6.08E-07 | 5.87899 |
| SLC6A14 | 0.787742 | 0.151476 | 3.557864 | 5.200443 | 2.74E-07 | 6.86E-07 | 5.756783 |
| CTSE | -0.81315 | 0.156553 | 5.826349 | -5.19412 | 2.83E-07 | 7.08E-07 | 5.725385 |
| CXCL13 | 0.668165 | 0.129585 | 3.906197 | 5.156177 | 3.43E-07 | 8.53E-07 | 5.537814 |
| MTTP | -0.68627 | 0.133685 | 2.499472 | -5.13351 | 3.85E-07 | 9.52E-07 | 5.426373 |
| CLDN18 | -0.92548 | 0.180391 | 5.23884 | -5.1304 | 3.92E-07 | 9.67E-07 | 5.411089 |
| DEFA5 | -0.70135 | 0.139854 | 2.409717 | -5.01488 | 7.01E-07 | 1.69E-06 | 4.850347 |
| REG4 | 0.807245 | 0.162036 | 3.823326 | 4.981874 | 8.26E-07 | 1.97E-06 | 4.692288 |
| PIGR | -0.836 | 0.172363 | 5.39884 | -4.8502 | 1.58E-06 | 3.65E-06 | 4.071308 |
| TCN1 | -0.75776 | 0.163076 | 4.485853 | -4.64666 | 4.15E-06 | 9.25E-06 | 3.141664 |
| ARL14 | -0.69276 | 0.151298 | 4.435172 | -4.5788 | 5.69E-06 | 1.25E-05 | 2.839994 |
| LCN2 | 0.76787 | 0.168839 | 5.547558 | 4.547934 | 6.56E-06 | 1.43E-05 | 2.704113 |
| TM4SF20 | 0.608828 | 0.134931 | 3.102431 | 4.512147 | 7.73E-06 | 1.67E-05 | 2.547663 |
| SPINK4 | 0.758589 | 0.174241 | 3.919465 | 4.353672 | 1.58E-05 | 3.29E-05 | 1.868774 |
| HEPACAM2 | -0.59034 | 0.138632 | 2.985352 | -4.25836 | 2.39E-05 | 4.91E-05 | 1.471432 |
