# Supplementary material for: Screening of core targets for Di(2-ethylhexyl) Phthalate-related gastric cancer based on machine learning, molecular docking, and SHAP analysis
Source: PLoS Comput Biol. 2026 Jul 16;22(7):e1014514. doi: 10.1371/journal.pcbi.1014514 (PMC13374966; doi:10.1371/journal.pcbi.1014514)
Supplement: S2 Table — (DOCX) [file pcbi.1014514.s003.docx]

**S2** **Table. Performance of the 127 constructed prediction models**

| **Method** | **Train** | **GSE13911** | **GSE19826** | **GSE51575** |
| --- | --- | --- | --- | --- |
| Lasso+Stepglm[both] | 0.975921746 | 0.937181664 | 0.9 | 0.983727811 |
| SVM | 0.933039732 | 0.793293718 | 0.833333333 | 0.865384615 |
| glmBoost+SVM | 0.922107142 | 0.761035654 | 0.7 | 0.826923077 |
| Ridge | 0.971215273 | 0.973684211 | 0.905555556 | 0.99852071 |
| Lasso+SVM | 0.933039732 | 0.793293718 | 0.833333333 | 0.865384615 |
| glmBoost+Ridge | 0.970507089 | 0.971137521 | 0.894444444 | 0.99852071 |
| Enet[alpha=0.1] | 0.977043037 | 0.956706282 | 0.905555556 | 0.99260355 |
| glmBoost+Enet[alpha=0.1] | 0.974770947 | 0.957555178 | 0.9 | 0.99112426 |
| Enet[alpha=0.2] | 0.976865991 | 0.957555178 | 0.9 | 0.99260355 |
| Enet[alpha=0.3] | 0.976984022 | 0.957555178 | 0.9 | 0.99260355 |
| glmBoost+Enet[alpha=0.3] | 0.974593901 | 0.96179966 | 0.9 | 0.99260355 |
| glmBoost+Enet[alpha=0.2] | 0.97466767 | 0.960101868 | 0.9 | 0.99260355 |
| Enet[alpha=0.4] | 0.976393868 | 0.96179966 | 0.9 | 0.99556213 |
| glmBoost+Enet[alpha=0.4] | 0.974534885 | 0.963497453 | 0.905555556 | 0.99260355 |
| Lasso+glmBoost | 0.969120229 | 0.968590832 | 0.888888889 | 0.99852071 |
| Enet[alpha=0.5] | 0.976364361 | 0.960101868 | 0.894444444 | 0.99408284 |
| glmBoost | 0.968751383 | 0.967741935 | 0.888888889 | 0.99852071 |
| glmBoost+Enet[alpha=0.5] | 0.974579147 | 0.962648557 | 0.905555556 | 0.99260355 |
| Enet[alpha=0.6] | 0.976570914 | 0.960101868 | 0.9 | 0.99260355 |
| glmBoost+Enet[alpha=0.6] | 0.974608655 | 0.960101868 | 0.9 | 0.99260355 |
| glmBoost+Enet[alpha=0.7] | 0.974726685 | 0.960950764 | 0.905555556 | 0.99260355 |
| glmBoost+Enet[alpha=0.8] | 0.974874224 | 0.9524618 | 0.9 | 0.98816568 |
| Enet[alpha=0.8] | 0.976615176 | 0.960101868 | 0.894444444 | 0.98964497 |
| Enet[alpha=0.9] | 0.976423376 | 0.960101868 | 0.894444444 | 0.99112426 |
| Lasso | 0.97674796 | 0.956706282 | 0.894444444 | 0.98816568 |
| Enet[alpha=0.7] | 0.976423376 | 0.960950764 | 0.894444444 | 0.99408284 |
| glmBoost+Enet[alpha=0.9] | 0.974815208 | 0.955857385 | 0.9 | 0.99112426 |
| glmBoost+Lasso | 0.974829962 | 0.955008489 | 0.9 | 0.98816568 |
| Lasso+plsRglm | 0.971333304 | 0.981324278 | 0.888888889 | 0.99408284 |
| glmBoost+plsRglm | 0.971790673 | 0.977928693 | 0.894444444 | 0.99704142 |
| glmBoost+Stepglm[forward] | 0.974947993 | 0.950764007 | 0.9 | 0.986686391 |
| Lasso+Stepglm[forward] | 0.977485652 | 0.936332767 | 0.911111111 | 0.985207101 |
| RF+SVM | 0.923235811 | 0.825551783 | 0.766666667 | 0.846153846 |
| Stepglm[forward] | 0.977957775 | 0.932937182 | 0.916666667 | 0.985207101 |
| plsRglm | 0.969208752 | 0.979626486 | 0.888888889 | 0.99704142 |
| RF+Ridge | 0.970167751 | 0.971986418 | 0.894444444 | 0.99852071 |
| RF+Enet[alpha=0.1] | 0.975508638 | 0.955857385 | 0.9 | 0.99112426 |
| RF+plsRglm | 0.97182018 | 0.975382003 | 0.894444444 | 0.99556213 |
| RF+Stepglm[forward] | 0.976128299 | 0.943123939 | 0.905555556 | 0.982248521 |
| RF+Enet[alpha=0.2] | 0.975523392 | 0.956706282 | 0.9 | 0.99112426 |
| RF+Enet[alpha=0.3] | 0.975375854 | 0.957555178 | 0.9 | 0.99112426 |
| RF+Enet[alpha=0.6] | 0.975479131 | 0.956706282 | 0.9 | 0.98964497 |
| RF+Lasso | 0.9757447 | 0.955857385 | 0.9 | 0.98816568 |
| RF+Enet[alpha=0.7] | 0.975154546 | 0.957555178 | 0.9 | 0.98964497 |
| RF+Enet[alpha=0.5] | 0.9755529 | 0.954159593 | 0.9 | 0.98964497 |
| RF+glmBoost | 0.96854483 | 0.966893039 | 0.894444444 | 0.99852071 |
| RF+Enet[alpha=0.9] | 0.975508638 | 0.956706282 | 0.9 | 0.98964497 |
| RF+Enet[alpha=0.4] | 0.975523392 | 0.955008489 | 0.9 | 0.98964497 |
| RF+Enet[alpha=0.8] | 0.975493885 | 0.955857385 | 0.9 | 0.98964497 |
| RF+Stepglm[both] | 0.975125039 | 0.953310696 | 0.9 | 0.98816568 |
| RF+Stepglm[backward] | 0.975125039 | 0.953310696 | 0.9 | 0.98816568 |
| Stepglm[both]+Ridge | 0.97093495 | 0.968590832 | 0.888888889 | 0.99852071 |
| Stepglm[backward]+Ridge | 0.97093495 | 0.968590832 | 0.888888889 | 0.99852071 |
| Stepglm[both]+plsRglm | 0.973295564 | 0.971137521 | 0.894444444 | 0.99260355 |
| Stepglm[backward]+plsRglm | 0.973295564 | 0.971137521 | 0.894444444 | 0.99260355 |
| Stepglm[both]+Enet[alpha=0.9] | 0.975921746 | 0.943972835 | 0.9 | 0.985207101 |
| Stepglm[backward]+Enet[alpha=0.9] | 0.97586273 | 0.943972835 | 0.9 | 0.985207101 |
| Stepglm[both]+Enet[alpha=0.1] | 0.975700438 | 0.943123939 | 0.9 | 0.986686391 |
| Stepglm[backward]+Enet[alpha=0.1] | 0.9757447 | 0.945670628 | 0.9 | 0.98816568 |
| Stepglm[both]+Enet[alpha=0.8] | 0.975877484 | 0.943972835 | 0.9 | 0.986686391 |
| Stepglm[backward]+Enet[alpha=0.8] | 0.975847977 | 0.944821732 | 0.9 | 0.986686391 |
| Stepglm[both]+Enet[alpha=0.2] | 0.975788961 | 0.943123939 | 0.9 | 0.986686391 |
| Stepglm[backward]+Enet[alpha=0.2] | 0.975788961 | 0.943123939 | 0.9 | 0.986686391 |
| Stepglm[both]+Lasso | 0.975892238 | 0.94057725 | 0.9 | 0.983727811 |
| Stepglm[backward]+Lasso | 0.975966007 | 0.943972835 | 0.9 | 0.985207101 |
| Stepglm[both]+Enet[alpha=0.6] | 0.975759454 | 0.944821732 | 0.9 | 0.986686391 |
| Stepglm[backward]+Enet[alpha=0.6] | 0.975818469 | 0.946519525 | 0.9 | 0.986686391 |
| glmBoost+GBM | 0.991575562 | 0.960101868 | 0.861111111 | 0.970414201 |
| Stepglm[both]+Enet[alpha=0.7] | 0.975906992 | 0.943123939 | 0.9 | 0.986686391 |
| Stepglm[backward]+Enet[alpha=0.7] | 0.975877484 | 0.943123939 | 0.9 | 0.986686391 |
| Lasso+Stepglm[backward] | 0.975921746 | 0.937181664 | 0.9 | 0.983727811 |
| Stepglm[both] | 0.975921746 | 0.937181664 | 0.9 | 0.983727811 |
| Stepglm[backward] | 0.975921746 | 0.937181664 | 0.9 | 0.983727811 |
| glmBoost+Stepglm[both] | 0.97428407 | 0.946519525 | 0.9 | 0.98816568 |
| glmBoost+Stepglm[backward] | 0.97428407 | 0.946519525 | 0.9 | 0.98816568 |
| Stepglm[both]+Enet[alpha=0.4] | 0.975803715 | 0.943123939 | 0.9 | 0.986686391 |
| Stepglm[backward]+Enet[alpha=0.4] | 0.975803715 | 0.943123939 | 0.9 | 0.986686391 |
| Stepglm[both]+Enet[alpha=0.3] | 0.975803715 | 0.943123939 | 0.9 | 0.986686391 |
| Stepglm[backward]+Enet[alpha=0.3] | 0.975847977 | 0.946519525 | 0.9 | 0.98816568 |
| Stepglm[both]+glmBoost | 0.968633353 | 0.965195246 | 0.894444444 | 0.99852071 |
| Stepglm[backward]+glmBoost | 0.968633353 | 0.965195246 | 0.894444444 | 0.99852071 |
| Stepglm[both]+Enet[alpha=0.5] | 0.97586273 | 0.942275042 | 0.9 | 0.985207101 |
| Stepglm[backward]+Enet[alpha=0.5] | 0.975803715 | 0.946519525 | 0.9 | 0.986686391 |
| glmBoost+RF | 0.997580372 | 0.969439728 | 0.85 | 0.983727811 |
| RF | 0.998509863 | 0.974533107 | 0.877777778 | 0.99704142 |
| Lasso+GBM | 0.994659113 | 0.960101868 | 0.877777778 | 0.974852071 |
| RF+GBM | 0.993390283 | 0.953310696 | 0.872222222 | 0.970414201 |
| GBM | 0.994231252 | 0.959252971 | 0.883333333 | 0.974852071 |
| Stepglm[both]+SVM | 0.925375116 | 0.777164686 | 0.733333333 | 0.826923077 |
| Stepglm[backward]+SVM | 0.925375116 | 0.777164686 | 0.733333333 | 0.826923077 |
| Lasso+RF | 0.998288555 | 0.9762309 | 0.877777778 | 0.99704142 |
| Stepglm[both]+GBM | 0.993582083 | 0.951612903 | 0.855555556 | 0.968934911 |
| Stepglm[backward]+GBM | 0.993375529 | 0.953310696 | 0.861111111 | 0.967455621 |
| Stepglm[both]+RF | 0.997550864 | 0.966044143 | 0.861111111 | 0.98964497 |
| LDA | 0.969253013 | 0.959252971 | 0.9 | 0.99852071 |
| glmBoost+LDA | 0.965859632 | 0.965195246 | 0.894444444 | 0.99852071 |
| RF+LDA | 0.967202231 | 0.963497453 | 0.894444444 | 0.99852071 |
| Stepglm[both]+LDA | 0.966228478 | 0.956706282 | 0.894444444 | 0.99852071 |
| Stepglm[backward]+LDA | 0.966228478 | 0.956706282 | 0.894444444 | 0.99852071 |
| Lasso+LDA | 0.969400552 | 0.957555178 | 0.9 | 0.99852071 |
| Stepglm[backward]+RF | 0.997624633 | 0.96179966 | 0.866666667 | 0.98964497 |
| XGBoost | 0.992652592 | 0.884974533 | 0.786111111 | 0.938609467 |
| Lasso+XGBoost | 0.981122472 | 0.879881154 | 0.691666667 | 0.899408284 |
| glmBoost+XGBoost | 0.977795482 | 0.907470289 | 0.727777778 | 0.963757396 |
| RF+XGBoost | 0.971952965 | 0.856112054 | 0.733333333 | 0.903106509 |
| Stepglm[both]+XGBoost | 0.992770622 | 0.929966044 | 0.777777778 | 0.961538462 |
| Stepglm[backward]+XGBoost | 0.989251833 | 0.929966044 | 0.819444444 | 0.977071006 |
| NaiveBayes | 0.951017277 | 0.977928693 | 0.880555556 | 1 |
| Lasso+NaiveBayes | 0.951976276 | 0.9762309 | 0.869444444 | 1 |
| glmBoost+NaiveBayes | 0.960031868 | 0.970288625 | 0.855555556 | 1 |
| RF+NaiveBayes | 0.955827026 | 0.9762309 | 0.869444444 | 1 |
| Stepglm[both]+NaiveBayes | 0.960533499 | 0.969439728 | 0.838888889 | 1 |
| Stepglm[backward]+NaiveBayes | 0.960533499 | 0.969439728 | 0.838888889 | 1 |
| Stepglm[both]+RF+NaiveBayes | 0.997595125 | 0.966044143 | 0.861111111 | 0.985207101 |
| Lasso+GBM+RF | 0.99457059 | 0.959252971 | 0.872222222 | 0.973372781 |
| Stepglm[both]+Enet[alpha=0.8]+XGBoost | 0.975980761 | 0.942275042 | 0.9 | 0.985207101 |
| Stepglm[backward]+Enet[alpha=0.8]+glmBoost | 0.975877484 | 0.943972835 | 0.9 | 0.986686391 |
| Stepglm[both]+Enet[alpha=0.2]+GBM | 0.975759454 | 0.943123939 | 0.9 | 0.986686391 |
| Stepglm[backward]+Enet[alpha=0.2]+XGBoost | 0.975818469 | 0.943123939 | 0.9 | 0.986686391 |
| Stepglm[both]+Lasso+GBM | 0.975966007 | 0.943972835 | 0.9 | 0.985207101 |
| Stepglm[backward]+Lasso+RF | 0.975951253 | 0.943972835 | 0.9 | 0.985207101 |
| Stepglm[both]+Enet[alpha=0.6]+GBM | 0.975877484 | 0.943972835 | 0.9 | 0.986686391 |
| Stepglm[backward]+Enet[alpha=0.6]+GBM | 0.975906992 | 0.943123939 | 0.9 | 0.986686391 |
| glmBoost+GBM+Lasso | 0.993050945 | 0.960101868 | 0.855555556 | 0.968934911 |
| Stepglm[both]+Enet[alpha=0.7]+GBM | 0.975877484 | 0.943972835 | 0.9 | 0.986686391 |
| Stepglm[backward]+Enet[alpha=0.7]+NaiveBayes | 0.975921746 | 0.943972835 | 0.9 | 0.986686391 |
| Lasso+Stepglm[backward]+Enet[alpha=0.5] | 0.975921746 | 0.937181664 | 0.9 | 0.983727811 |
